# Supplementary material for: CoFeBP Micro Flowers (MFs) for Highly Efficient Hydrogen Evolution Reaction and Oxygen Evolution Reaction Electrocatalysts
Source: Nanomaterials (Basel). 2024 Apr 17;14(8):698. doi: 10.3390/nano14080698 (PMC11053626; doi:10.3390/nano14080698)
Supplement: Supplementary file 1 [file nanomaterials-14-00698-s001.zip › nanomaterials-2952624-supplementary.pdf]

Supporting Information

CoFeBP micro flowers (MFs) for highly efficient hydrogen evolution reaction and oxygen evolution reaction electrocatalysts

Shusen Lin, Shalmali Burse, Rutuja Mandavkar, Md Ahasan Habib, Mehedi Hasan Joni, Jae-Hun Jeong*, Young-Uk Chung** and Jihoon Lee***

Department of Electronic Engineering, College of Electronics and Information, Kwangwoon University, Nowon-gu, Seoul, 01897, South Korea.

Correspondence e-mail: myloveofjh@gmail.com (J. Jeong) *, yuchung@kw.ac.kr (Y. Chung) **, jihoonlee@kw.ac.kr (J. Lee) **

S1. Detailed experimental section

S1.1. Preparation of substrate and precursors

S1.2. Electrochemical characterizations

S1.3. Morphological, elemental, and optical characterizations

S1.4. Reference electrode fabrication

S1.5. Fabrication of CoFeBP micro flower branch (MFB)

S1.6. Raman analysis on CoFeBP MFBs after post-annealing

S1.7. XRD analysis on CoFeBP MFBs

S1.8. Turnover frequency (TOF) calculation for CoFeBP MFBs

S1.9. Faradaic efficiency calculation

S1.10. XPS analysis on CoFeBP MFBs

S1.11. EIS & LSV analysis before/after annealing

S2. CoFeBP MFB electrocatalysts

S2.1. Substrate and other necessary characterizations

S2.1.1. Bare Ni foam characterization

S2.1.2. LSV at different scan rates with the best CoFeBP

S2.1.3. EIS voltage variation of the best CoFeBP MFBs

S2.1.4. Pt/C (HER) reference electrode

S2.1.5. RuO_2 (OER) reference electrode

S2.2. Reaction parameter and precursor optimizations

S2.2.1. Fabrication steps of CoFeBP MFB

S2.2.2. Reaction duration optimization

S2.2.3. Reaction temperature optimization

S2.2.4. B-P concentration optimization

S2.2.5. Co-Fe concentration optimization

S2.2.6. Urea concentration optimization

S2.2.7. Urea- Ammonium fluoride concentration optimization

S2.3. Post-annealing optimization

S2.3.1. Post-annealing time optimization

S2.3.2. Post-annealing temperature optimization

S2.4. Before and after post-annealing analysis of CoFeBP MFB

S2.4.1. Before and after post-annealing: Raman

S2.4.2. Before and after post-annealing: XRD

S2.4.3. Before and after post-annealing: EIS

S2.4.4. Before and after post-annealing: HER/OER LSV

S3. Analysis on the best CoFeBP MFB

S3.1. XRD spectra of CoFeBP and related compounds

S3.2. XRD PDF cards of related compounds

S3.3. CV measurement in the same range of OER

S3.4. Water-gas displacement for Faradaic efficiency

S3.5. Faradaic efficiency of the best CoFeBP MFBs

S3.6. 3-E CA and LSV comparison

S3.7. 2-E CA and LSV comparison

S3.8. 2-E stabiltiy test in 1 M and 6 M KOH

S3.9. 2-E LSV in sea and river waters

S3.10. 2-E CA and LSV comparison in seawater + 1M KOH

S4. After stability test

S4.1. After stability test: SEM

S4.2. After stability test: Raman

S4.3. After stability test: LSV

S1. Detailed experimental section

S1.1. Preparation of substrate and precursors

S1.2. Electrochemical characterizations

S1.3. Morphological, elemental, and optical characterizations

S1.4. Reference electrode fabrication

S1.5. Fabrication of CoFeBP micro flower branch (MFB)

S1.6. Raman analysis on CoFeBP MFBs after post-annealing

S1.7. XRD analysis on CoFeBP MFBs

S1.8. Turnover frequency (TOF) calculation for CoFeBP MFBs

S1.9. Faradaic efficiency calculation

S1.10. XPS analysis on CoFeBP MFBs

S1.11. EIS & LSV analysis before/after annealing

S1. Detailed experimental section

S1.1. Preparation of substrate and precursors

Nickel foam (NF) was utilized as a substrate for the fabrication of CoFeBP micro-flower-branch (MFB) electrodes due to the high 3-dimensional porosity.[1] The porous structure can lead to the large surface area and improved electrocatalytic efficiency as compared with other flat substrates.[1] Bare NF of 3×2 cm^2 was ultrasonicated in 6 M hydrochloric acid (HCl) for 15 min to remove the surface oxides and contaminants. Then, the NF was washed in ethanol and DI water to eliminate the chemical residuals and then dried in ambient. The surface morphology and elemental analyses on bare NF are provided in Figs. S1(a) – S1(c), which showed clean 3-D porous NF ready for the CoFeBP fabrication. The electrochemical performance of bare NF is shown in Figs. S1(d) – S1(e), in which the HER performance was 466 mV at 100 mA/cm^2 and that of OER was 861 mV at 100 mA/cm^2.

The CoFeBP precursor solution was consisted of cobalt(II) nitrate hexahydrate ($Co(NO_3)_2{\bullet}6H_2O$) for Co, iron(III) nitrate nonahydrate ($Fe(NO_3)_3{\bullet}9H_2O$) for Fe, boric acid (H_3BO_3) for B, and sodium hypophosphite ($H_2NaO_2P{\bullet}H_2O$) for P as precursors, which were dissolved in 30 mL DI water for a hydrothermal reaction as illustrated in Fig. S6. In addition, the urea (CH_4N_2O) and ammonium fluoride (NH_4F) were adapted as surface active agents to boost the nucleation and 3D structure formation by increasing solution conductivity. The precursor solution was magnetically stirred for 10 min to get a homogeneous mixture. Specific precursor concentrations and molarities are listed in the related figures and sections. All the chemicals were analytical grades and used as received without further purification (Sigma Aldrich, USA). Potassium hydroxide (KOH), sulfuric acid (H_2SO_4) and phosphate-buffered saline (PBS) tablets were dissolved into 30 mL DI water respectively to make 1 M KOH (alkaline), 0.5 M H_2SO_4 and 1 M PBS (neutral) solutions for electrochemical performance measurements in different pH waters. The river water was collected

from the Han River (Seoul, Korea) and the seawater was obtained from the Yellow Sea (Incheon, Korea) respectively. Natural waters were used after the sedimentation and filtration.

S1.2. Electrochemical characterizations

Three-electrode (3-E) electrochemical performance was examined by an electrochemical workstation (Wizmac, South Korea), which was consisted of the CoFeBP micro flower branch (MFB) electrode as a working electrode, Ag/AgCl as a reference electrode and graphite as a contour electrode. The applied LSV potential was from - 0.6 V to 0.2 V vs. RHE for the HER and from 1.2 V to 2.0 V vs. RHE for the OER respectively. All the electrochemical measurements were performed with the fixed cell position, indicating fixed electrode distance and measurement error was tried to be minimized for consistency. The polarization curves were plotted as received without an iR correction. iR correction can improve the LSV performance by removing the solution resistance. In the reaction cell, both solution resistance and contact/intrinsic resistance exist, which is not avoidable during the splitting process. [2] The reversible hydrogen electrode (RHE) potential was converted based on Eq. (S1).

$$E\ [\text{V vs RHE}] = E(\text{Ag/AgCl}) + 0.1971 + 0.059 \times \text{pH} \qquad \text{(S1)}$$

The polarization curve was collected by linear sweep voltammetry (LSV) measurement at the scan rate of 5 mA/s. As an example, the scan rate varied between 2, 5, 8 and 10 mV/s for the HER and OER LSV measurement in Fig. S2.

$$\text{Current response, } i_{mp}\,(\text{A}) = 0.446 nFAC^{o}\,(nF\upsilon D_0/RT)^{1/2} \qquad \text{(S2)}$$

Where n is the number of electrons transferred in the redox event, A is the electrode surface area (geometric surface area), D_0 is the diffusion coefficient of the oxidized analyte, and C^{o} is the bulk concentration of the analyte. Additionally, R ($JK^{-1}mol^{-1}$) is the gas constant and T (K) is the temperature. As per the Randle-Saiki equation, the peak current i_p (A) can increase linearly with the square root of the scan rate (V/s). The higher scan rate can lead to a reduction in the diffusion layer

and thus can result in high current densities with lower potential [3]. The higher scan rate not only reduces the diffusion layer but also alters the gas diffusion activity, which may affect the overall catalytic activity.[4] At the same time, at higher scan rates, reaction kinetics decreases with the degradation of performance.[5] The 5 mV/s scan rate is adapted for LSV in this experiment and other previous works widely adapt the scan rates of ~ 5 mV/s.

Electrochemical impedance spectroscopy (EIS) was carried out at 50 mA/cm^2 corresponding voltages between 100 kHz to 0.1 Hz with an amplitude of 5 mV. The EIS measurements presented voltage-dependent R_{ct} response around the turnover regions as seen in Fig. S4. For example, a lower R_{ct} values were obtained at a higher voltage due to reduction in the diffusion depth.[6] Thus, the potential of EIS measurement was picked at the fixed current of 50 mA/cm^2 to properly reflect the R_{ct} trend in a set. The cyclic voltammetry (CV) was obtained at various scan rates between 40 and 180 mV/s in the non-faradic potential range of 0.2 ~ 0.3 V vs. RHE for the HER and 1.04 ~1.14 V vs. RHE for OER. The electrochemical double-layer capacitance (C_{dl}) plots are obtained from the anodic and cathodic current density plots: $\Delta jH_{0.25} = (J_a - J_c)/2$ and $\Delta jO_{1.09} = (J_a - J_c)/2$, where J_a is the anodic current and J_c is cathodic current.[7] The C_{dl} values are obtained by extracting the slope of C_{dl} plots, which represent the electro chemical surface area (ECSA) of an electrode. For HER, the CV curves were taken in a non-faradic region where there are no charge-transfer reactions occur in between 0.2 and 0.3 V below 1.023 V based on Eq. (S3)

$$E_{RHE} = E + 0.059 * pH + 0.197 \text{ (Ag/AgCl)} \qquad \text{(S3)}$$

The actual reverse sweeping voltage (E) was between - 0.72– and - 0.823 V. For OER, the CV curves were taken in between 1.04 and 1.14V below 1.23 V based on the same equation. The actual forward sweeping voltage (E) was between 0.017 and 0.117 V. The ECSA calculation formula is as follows: ECSA ≈ A × C_{dl} / Cs, where A is geometrical area (0.5×0.5 cm^2) and Cs is specific capacitance of smooth electrode surface that generally is 0.04 mF cm^{-2} in 1M KOH.[8]

The performance of CoFeBP was tested in both laboratory and industrial conditions. The laboratory condition was at room temperature in 1M KOH. For the industrial condition, the electrolyte was changed to 6M KOH, and the electrolysis was performed on the hotplate with the temperature at 60 °C. The measurement started when the electrolyte temperature stabilized the setting values.

S1.3. Morphological, elemental, and optical characterizations

Scanning electron microscope (SEM, Regulus 8230 Hitachi, Japan) and energy-dispersive X-ray spectrometer (EDS, Ultimax, Oxford Instruments, UK) were utilized to analyze the surface morphology and elemental phases of various CoFeBP micro flower branch (MFB) electrodes. SEM scanning was conducted using the accelerating voltage of 20 keV, the beam size of 11 and working distance of 10 mm. Regarding EDS analysis, the accelerating voltage was set as 10 keV. The beam size of 16 and working distance of 15 mm were adapted respectively. Raman analysis was performed by a NOST system (Nostoptiks, South Korea), equipped with 532 nm laser, spectrograph (ANDOR SR-500, UK) and charge-coupled device (CCD). The laser power was 120 mW and the slit width was set as 100 μm. The exposure time of 0.8 s was used along with 10 times accumulations for the Raman signal collection. X-ray diffraction (XRD, D8 Advance, Bruker, USA) was utilized to analyze the crystalline phases and planes of MFB electrodes at a scanning rate of 2° /min by the Cu Kα radiation (λ = 1.5406 Å). X-ray photoelectron spectroscopy (XPS) was adapted to understand the chemical state and electronic structure of CoFeBP MFBs at 1486.6 eV under < 10^{-8} torr with the X-ray spot size of 10 μm of Al K-Alpha X-ray source (FC-XP10, Thermo Fisher Scientific, USA). The anode line was monochromatized and the spectrum was measured at 0.05 eV intervals.

S1.4. Reference electrode fabrication

In this work, the Pt/C reference electrode was adapted for the HER reference and the RuO_2 was used as OER benchmark electrode. For the Pt/C HER reference electrode fabrication, 20 mg of Pt/C and

60 μL of 5 wt% Nafion (117 solution, Aldrich) were dispersed into a mixed solution containing of 5 mL ethanol and 5 mL DI water [9]. The precursor solution was then ultrasonicated for 30 min for a homogeneous dispersion. A clean NF was soaked into the solution to make Pt/C reference electrode for 30 min and dried in ambient. The OER benchmark electrode of RuO_2 was fabricated analogously by a soaking approach. 40 mg of RuO_2 and 60 μL of 5 wt% Nafion (117 solutions, Aldrich) were dispersed in the 5 mL ethanol and 5 mL DI water solution [9] and then ultrasonically mixed for 30 min. The NF was then immersed in the dispersed solution for 30 min before being dried in ambient. The surface morphology, EDS spectrum and electrochemical properties of Pt/C and RuO_2 reference electrodes are provided in Figs. S4 – S5.

S1.5. Fabrication of CoFeBP micro flower branch (MFB)

The general fabrication process of CoFeBP micro flower branch (MFB) electrode on the NF by a hydrothermal reaction is illustrated in Fig. S6, including the hydrothermal reaction and annealing optimization. In short, a clean NF was dipped into the CoFeBP precursor solution in a Teflon-line autoclave and then various hydrothermal reaction parameters, i.e., temperature, duration, and precursor concentration, were thoroughly optimized by the systematic control on each parameter. After the hydrothermal reaction, the temperature was cooled down to an ambient temperature and the sample was rinsed in DI water. Post-annealing was performed to improve crystallinity in a rapid thermal processing (RTP) system as illustrated in Fig. S6. Then, the sample was cut into 1 × 1 cm^2 for various characterizations and analyses. The synthesis of CoFeBP MFB electrode can be described as below.

$$Co(NO_3)_2{\bullet}6H_2O + Fe(NO_3)_3{\bullet}9H_2O + H_3BO_3 + 2NaH_2PO_2{\bullet}H_2O + CO(NH_2)_2 + NH_4F + n.H_2O \rightarrow \quad (S4)$$

$$Co^{2+} + Fe^{3+} + 5(NO_3)^- + [B(OH)_4]^- + H^+ + PH_3 + 2Na^+ + HPO_4^{2-} + 17H_2O + CO_2(g) + 3NH_4^+ + 2OH^- + F^- \rightarrow \quad (S5)$$

$$Co(NO_3)_2{\bullet}6H_2O \rightarrow Co^{2+} + 2(NO_3)^- + 6H_2O \quad (S5\text{-}1)$$

$Fe(NO_3)_3{\bullet}9H_2O \rightarrow Fe^{3+} + 3(NO_3)^{-} + 9H_2O$ (S5-2)

$H_3BO_3 + H_2O \rightarrow [B(OH)_4]^{-} + H^{+}$ (S5-3)

$2NaH_2PO_2{\bullet}H_2O \rightarrow PH_3 + Na_2HPO_4 + 2H_2O \rightarrow PH_3 + 2Na^{+} + HPO_4^{2-} + 2H_2O$ (S5-4)

$CO(NH_2)_2 + H_2O \rightarrow CO_2(g) + 2NH_3(g)$

$2NH_3(g) + 2H_2O \rightarrow 2NH_3{\bullet}H_2O(aq) \rightarrow 2NH_4^{+} + 2OH^{-}$ (S5-5)

$NH_4F \rightarrow NH_4^{+} + F^{-}$ (S5-6)

$CoFeBP + 5(NO_3)^{-} + 2Na^{+} + 21H_2O + CO_2(g) + 3NH_4^{+} + 2OH^{-} + F^{-}$ (S6)

The precursors and surface agents utilized for the fabrication of CoFeBP micro flower branch (MFB) electrode are shown in Eq. (S4) above and the ionized precursors are shown in Eq. (S5). The ionized precursors are formed by the ionic dissolution and reactions of each element as seen in Eqs. (S5-1) - (5-6). For example, the Co^{2+} and Fe^{3+} ions are dissolved from the Co and Fe precursors of $Co(NO_3)_2{\bullet}6H_2O$ and $Fe(NO_3)_3{\bullet}9H_2O$ as seen in Eqs. (S5-1) and (5-2). The $[B(OH)_4]^{-}$ as the B precursor is generated from the H_3BO_3 as seen in Eq. (S5-3). Sodium hypophosphite (NaH_2PO_2) can decompose and form the PH_3 and NaH_2PO_2 as seen in Eq. (S5-4). Na_2HPO_4 further continue to ionize and leave $2Na^{+}$ + HPO_4^{2-} in the solution [10]. Urea ($CO(NH_2)_2$) and ammonium fluoride (NH_4F) served as surface agents to modulate the nucleation rate in Eqs. (S5-5) - (5-6). Both surface agents led to a high-speed homogeneous deposition by raising conductivity [11][12]. The urea decompose into NH_3 and CO_2, where the NH_3 group can accept a proton and generate hydroxyl (OH^{-}) ions, a common hydrophilic group, as seen in Eq. (S5-5) [11]. The generation of OH^{-} can increase the number of precursors binding to the nickel foam (NF) and improve the electrodes' ability to absorb water molecules.[11] The presence of F ions (F^{-}) ($NH_4F \rightarrow NH_4^{+} + F^{-}$) can readily chemisorb elements with dangling bonds and promote the nucleation process with strong electronegativity[13]. Finally, various 3-D CoFeBP micro flower branch (MFB) structures can be gradually developed during the hydrothermal reaction as seen in Eq. (S6).

S1.6. Raman analysis on CoFeBP MFBs after post-annealing

Generally, Raman spectroscopy is a powerful tool in the field of vibrational spectroscopy in a molecular system and the obtained Raman peaks can define the unique phonon scattering of lattice vibrations.[7] It also yields useful results in the analysis of surface crystallinity and crystal quality of nano- and micro-structures.[7] The Raman peaks of chemically synthesized CoFeBP electrode before annealing treatment is shown as black line in Fig. S29. Several strong characteristic Raman bands were observed at 596, 962, 1029 and 1102 cm^{-1} and other smaller peaks appeared at 261, 360 and 478 cm^{-1}. In previous work, the Fe-P bond exhibited strong vibration peak at 476 cm^{-1} [14] and CoP_3 possess several sharp peaks between 200 and 600 cm^{-1}.[15] Three peaks of 513, 605 and 660 cm^{-1} were found in CoOOH.[16] The Co_3O_4 showed the strong E_g and E^1_{2g} vibration modes at 478 and 569 cm^{-1} and Fe-O showed a characteristic peak around 261 cm^{-1}.[17–19] After comparison, the Raman peaks of CoFeBP at 261, 478 and 596 cm^{-1} can be assigned to the Fe-O bond, E_g vibration mode of Co_3O_4 and CoOOH respectively. As for the stretching vibration peaks in 950 ~ 1100 cm^{-1}, it is possibly due to active oxygen bond (O-O). The OH^- species initially adsorbed on Co and Fe surface and it can be converted into M-O* (M=Co and Fe).[20,21] In this regard, Co-O* and nearby Co-O* or Fe-O* can directly form O-O bonds without consuming much energy.[20,21] The peak can split into two shoulder peaks which can be related to the symmetric stretching O-O vibration.[20,21] The Raman spectrum after the post-annealing is provided as blue line in Fig. S32 and intensified peaks or increased peak intensities were obtained after the vacuum annealing process. The moderate annealing treatment can effectively improve the crystalline. Initially, crystal atomic arrangement was randomly grown on the surface of Ni foam and various defects may exist.[22,23] Surface atomic diffusion initiated by thermal treatment can effectively alter the spatial atomic arrangement and hence can reduce the atomic dislocation density as well as the surface defects.[22,23]. Ramana analysis on the samples at higher annealing temperature are seen in Fig. 2(g). The peak intensity gradually decreased from 200 to 500 ºC. Especially at 500 ºC, the main peaks were nearly not visible. Higher annealing temperature can result in lattice distortion and even undesirable crystal sintering[24]

as revealed from the SEM images. As a result, lower intensity peaks were obtained, and the trend of electrochemical properties was consistent with the Raman analysis as seen in Fig. 3.

Figure S44 shows the Raman analysis on the CoFeBP MFBs after the 12-hr stability test. As the stability test was done in the 2-electrode configuration, both anode and cathode are examined. Overall, the intensity of characteristic peaks became lower in both anode and cathode and cathode exhibited further reduced peak intensity after a long-time redox reaction as compared with the electrode before the stability test. It mainly can be due to the amorphous surface oxidation process leading to the decreased quality of surface crystals and the loss of local crystallinity.[25] The peaks at 478 and 569 cm^{-1} possibly can be assigned to the E_g and E^1_{2g} vibration modes of Co_3O_4 due to the stronger oxidation during the HER/OER.[17] The sharper peak ranging from 880 to 1190 cm^{-1} in the anode side might be explained by more O-O couples formation during oxygen and hydrogen generation process.[20,21]

S1.7. XRD analysis on CoFeBP MFBs

Generally, XRD patten provides the crystalline phase and the elemental composition can be referred to the diffraction pattern. Figures S33(a) – S33(d) show the X-ray diffraction (XRD) patterns of CoFeB, CoBP, CoFeP and CoFeBP electrodes. The CoFeB, CoBP and CoFeP were additionally fabricated for a comparison with the CoFeBP. Here, the CoFeBP electrode exhibited strong peaks between 27 and 35 such as 27.0, 27.9, 30.0, 34.0, 35.2 º as well as other secondary small peaks in the scan range in Fig. S33(a). Several small peaks were observed at 27.7, 28.2, 32.8 and 33.9 º for the Co-Fe-P electrode, and a series of multiple secondary peaks were also observed throughout the scan range in Fig. S33(b). Co-Fe-B exhibited multiple strong peaks such as 26.7, 30.1, 33.2, 35.5, 38.7, 59.2 and 63.0 º in Fig. S33(c). In the case of CoBP, more peaks were obtained such as 24.0, 27.9, 32.2, 34.3, 36.1, 37.2, 38.7, 39.9, 49.3 and 57.7 º in Fig. S33(d). The two main strong diffraction

peaks at ~ 43 and 52° in all XRD patterns can be indexed to the (111) and (200) planes of Ni foam substrate [26].

The CoFeBP is a new material composition and thus there was no exactly matching PDF card in the database (http://icsd.kisti.re.kr) and literature. The XRD patterns of related compounds are discussed in Figs. S34(a) – S34(d). In Fig. S34(a), the Co_2B (ICSD ID:14499) exhibits one strong peak at 59.6° and other secondary peaks in between 41 ~ 65 °. Similarly, the strongest peak is located at 21.6 ° and more secondary peaks appear the pattern of Co_3B (ICSD ID:53388). For CoB (ICSD ID:14502), stronger diffraction peaks can be witnessed at 34.2, 35.4, 39.5, 41.4, 44.1, 49.3 and 55.7 ° and fewer secondary peaks appear. In Fig. S34(b), CoP_3 (ICSD ID:8621) demonstrates several strong peaks located at 23.1, 32.8, 37.5 and 42.3° whereas the diffraction peaks of Co_2P (ICSD ID:9911) are primarily ranging in between 40 and 65 °. The strong peaks of CoP (ICSD ID:52318) can be observed from 23 to 36 ° and from 47 to 62 °. Figure S34(c) shows the FeB and BP-related XRD patterns. Here, the Fe_2B (ICSD ID:5145) presents two strong peaks located at 27.5 and 58.7 ° while the FeB (ICSD ID:14501) shows multiple intense peaks located at 32.6, 37.9, 41.3, 45.2, 47.8 and 63.2 °. In BP (ICSD ID:54947) spectrum, two main peaks appear at 39.9 and 57.6 °. The Fe-P-related XRD patterns are shown in Fig. S34(d). FeP_2 (ICSD ID:3866) demonstrates one main peak at 23.9 ° and other peaks are located from 31.7 to 63 °. In FeP_4 (ICSD ID:1485) spectrum, a series of small peaks can be found from 46.1 to 65 ° as well as a few strong peaks at 25.6, 31.8, 32.4 and 38.7 °. In FeP (ICSD ID:3890), the peak at 23.1° shows the highest intensity and other secondary peaks can be seen from 30.9 to 65°. Overall, there was not exactly matching XRD card with the CoFeBP XRD pattern from the closely related materials. Thus the unique XRD pattern of CoFeBP can indicate the formation of CoFeBP and a series of small secondary peaks may indicate the formation of a polycrystalline phase.[27] The polycrystalline phases can expose more active sites and the long-range disordered structure can be related to the higher structural flexibility in the modulation of

surface electronic structure as compared to the signal crystal phase.[25] Thus, the polycrystalline CoFeBP may demonstrate a rapid water splitting performance.

Figure S30 shows the XRD comparison before and after the post-annealing treatment. Before the thermal treatment, the whole XRD pattern is shown in Fig. S30(a): main peaks are shown in Fig. S30(a-1) and secondary peaks from 50 to 70 º can be found in the zoom-in image in Fig. S30(a-2). After annealing, several stronger peaks can be found at 22.8, 26.7, 27.6, 29.5, 33.7 and 35.1 º and additional secondary peaks were observed at 38.4, 41.0, 42.5º as seen in Figs. S30(b) – S30(b-1). In the zoom-in spectrum in Fig. S30(b-2), a series of small peaks appeared such as 55.7, 56.9, 57.4, 60.8, 61.7 and 66.5 º. The peaks between 26 and 42 º exhibited increased intensity without shifting, meaning improved crystallinity.[25] The annealing process can effectively reduce atomic dislocation and remove surface point defects and hence can improve the crystallinity.[25] The appearance of new peaks such as 22.8 º and others between 55 and 67 º may be mainly due to the changed local atomic arrangement via atomic diffusion initiated by the heat process.[25] It was concluded that hydrothermal synthesized CoFeBP electrodes indicated the polycrystal phase and the annealing can effectively improve the crystallinity.

S1.8. Turnover frequency (TOF) calculation for CoFeBP MFBs

The HER and OER turnover frequency (TOF) is defined as the number of hydrogen and oxygen generated during the turnover per unit time [28]. TOF can be used to compare the intrinsic active site efficiency of different catalysts and electrode's intrinsic capability of H_2 and O_2 generation [29]. The HER/OER TOF was calculated based on Eq. (S7) [30]:

$$\textbf{Turnover frequency (TOF)} = \frac{\frac{Total\ number\ of\ H^2\ or\ O^2}{\text{Geometric area (cm)}^2} \times \text{Current density}}{\text{Number of active sites}} \quad \text{(S7)}$$

Total H_2 turnover can be calculated by Eq. (S8):

$$= \left(\mathrm{j}\,\frac{\mathrm{mA}}{\mathrm{cm}^2}\right)\left(\frac{1\frac{\mathrm{C}}{\mathrm{s}}}{1000\ \mathrm{mA}}\right)\left(\frac{1\frac{\mathrm{mol}}{\mathrm{e}}}{96485\ \mathrm{C}}\right)\left(\frac{1\ \mathrm{mol\ H_2}}{2\frac{\mathrm{mol}}{\mathrm{e}}}\right)\left(\frac{6.022\times10^{23}\mathrm{mol\ H_2}}{1\ \mathrm{mol\ H_2}}\right) \quad \text{(S8)}$$

$$= 3.12 \times 10^{15} \left(\frac{H_2/s}{cm^2}\right) \text{ per } \left(\frac{mA}{cm^2}\right)$$

Total O_2 turnover can be calculated by Eq. (S9):

$$= \left(j\,\frac{mA}{cm^2}\right)\left(\frac{1\frac{C}{s}}{1000\text{ mA}}\right)\left(\frac{1\,\frac{mol}{e}}{96485\text{ C}}\right)\left(\frac{1\text{ mol }O_2}{4\,\frac{mol}{e}}\right)\left(\frac{6.022\times 10^{23}\text{mol }O_2}{1\text{ mol }O_2}\right) \quad \text{(S9)}$$

$$= 1.56 \times 10^{15} \left(\frac{O_2/s}{cm^2}\right) \text{ per } \left(\frac{mA}{cm^2}\right)$$

Active sites of CoFeBP MFBs:

$$= \frac{\text{EDS weight\%}}{100} \times \text{amonut of loading per area} \times \textit{Molecular mass} \times \left(\frac{\text{Avogadro Number}}{\text{Per concentration (mM)}}\right) \quad \text{(S10)}$$

$$= \frac{56.08}{100} \times 1.9\,\frac{mg}{cm^2} \times \frac{1\,mmol}{58.93\,mg} \times \frac{3.41}{100} \times 1.9\,\frac{mg}{cm^2} \times \frac{1\,mmol}{55.84\,mg} \times \left(\frac{6.022\times 10^{23}\text{ mM}}{1\text{ mM\^{}3}}\right)$$

$$= 1.2634 \times 10^{19}\ active\ sites.cm^{-2}$$

To calculate the TOF, the number of active sites should be calculated first. The loading material of 1.9 mg for the CoFeBP electrode is measured by weighing the bare Ni foam (0.0457 g) and CoFeBP MFB sample after the synthesis (0.0438 g). The molecular masses of Co (58.93 mg) and Fe (55.84 mg) were used for the calculation for the Co and Fe active sites. Further, the Wt.% of Co and Fe were evaluated from the EDS spectra for the annealing temperature variation set of CoFeBP electrodes.

$$TOF_{(HER)} = \frac{3.12\times 10^{15}\times 785}{1.2634\times 10^{19}}$$

$$TOF_{(HER)} = 0.193\ site^{-1}\ s^{-1}$$

Then, the HER TOF of CoFeBP MFB electrode (100 ºC) can be calculated as above. In the annealing temperature variation set, the 100, 200, 300 and 500 ºC electrodes demonstrated the HER current density of 785, 567, 520 and 500 mA/cm^2 at the fixed 400 mV.

$$TOF_{(OER)} = \frac{1.56\times 10^{15}\times 821}{1.2634\times 10^{19}}$$

$$TOF_{(OER)} = 0.101\ site^{-1}\ s^{-1}$$

Similarly, the OER TOF CoFeBP electrode (100 ºC) can be calculated as above. The OER current densities of 821, 572, 526 and 407 mA/cm^2 at 2.03 V were utilized for the calculations. The HER/OER TOFs are plotted in Figs. 3(j) and 3(k).

S1.9. Faradaic efficiency calculation

The faradaic efficiency is the ratio of experimentally generated H_2 or O_2 to theoretically calculated quantities [31], which can be calculated by Eq. (S11). [30]

$$\boldsymbol{Faradaic\ efficiecy} = \frac{experimental\ mol\ of\ O_2\ and\ H_2\ gas}{theoretica\ \ mol\ of\ O_2\ and\ H_2\ gas} \times 100 \qquad \text{(S11)}$$

The theoretical value of O_2 or H_2 can be calculated by Faraday's law based on Eq. (S12).

$$n = \frac{I \times t}{z \times F} \qquad \text{(S12)}$$

Where n the number of mol, I is the current in ampere, t is the time in seconds, z is the transfer of electrodes (z = 2 for H_2 and z = 4 for O_2). F presents the Faraday constant (96,485 C mol^{-1}).

Theoretical H_2 or O_2 quantities

The theoretical quantities of O_2 and H_2 were calculated at the current of 50 mA for 20 min as examples.

The theoretical value of O_2: n = 0.1555 mM.

The theoretical value of H_2: n = 0.3109 mM.

Experimentally measured H_2 or O_2 quantities

The generated gas was collected by the water-gas displacement method as seen in Fig. S36 and the O_2 or H_2 quantities were calculated by Eq. (S13):

$$PV = nRT \qquad \text{(S13)}$$

Where V is the volume of the collected gas. T is the temperature in kelvin, R is the ideal gas constant (0.0821 atm/mol K) and P is atmospheric pressure (~ 1 atm).

The H_2 amount in water-gas displacement:

$$(1\ atm)\ (0.0069\ L) = n\left(0.0821\frac{\mathrm{atm}}{\mathrm{mol}\ K}\right)(298K)$$

$$n = 0.2814\ \mathrm{mM}$$

The O_2 amount in water-gas displacement:

$$(1\ atm)\ (0.0034\ L) = n\left(0.0821\frac{\mathrm{atm}}{\mathrm{mol}\ K}\right)(298K)$$

$$n = 0.1402\ \mathrm{mM}$$

The faradaic efficiencies of H_2 and O_2 were determined to be 90.5% and 90.2% respectively as seen in Fig. S37.

S1.10. XPS analysis on CoFeBP MFBs

X-ray photoelectron spectroscopy (XPS) was conducted to probe the surface electronic states of Co, Fe, B and P. The full scan spectrum of the best sample after annealing is shown in Fig. 2(i) with the Co 2p, Fe 2p, B 1s and P 2p peaks and the high-resolution spectra of corresponding elements are presented in Figs. 2(i-1) – 2(i-4). In the Co 2p spectrum, peaks were observed at 778.5,780.8, 789.6, 793.6, 795.9 and 804.7 eV in Fig. 2(i-1). First, the binding energy (BE) of 793.6 and 778.5 eV can be assigned to Co $2p_{1/2}$ and $2p_{3/2}$. The standard BE of Co $2p_{1/2}$ is located at 793.3 eV and Co $2p_{3/2}$ was found at 778.3 eV according to the XPS handbook.[32] A positive shift of 0.3 and 0.2 eV for Co $2p_{1/2}$ and $2p_{3/2}$ was observed as compared with the pristine peak, indicating an electron donation and strong interaction with other atoms for the formation of CoFeBP. The peaks located at 795.9 and 781.4 eV indicate the existence of CoO on the surfac and BEs at 789.6 and 804.7 eV can be assigned to satellite peaks[19][33] In previous study, a positive shift of Co $2p_{3/2}$ was also observed with the N-FeCoNiS/SVG and Co-NiO@N-rich carbon nanofiber electrodes and a strong interaction with other constituent atoms were indicated.[34][35] The metallic Co^0, Co^{2+} and Co^{3+} were found at the BEs of 777.73, 797.06/780.91 eV and 799.51/782.82 eV respectively in the Co-Mo-B-P matrix.[33]

Similarly, the appearance of high Co-oxidation states was ascribed to the inevitable natural oxidation. The vibrational satellite peaks were also located at 789.8 and 804.5 eV for the $ZnCo_2O_4$@Co_3O_4.[36]

The Fe spectrum in Fig. 2(i-2) exhibited the characteristic peaks at 707.2, 708.6, 712.3, 715.1, 720.7, 723.6, 727.2 and 731.8 eV. The peaks at 707.2 and 720.7 eV can be assigned to Fe $2p_{3/2}$ and $2p_{1/2}$ respectively. The standard BEs of Fe $2p_{3/2}$ and $2p_{1/2}$ are found at 707.0 and 720.1 eV. As compared to the pristine Fe peak, a positive shift of 0.2 eV was observed for Fe $2p_{3/2}$ and similarly, Fe $2p_{1/2}$ peak also shifted towards higher BE with the shift of 0.6 eV, indicating electron donation process.[19,37] Electron donation can alert the charge distribution and can tune the electronic structure in the compound as indicated in the previous studies such as Fe-Ni_2P, Ir doped-FeNiP and Ni-Co-Fe-Se@NiCo-LDH.[38–41].The peaks at 708.6 and 723.6 eV can be related to the FeOOH and the peaks at 712.3 and 727.2 eV can indicate the formation of Fe_3O_4.[42] The satellite peaks were found at 731.8 and 715.1 eV[19,37].

On the other hand, the B 1s peak was negatively shifted by 2.4 eV from 189.4 to 187.0 eV in Fig. 2(i-3), indicating an orbital hybridization by electron acceptance in the formation of CoFeBP. The 192.5 eV peak can be assigned to the B_2O_3 oxidation states.[43] In the B-$MnFe_2O_4$, the B-O and B-M bonds were observed at 191.74 and 187.65 eV.[44] Three peaks of 128.7, 129.5 and 133.4 eV were observed in the P 2P spectrum in Fig. 2(i-4). The BE of P $2P_{3/2}$ and $2P_{1/2}$ was also negatively shifted by 1.2/1.24 eV toward lower BE from the standard 129.9/130.74, implying electron acceptance to the p-orbital. [33] The strong electronegativity and electron affinity can be beneficial for the strong bond formation in CoFeBP.[33] The 133.4 eV peak can belong to the oxidized P species (P-O) due to the atmospheric exposure.[33] The P oxidation states such as PO_4^{3-} and P_2O_5 are located at 133.2 and 134.6 eV and the obtained P-oxidation peak in CoFeBP is close to PO_4^{3-} species. The PO_4^{3-} was observed at a closed BE of 133.3 and 133.4 eV in other electrode such as Co-Mo-B-P/CF and Fe-doped NiCo-MoO_3[33][45]. Overall, the positive and negative shifts of elemental states

by the electron transfers from the Co and Fe to B and P can imply a strong ionic interaction of Co, Fe, B and P and also the oxidation states were identified.

S1.11. EIS & LSV analysis before/after annealing

Overall, the 100 ºC CoFeBP MFBs demonstrated the best electrochemical actives in the post-annealing temperature variation set, which can be mainly due to the improved crystallinity and effectively boosted carrier transport under the annealing condition.[46] The defect density can be altered by atomic diffusion and thermal treatment is crucial in the modulation of surface active sites.[9] As seen in the Nyquist plots in Fig. S31, lowered EIS values clearly revealed the reduced charge transfer resistance via the thermal treatment, which could be related to the faster HER/OER kinetics process. Improved HER/OER water splitting activities were observed after the annealing treatment in the polarization curves in Fig. S32, especially the HER and high current part of OER. The reduced oxidation peaks in OER might be due to the removal of oxygen vacancies after the annealing treatment.[47] While the oxidation peak might indicate additional active site for OER, the oxidation peak reduction might be beneficial for the improved interface kinetic transfer process. The oxygen removal can reduce the charge carrier trapping and recombination, allowing more carriers to attend in the water splitting process.[47]

S2. CoFeBP MFB electrocatalysts

S2.1. Substrate and other necessary characterizations

S2.1.1. Bare Ni foam characterization

S2.1.2. LSV at different scan rates with the best CoFeBP

S2.1.3. EIS voltage variation of the best CoFeBP MFBs

S2.1.4. Pt/C (HER) reference electrode

S2.1.5. RuO_2 (OER) reference electrode

S2.2. Reaction parameter and precursor optimizations

S2.2.1. Fabrication steps of CoFeBP MFB

S2.2.2. Reaction duration optimization

S2.2.3. Reaction temperature optimization

S2.2.4. B-P concentration optimization

S2.2.5. Co-Fe concentration optimization

S2.2.6. Urea concentration optimization

S2.2.7. Urea- Ammonium fluoride concentration optimization

S2.3. Post-annealing optimization

S2.3.1. Post-annealing time optimization

S2.3.2. Post-annealing temperature optimization

S2.4. Before and after post-annealing analysis of CoFeBP MFB

S2.4.1. Before and after post-annealing: Raman

S2.4.2. Before and after post-annealing: XRD

S2.4.3. Before and after post-annealing: EIS

S2.4.4. Before and after post-annealing: HER/OER LSV

S2.1.1. Bare Ni foam characterization

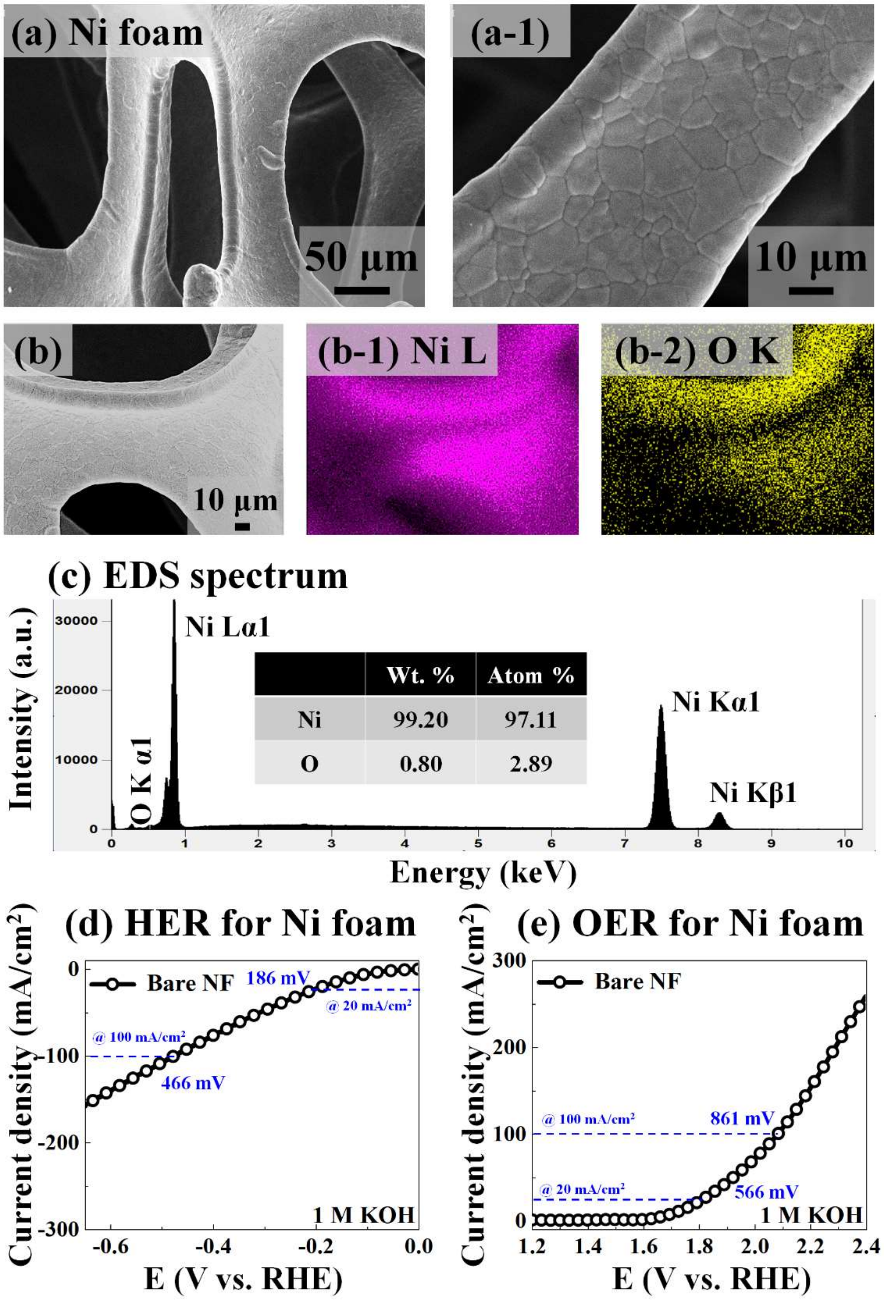

Figure S1: (a) – (a-1) Scanning electron microscope (SEM) images of porous nickel foam (NF). (b) – (b-2) SEM image and corresponding EDS phase maps of porous NF. (c) EDS spectrum with the atomic percentage. (d) & (e) hydrogen evolution reaction (HER) and oxygen evolution reaction (OER) activities of porous NF in 1M KOH. More details on the preparation of substrate and precursors can be found in the supplementary text S1.1.

S2.1.2. LSV at different scan rates for the best CoFeBP

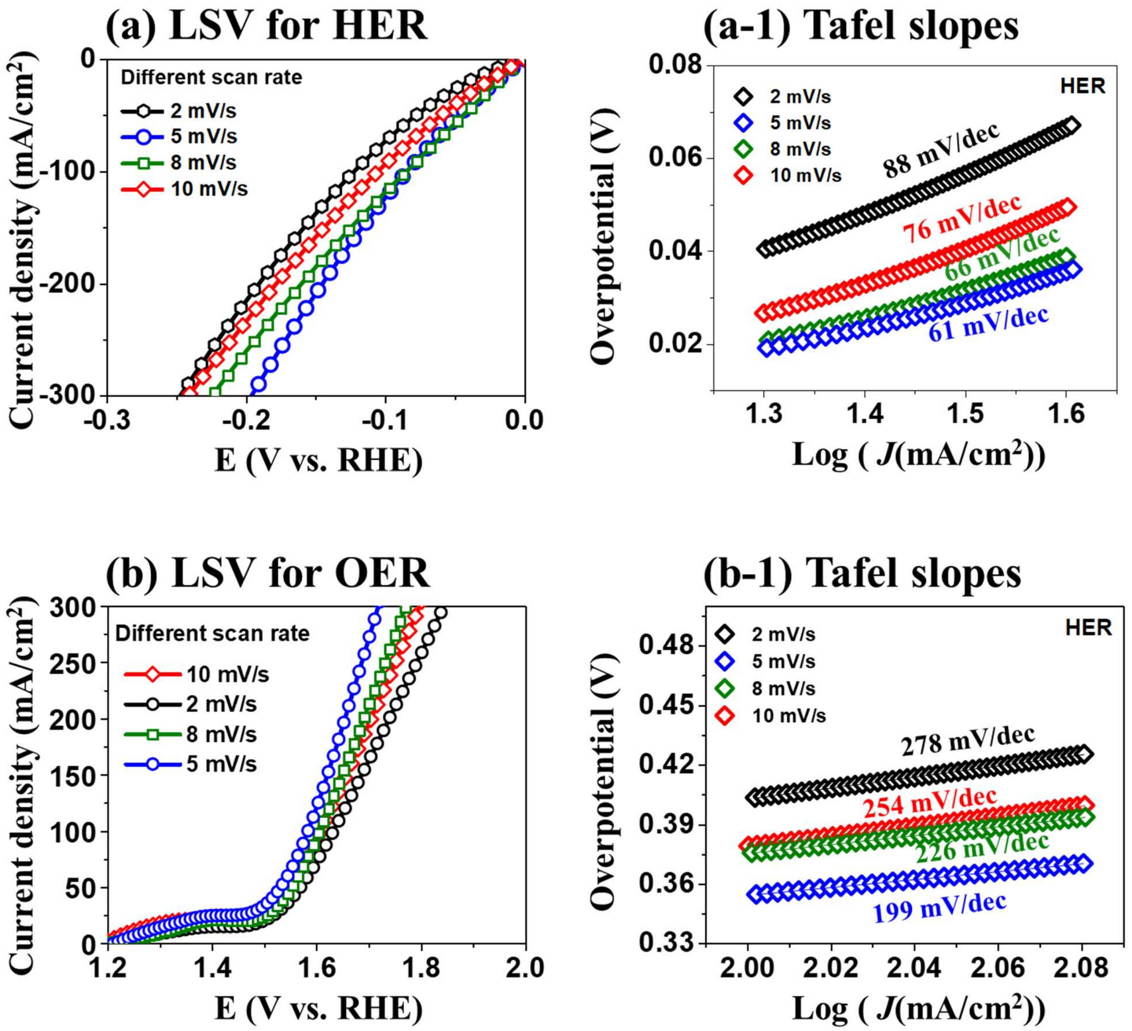

Figure S2: (a) & (b) hydrogen evolution reaction (HER) and oxygen evolution reaction (OER) activities of the best CoFeBP MFB at different scan rates between 2 ~ 10 mV/s. (a-1) & (b-1) Corresponding Tafel slopes. 5 mV/s demonstrated the optimized scan rate and thus it was adapted for further LSV measurements. More details can be found in the supplementary text S1.2.

S2.1.3. EIS voltage variation of the best CoFeBP MFBs

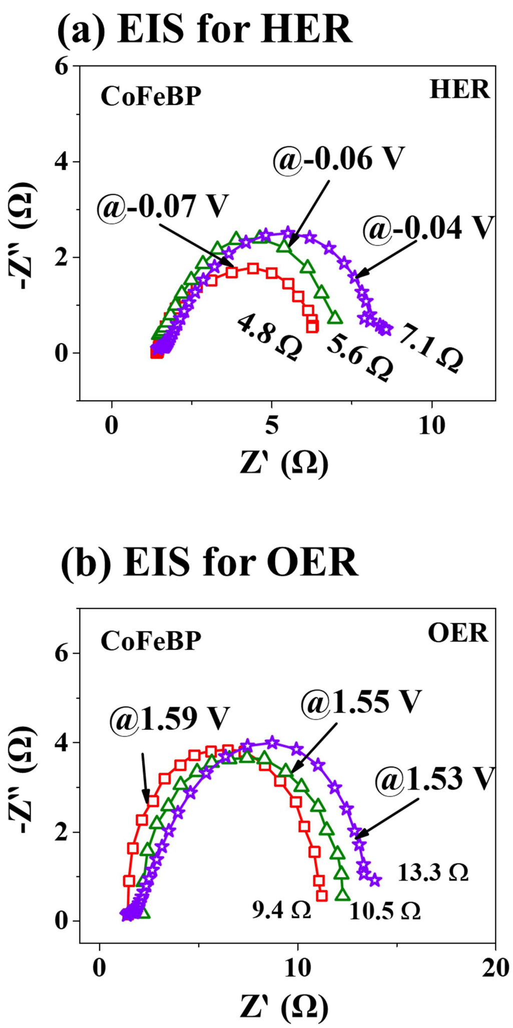

Figure S3: Electrochemical impedance spectroscopy (EIS) measurements for the optimized CoFeBP MFB electrode at different voltages. (a) HER EIS. (b) OER EIS. Voltage variation at fixed current was adapted for further EIS measurements. More details can be found in the supplementary text S1.2.

S2.1.4. Pt/C (HER) reference electrode

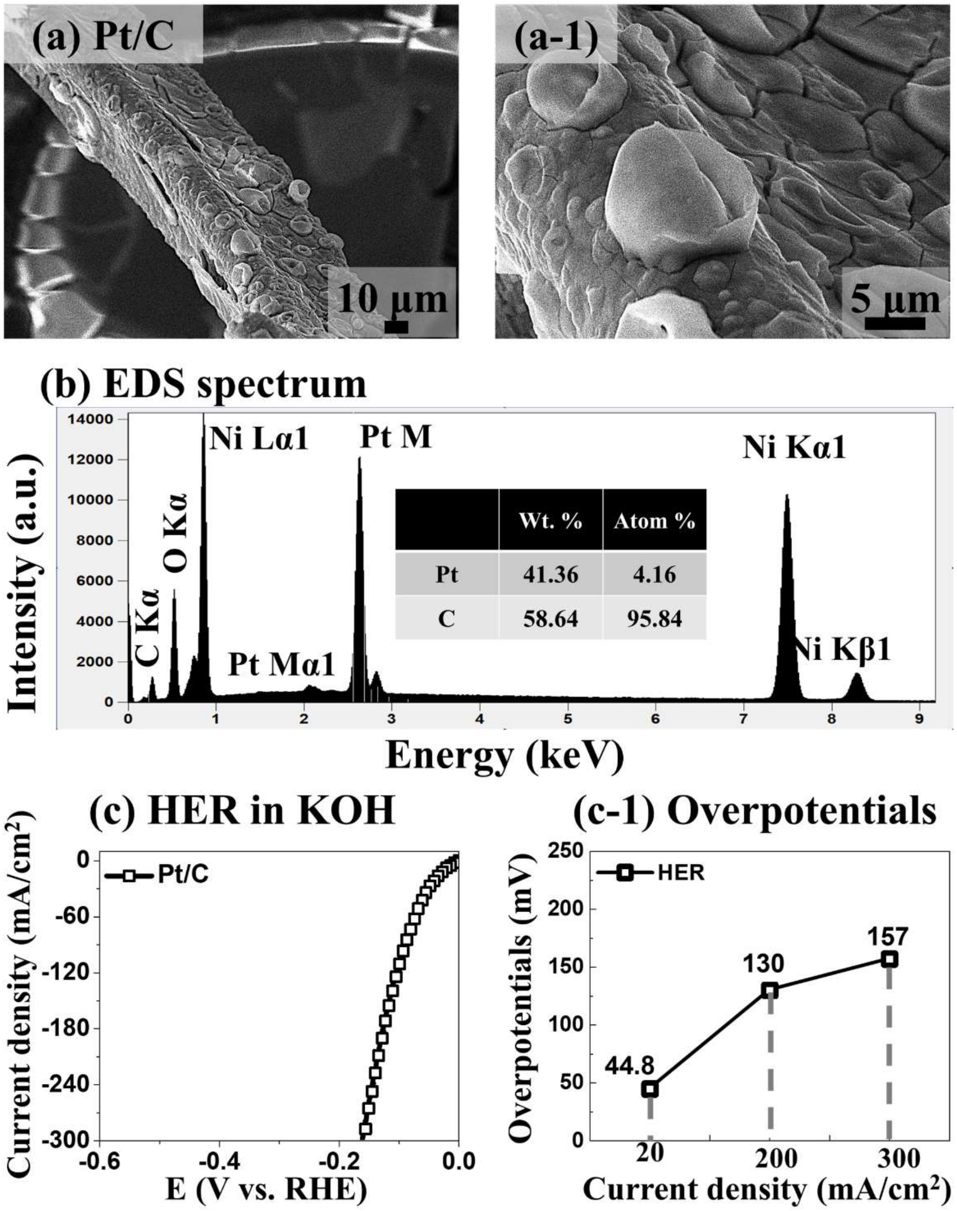

Figure S4: (a) – (a-1) SEM images of Pt/C reference electrode. (b) EDS spectrum. (c) – (c-1) HER activity of Pt/C electrode and corresponding overpotential values at different current densities. More details can be found in the supplementary text S1.4.

S2.1.5. RuO_2 (OER) reference electrode

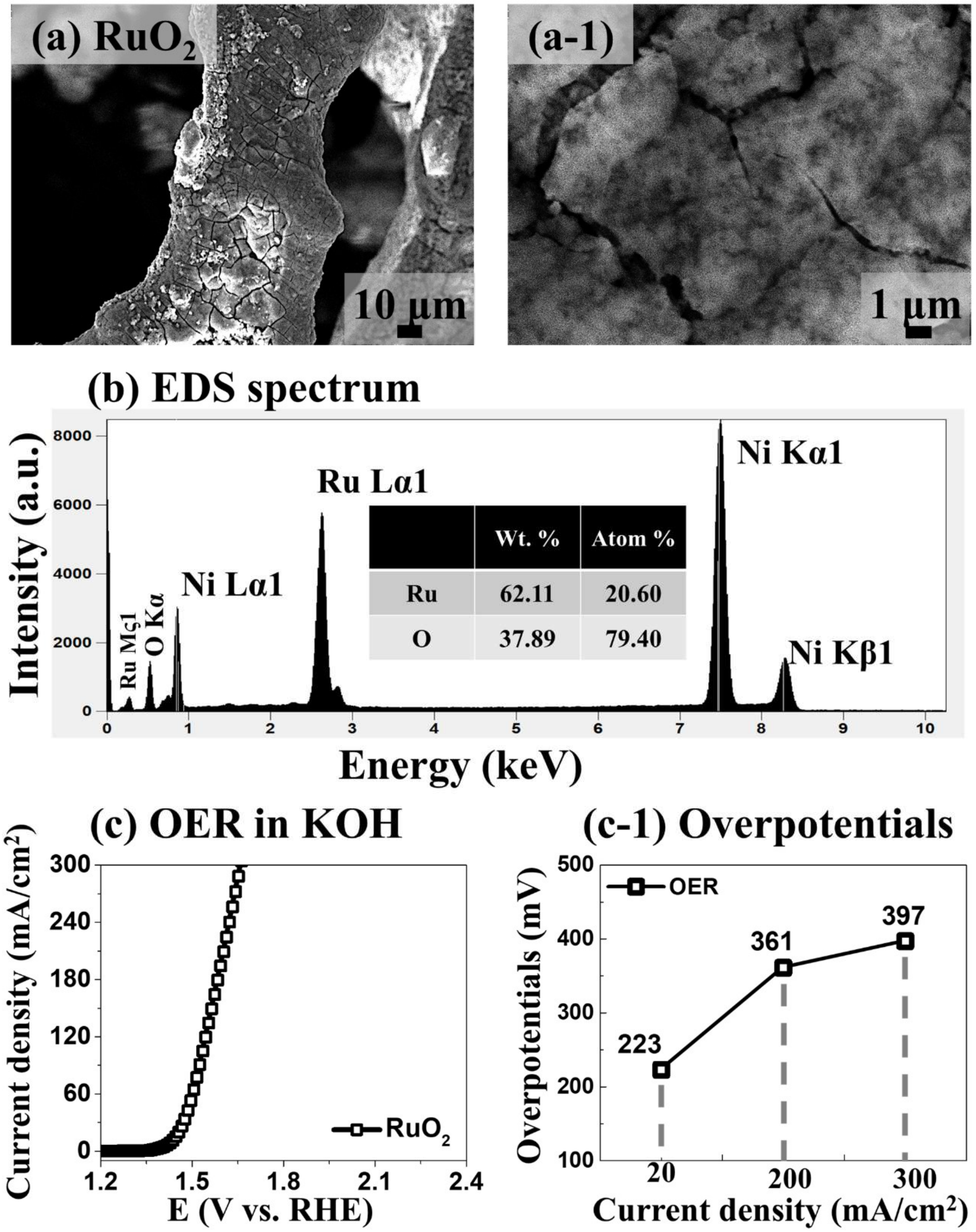

Figure S5: (a) – (a-1) SEM images of RuO_2 reference electrode. (b) EDS spectrum. (c) – (c-1) OER activity of RuO_2 electrode and corresponding overpotential values at different current densities. More details can be found in the supplementary text S1.4.

S2. CoFeBP MFB electrocatalysts

S2.1. Substrate and other necessary characterizations

S2.1.1. Bare Ni foam characterization

S2.1.2. LSV at different scan rates with the best CoFeBP

S2.1.3. EIS voltage variation of the best CoFeBP MFBs

S2.1.4. Pt/C (HER) reference electrode

S2.1.5. RuO_2 (OER) reference electrode

S2.2. Reaction parameter and precursor optimizations

S2.2.1. Fabrication steps of CoFeBP MFB

S2.2.2. Reaction duration optimization

S2.2.3. Reaction temperature optimization

S2.2.4. B-P concentration optimization

S2.2.5. Co-Fe concentration optimization

S2.2.6. Urea concentration optimization

S2.2.7. Urea- Ammonium fluoride concentration optimization

S2.3. Post-annealing optimization

S2.3.1. Post-annealing time optimization

S2.3.2. Post-annealing temperature optimization

S2.4. Before and after post-annealing analysis of CoFeBP MFB

S2.4.1. Before and after post-annealing: Raman

S2.4.2. Before and after post-annealing: XRD

S2.4.3. Before and after post-annealing: EIS

S2.4.4. Before and after post-annealing: HER/OER LSV

S2.1.1. Fabrication steps of CoFeBP MFB electrode

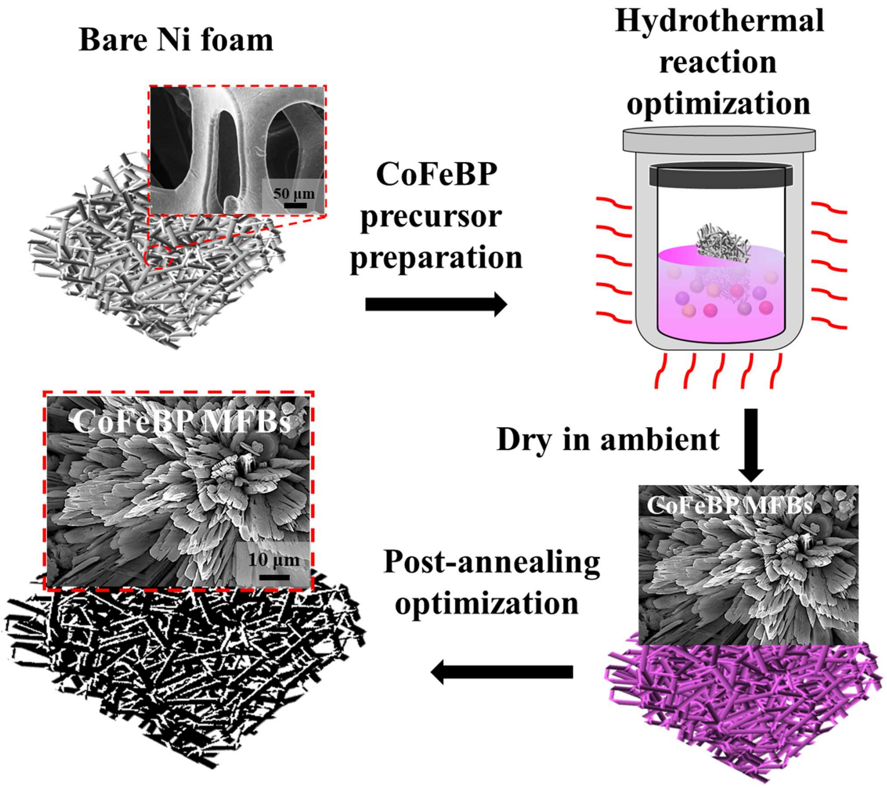

Figure S6: Fabrication setps of CoFeBP micro flower branch (MFB) electrode on the porous nickel foam (NF) via the hydrothermal reaction. The best sample from the hydrothermal reaction was treated with post-annealing. More details on the fabrication of CoFeBP MFB can be found in the supplementary text S1.5.

S2.2.2. Hydrothermal reaction duration optimization: morpholgy

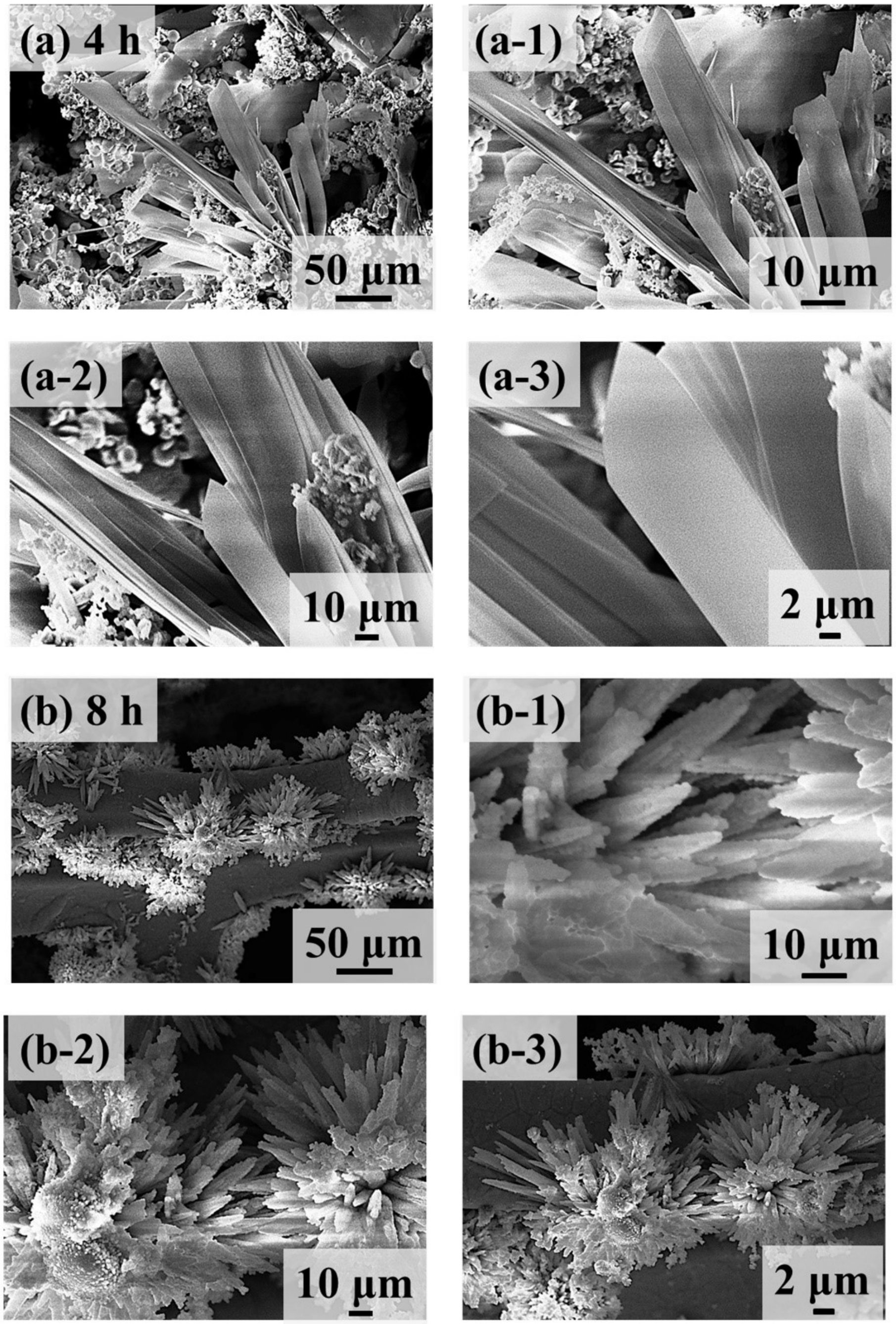

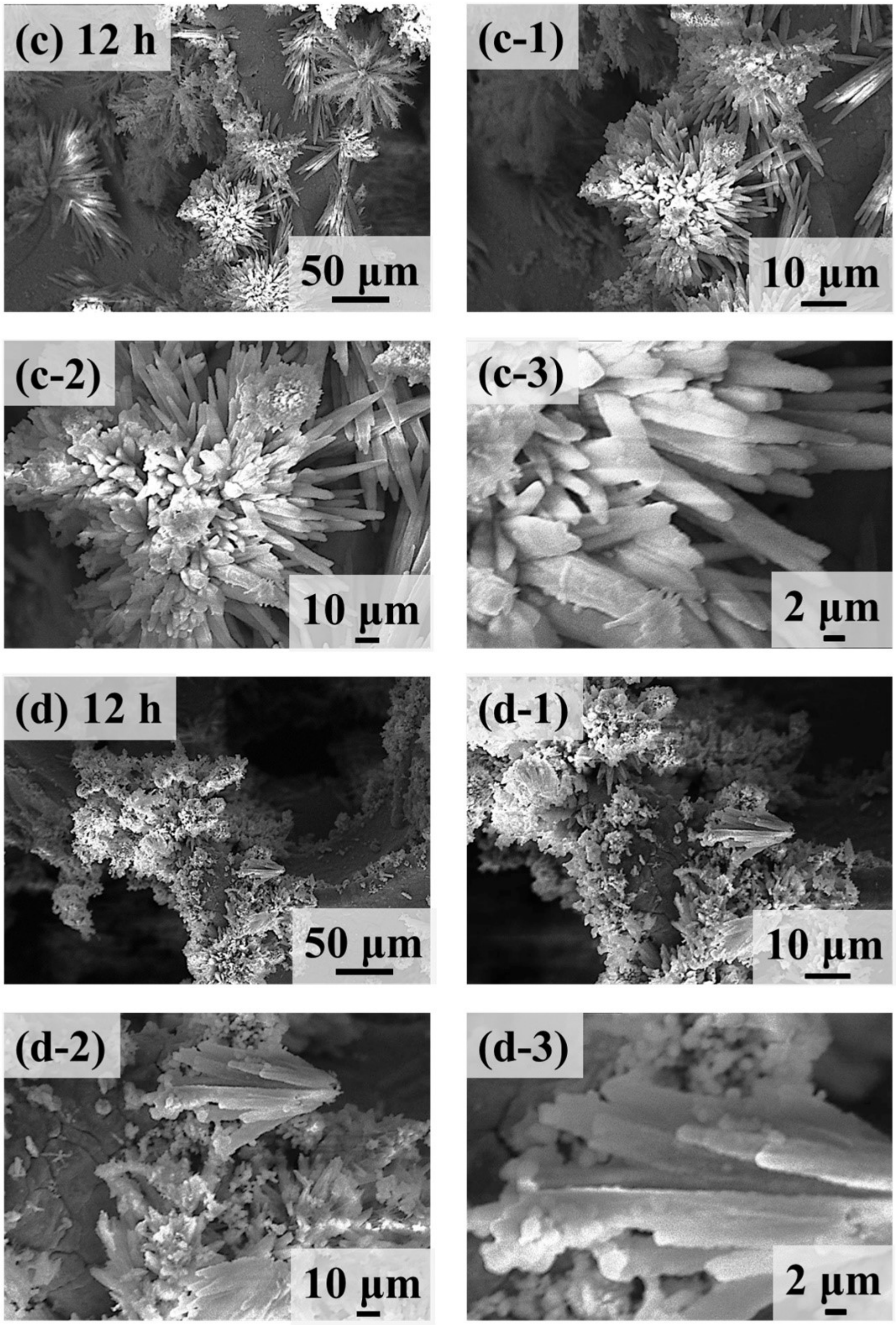

Figure S7: (a) – (d) SEM images of CoFeBP electrodes at different reaction duration . (a-1) – (a-3), (b-1) – (b-3), (c-1) – (c-3), and (d-1) – (d-3) Corresponding zoom-in images.

S2.2.2. Hydrothermal reaction duration optimization: LSV

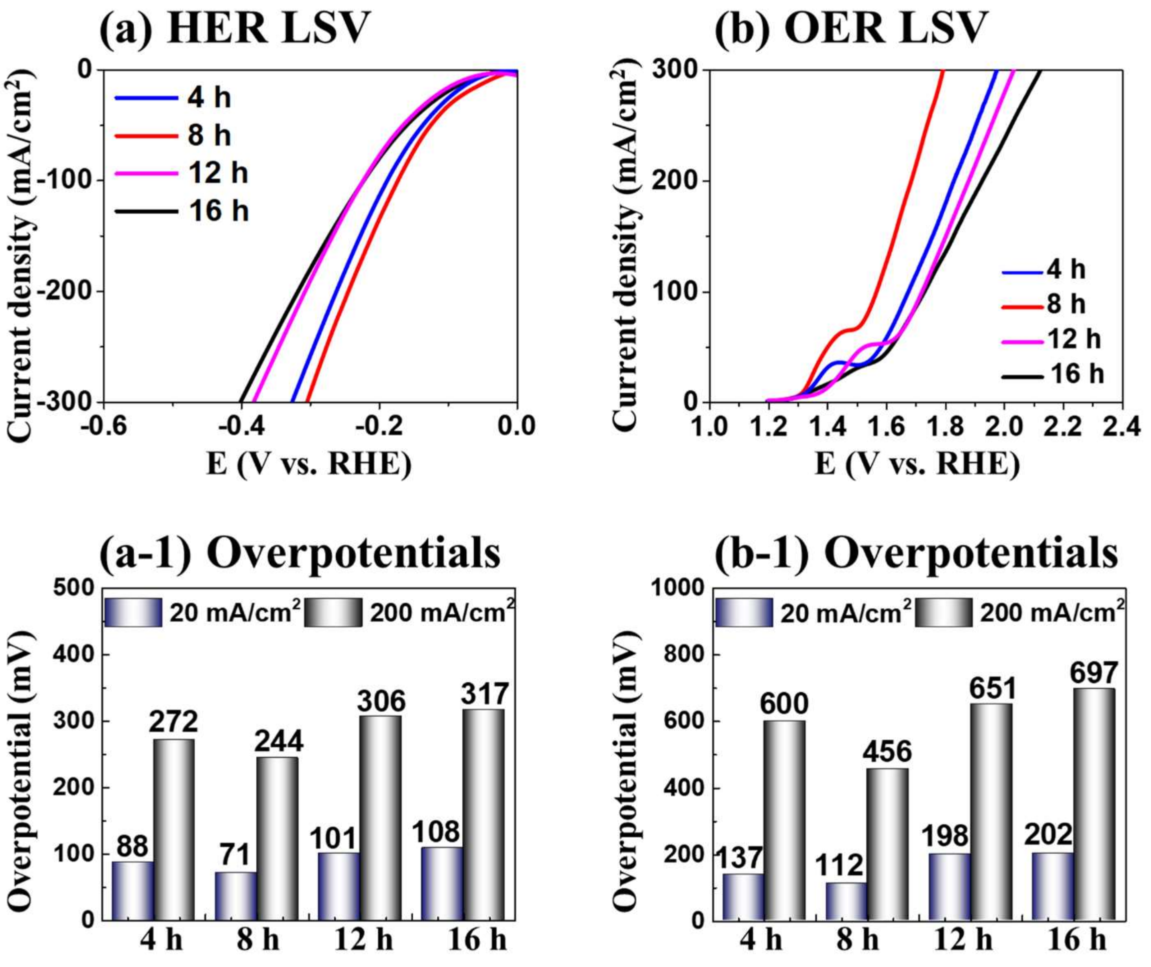

(a-2)

Best electrode	Overpotential @200 mA/cm²
8 h	244 mV

(b-2)

Best electrode	Overpotential @200 mA/cm²
8 h	456 mV

Figure S8: HER/OER performances of CoFeBP electrodes with hydrothermal deposition time variation. (a) & (b) HER/OER LSV results. (a-1) & (b-1) Corresponding overpotential values at 200 mA/cm^2. (a-2) & (b-2) HER/OER overpotential values of the best electrode at 200 mA/cm^2.

S2.2.3. Hydrothermal reaction temperature optimization: morpholgy

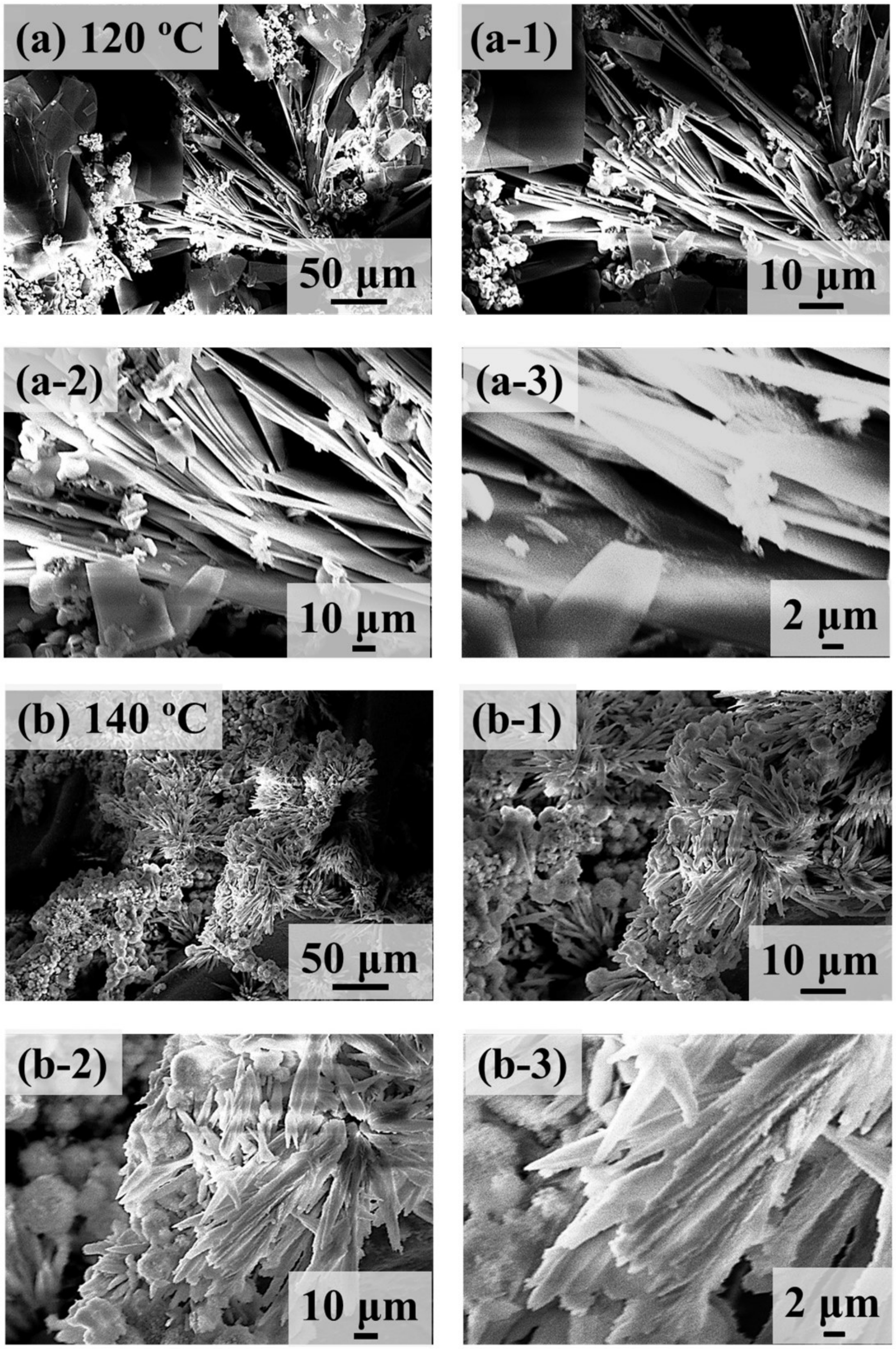

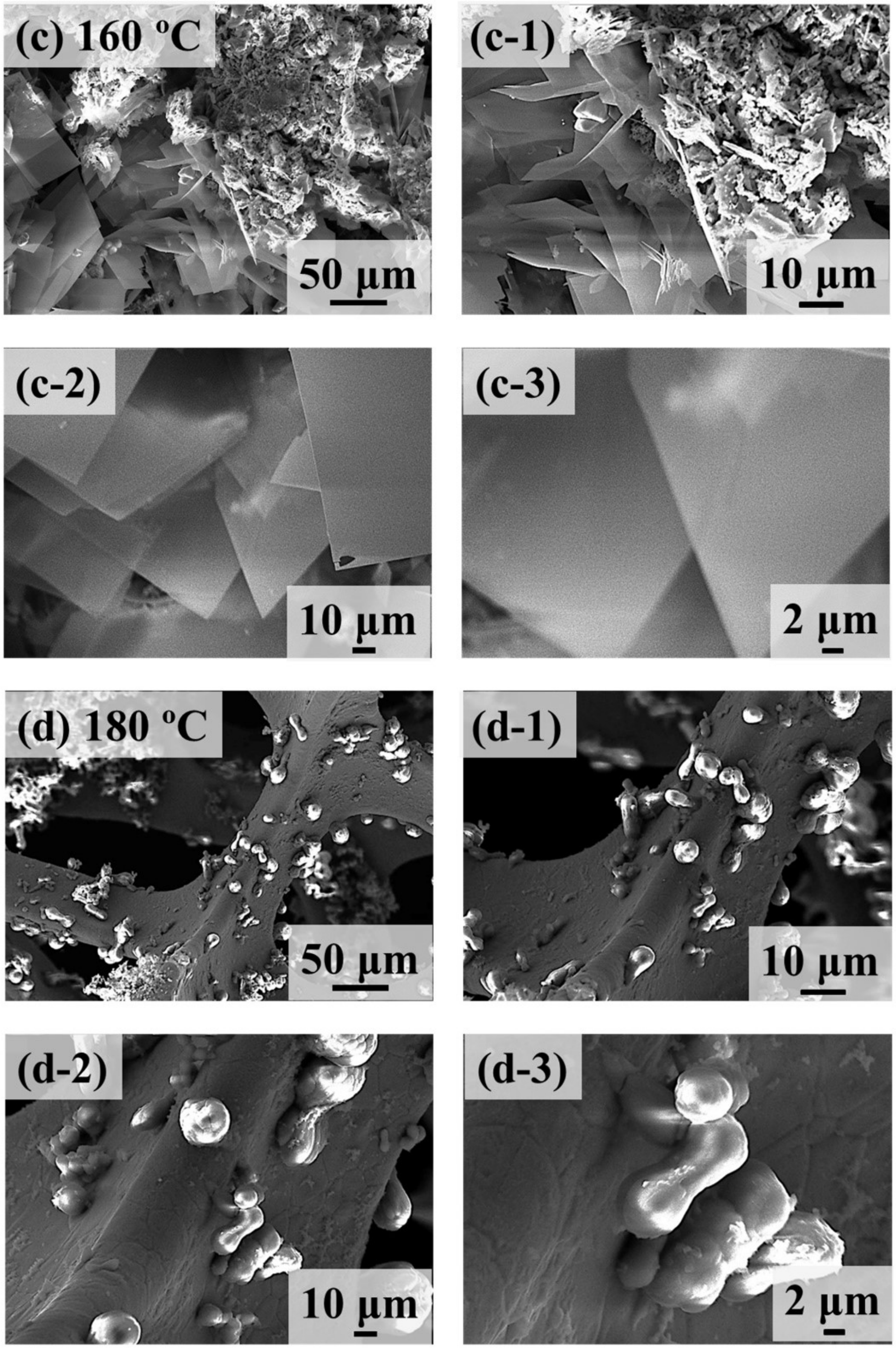

Figure S9: (a) – (d) SEM images of CoFeBP electrodes with hydrothermal reaction temperature variation. (a-1) – (a-3), (b-1) – (b-3), (c-1) – (c-3), and (d-1) – (d-3) Corresponding zoom-in images.

S2.2.3. Hydrothermal reaction temperature optimization: LSV

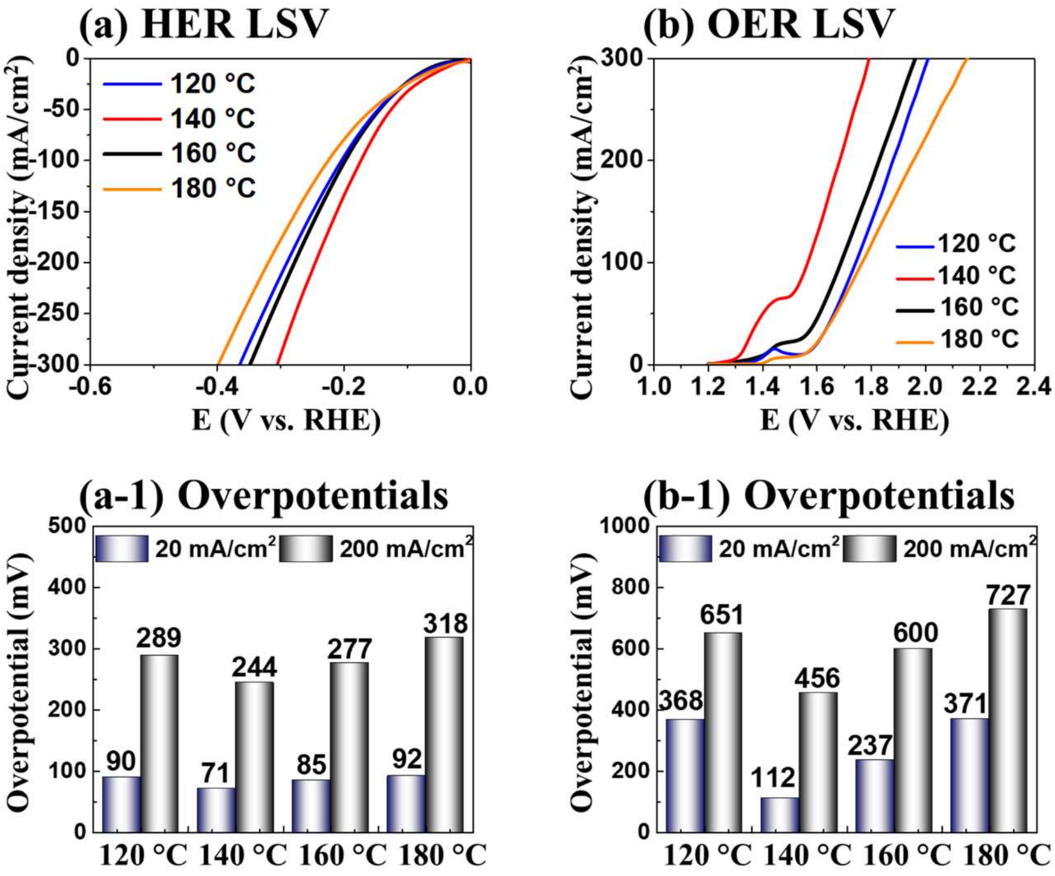

(a-2)

Best electrode	Overpotential @200 mA/cm^2
140 °C	244 mV

(b-2)

Best electrode	Overpotential @200 mA/cm^2
140 °C	456 mV

Figure S10: HER/OER performances of CoFeBP electrodes with hydrothermal reaction temperature variation**.** The duration of hydrothermal process was fixed for 8 h. (a) & (b) HER/OER LSV results. (a-1) & (b-1) Corresponding overpotential values at 200 mA/cm^2. (a-2) & (b-2) HER/OER overpotential values of the best electrode at 200 mA/cm^2.

S2.2.4. B-P concentration optimization: morpholgy

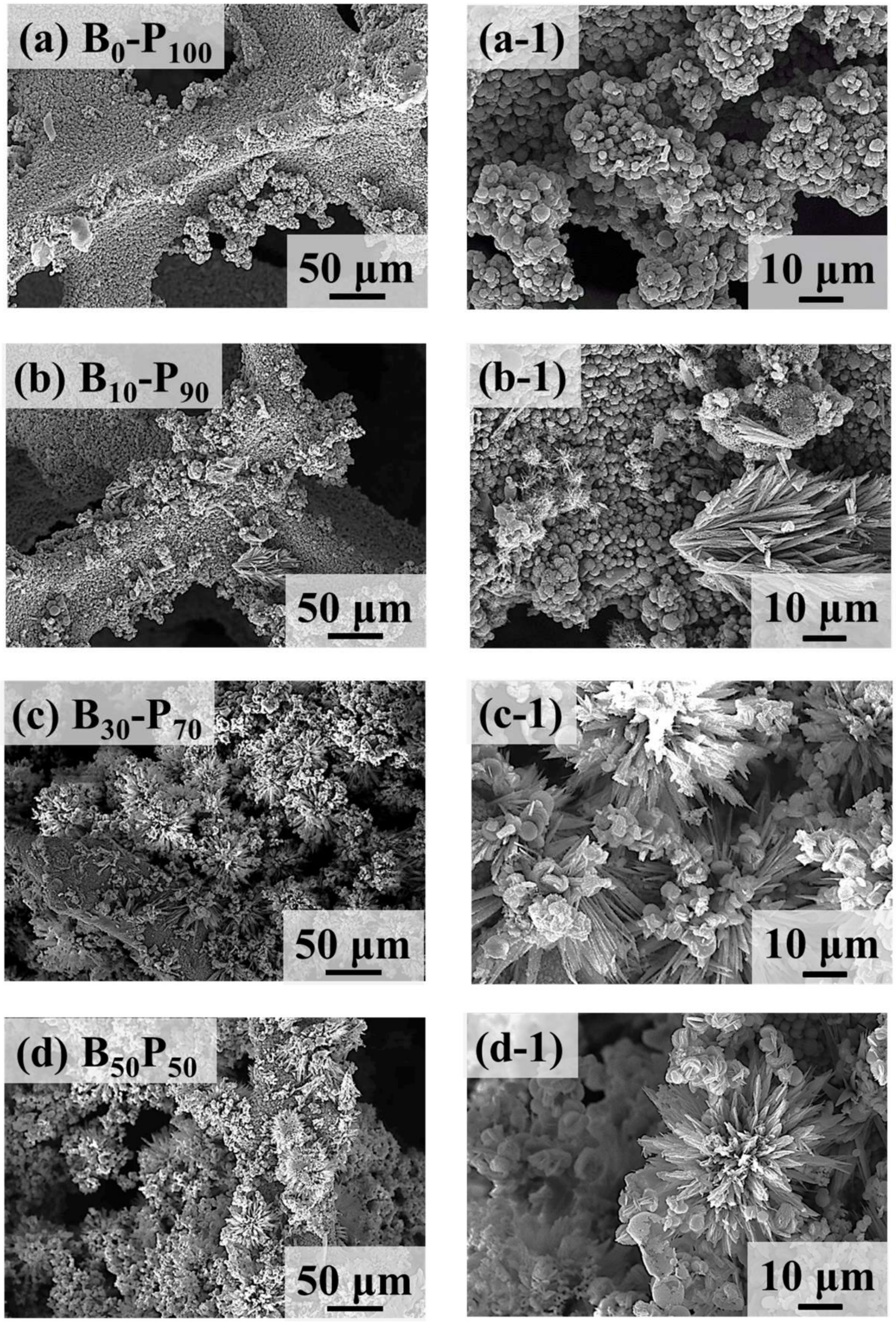

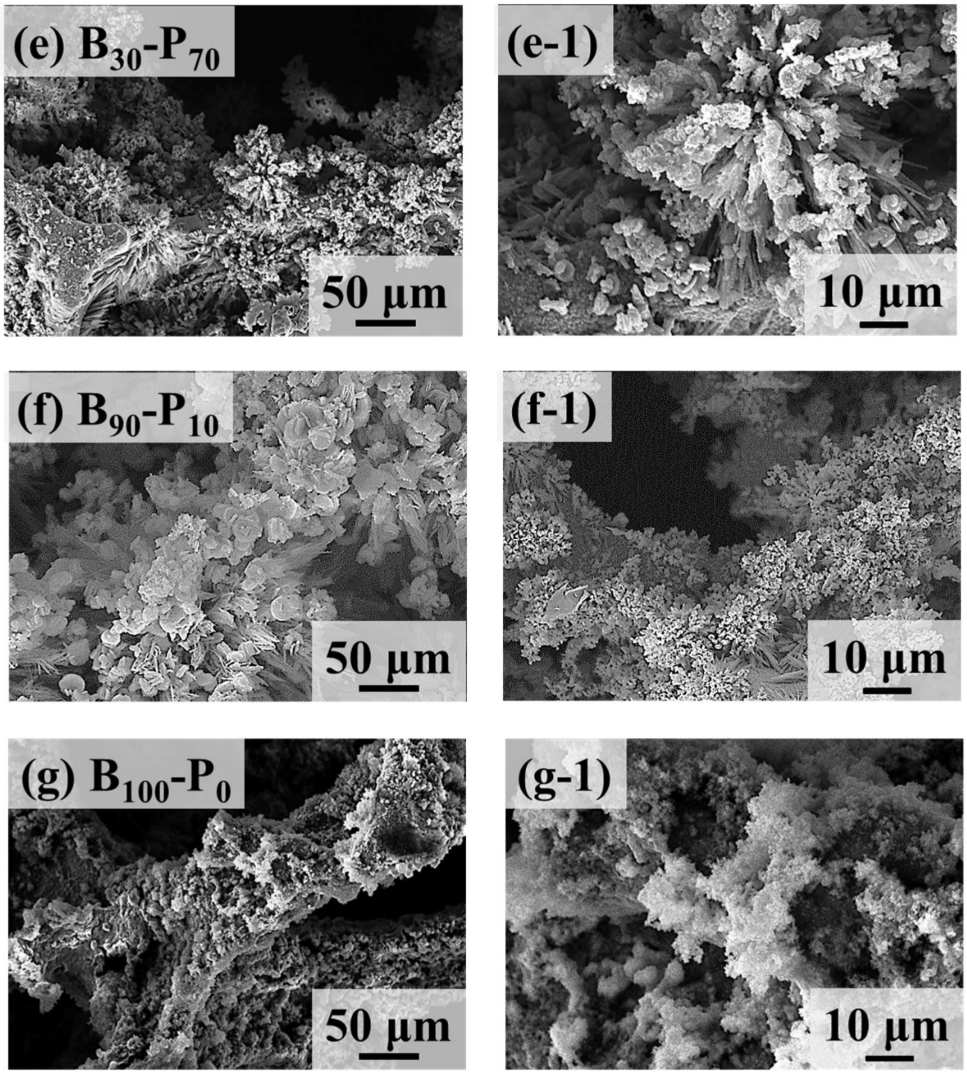

Figure S11: (a) – (g) SEM images of CoFeBP MFB electrodes with the B-P concentration variation. (a-1) – (g-1) Corresponding zoom-in images.

S2.2.4. B-P concentration optimization: EDS

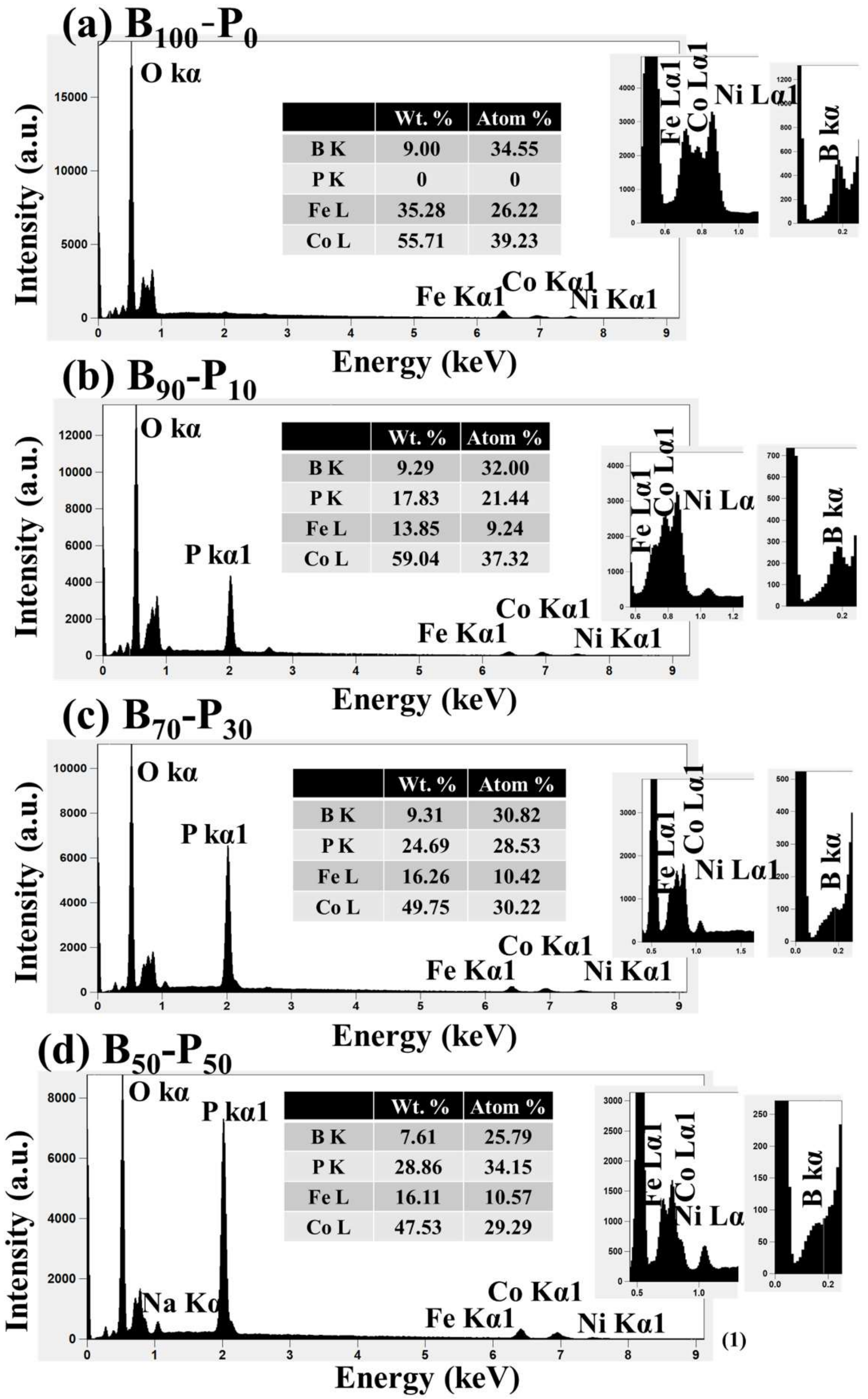

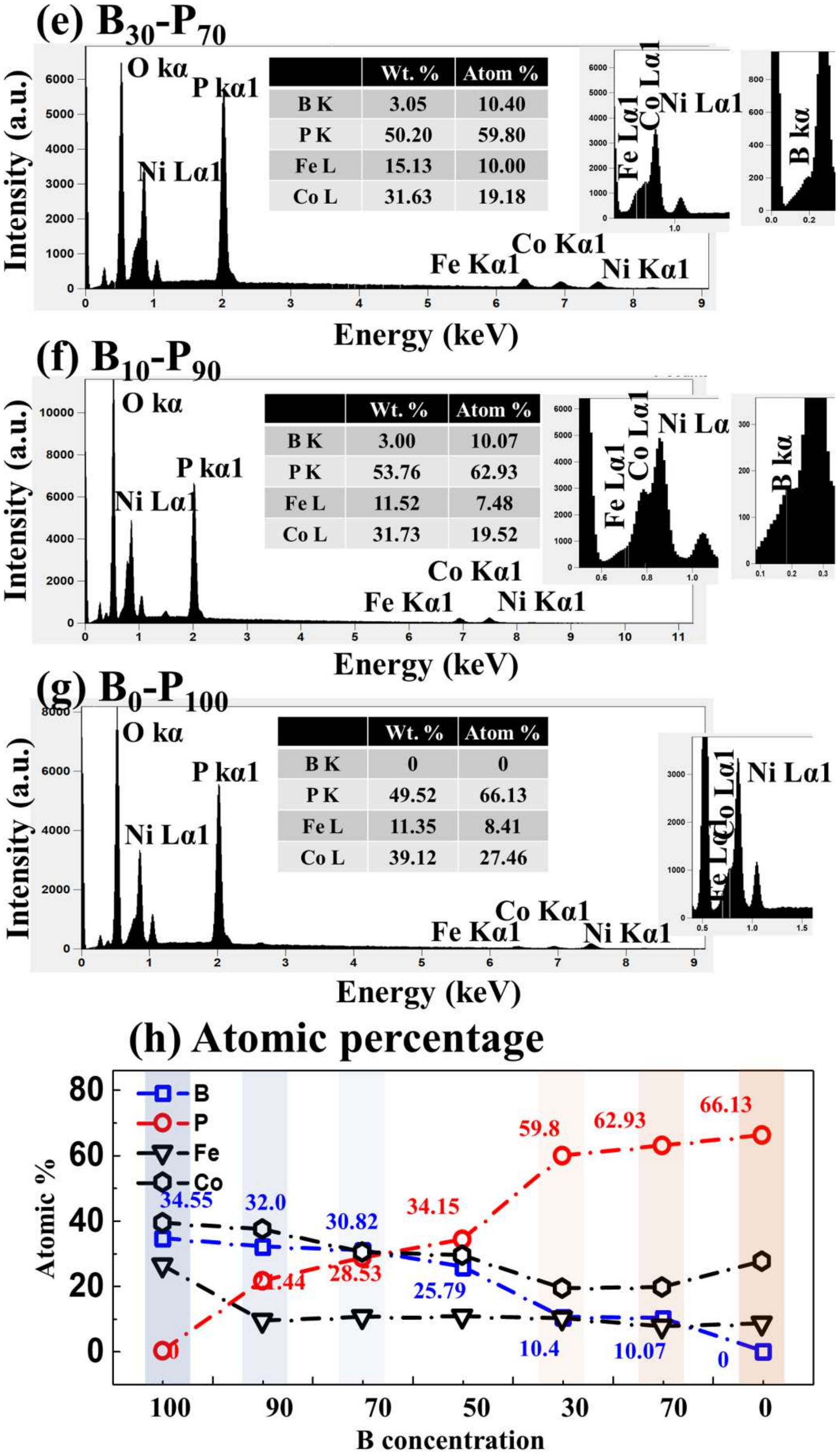

	Wt. %	Atom %
B K	3.05	10.40
P K	50.20	59.80
Fe L	15.13	10.00
Co L	31.63	19.18

	Wt. %	Atom %
B K	3.00	10.07
P K	53.76	62.93
Fe L	11.52	7.48
Co L	31.73	19.52

	Wt. %	Atom %
B K	0	0
P K	49.52	66.13
Fe L	11.35	8.41
Co L	39.12	27.46

Figure S12: (a) – (g) EDS spectra of CoFeBP electrodes in the B-P concentration variation. (h) Summary plot of atomic percentage.

S2.2.4. B-P concentration optimization: LSV

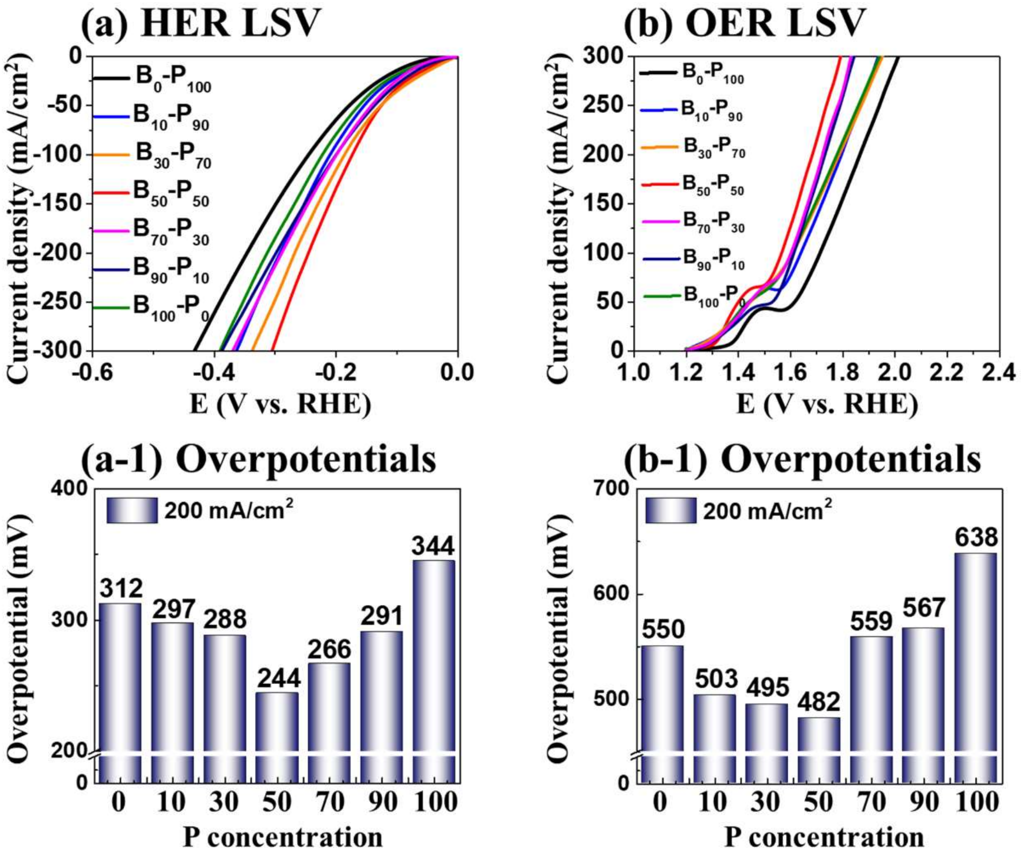

(a-2)

Best electrode	Overpotential @200 mA/cm^2
B50-P50	244 mV

(b-2)

Best electrode	Overpotential @200 mA/cm^2
B50-P50	482 mV

Figure S13: (a) & (b) HER/OER LSV summary of B-P concentration variation set. (a-1) & (b-1) Corresponding overpotential values at 200 mA/cm^2. (a-2) & (b-2) HER/OER overpotential values of the best electrode at 200 mA/cm^2.

S2.2.5. Co-Fe concentration optimization: morpholgy

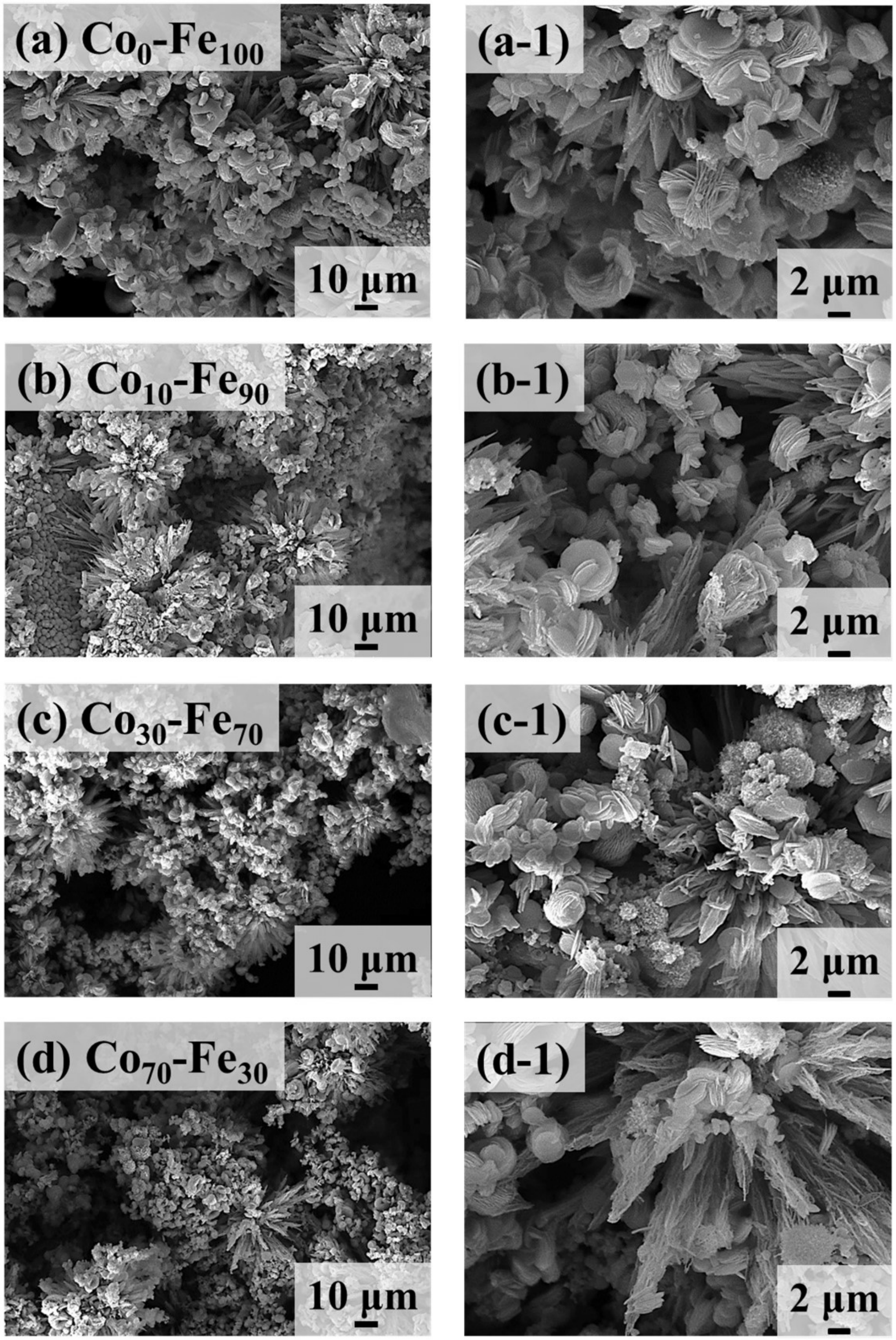

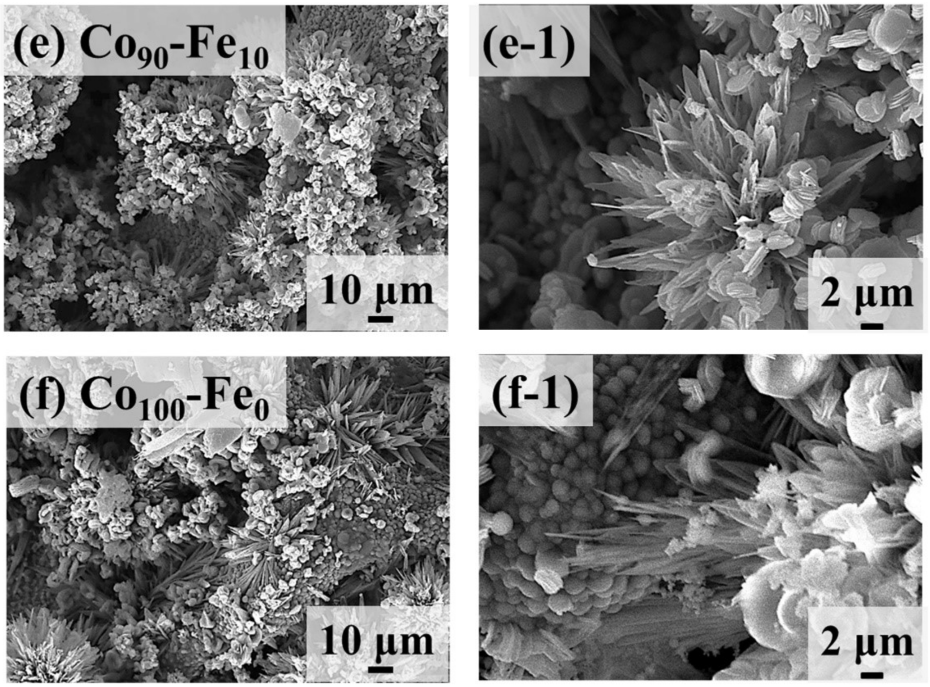

Figure S14: (a) – (f) SEM images of CoFeBP MFB electrodes with the Co-Fe concentration variation. (a-1) – (f-1) Corresponding zoom-in images.

S2.2.5. Co-Fe concentration optimization: EDS

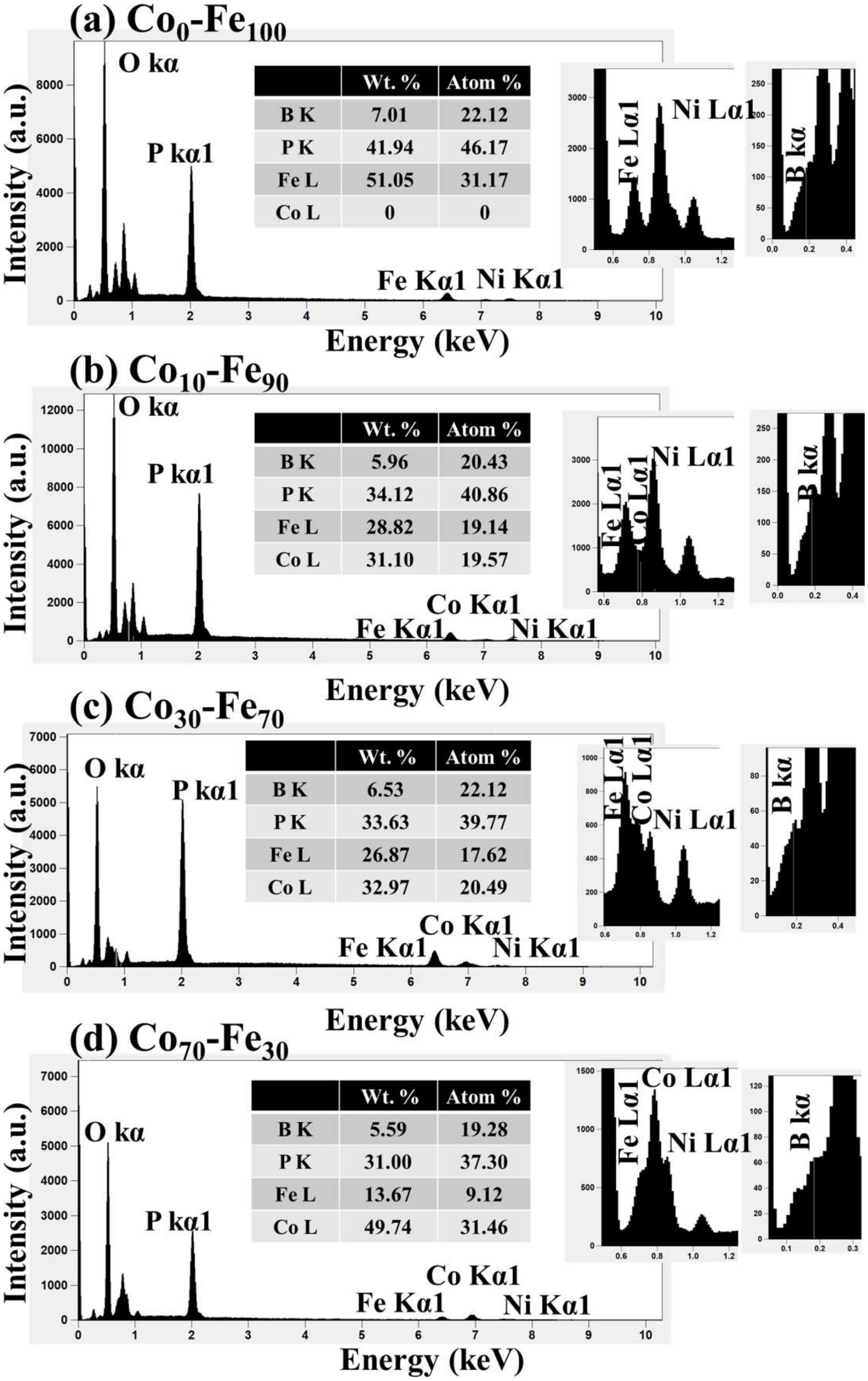

(a) Co_0-Fe_{100}

	Wt. %	Atom %
B K	7.01	22.12
P K	41.94	46.17
Fe L	51.05	31.17
Co L	0	0

(b) Co_{10}-Fe_{90}

	Wt. %	Atom %
B K	5.96	20.43
P K	34.12	40.86
Fe L	28.82	19.14
Co L	31.10	19.57

(c) Co_{30}-Fe_{70}

	Wt. %	Atom %
B K	6.53	22.12
P K	33.63	39.77
Fe L	26.87	17.62
Co L	32.97	20.49

(d) Co_{70}-Fe_{30}

	Wt. %	Atom %
B K	5.59	19.28
P K	31.00	37.30
Fe L	13.67	9.12
Co L	49.74	31.46

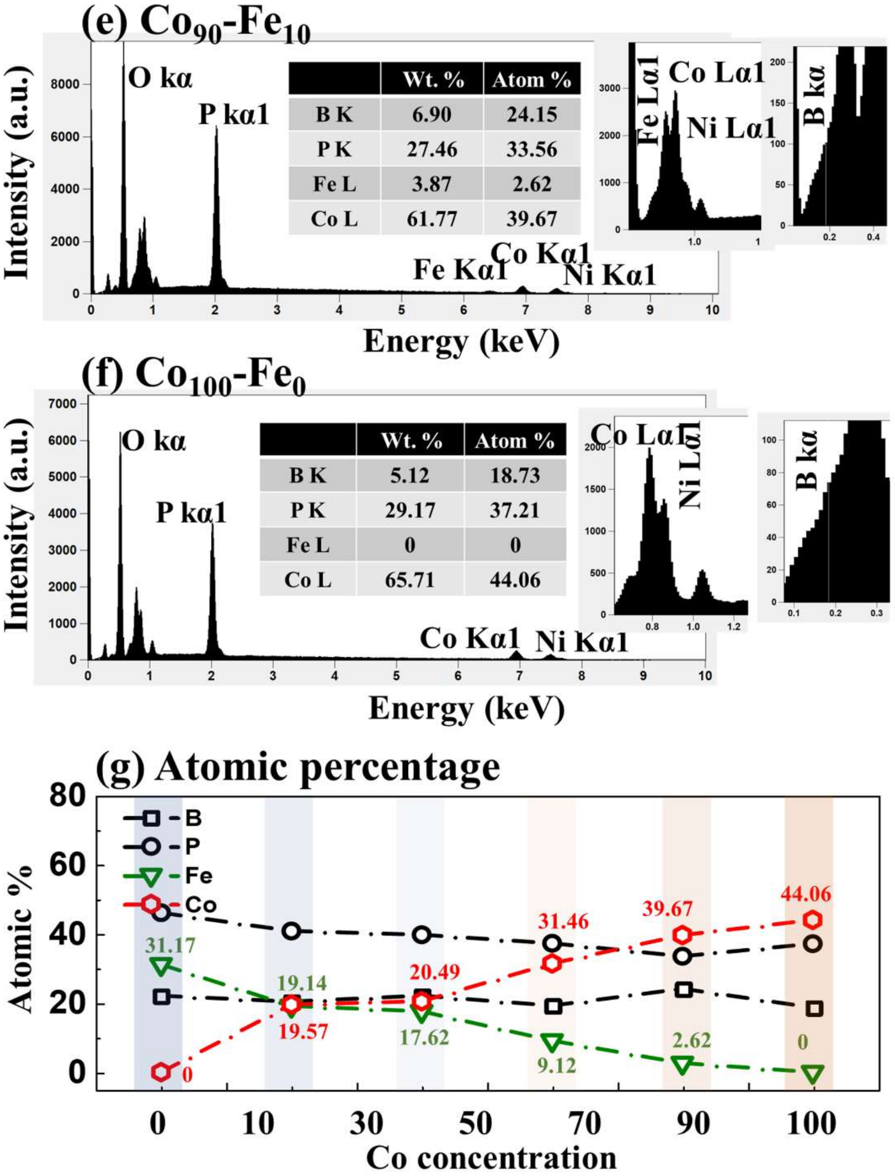

Figure S15: (a) – (f) EDS spectra of CoFeBP electrodes in the Co-Fe concentration variation. (g) Atomic percentage summary plot.

S2.2.5. Co-Fe concentration optimization: LSV

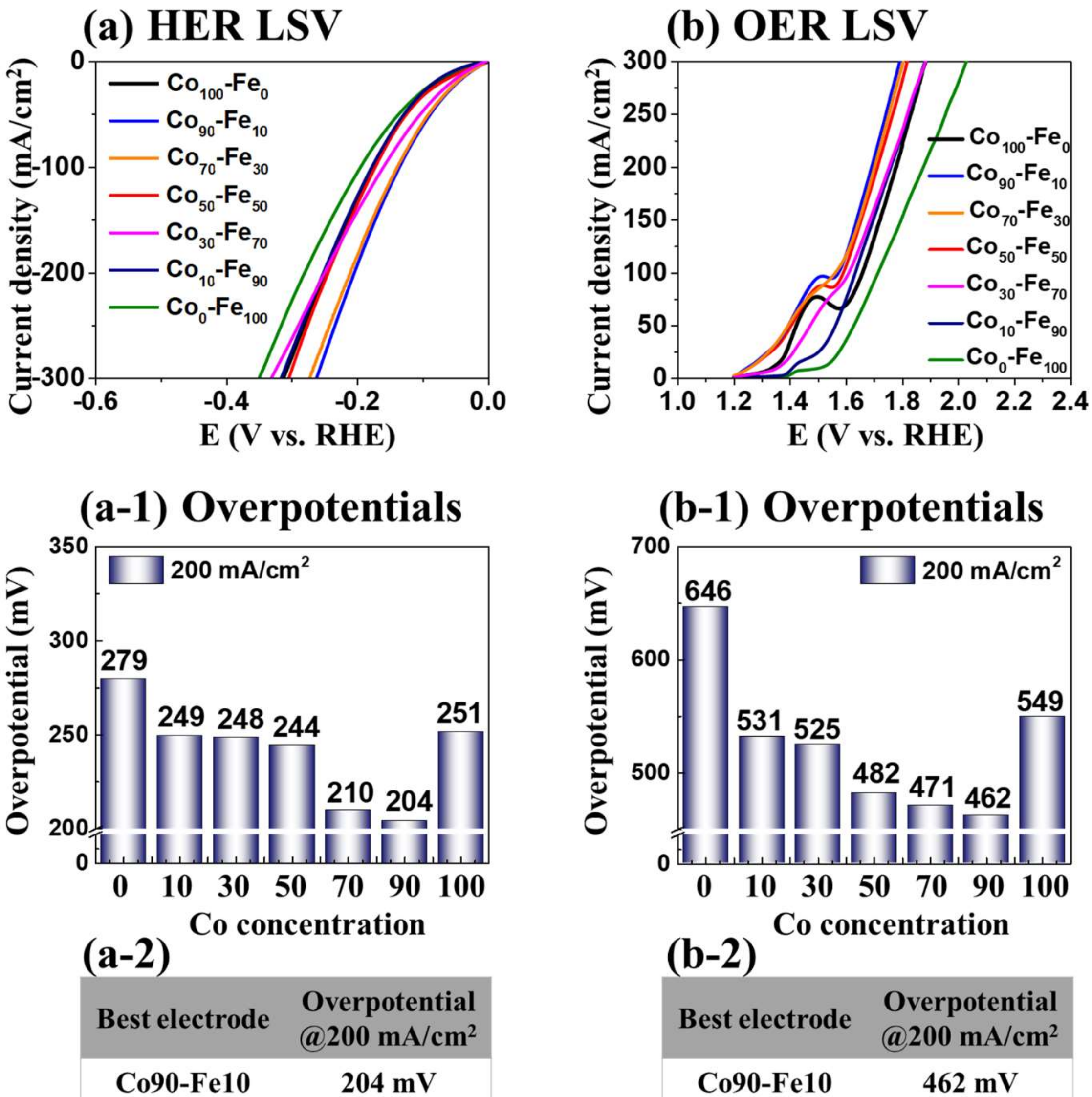

(a-2)

Best electrode	Overpotential @200 mA/cm²
Co90-Fe10	204 mV

(b-2)

Best electrode	Overpotential @200 mA/cm²
Co90-Fe10	462 mV

Figure S16: (a) & (b) HER/OER LSV results. (a-1) & (b-1) Corresponding overpotential values at 200 mA/cm^2. (a-2) & (b-2) HER/OER overpotential values of the best electrode at 200 mA/cm^2.

S2.2.6. Urea concentration optimization: morpholgy

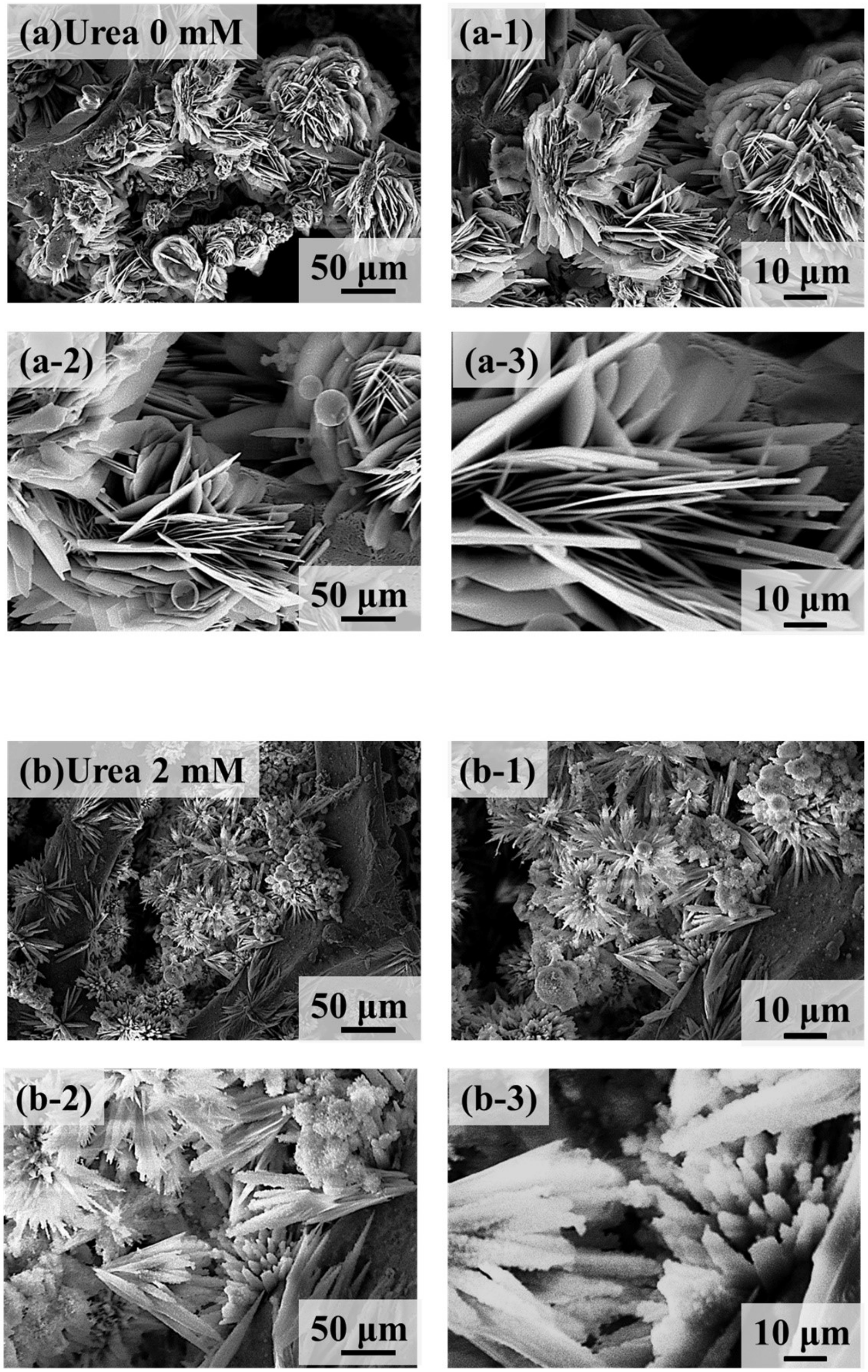

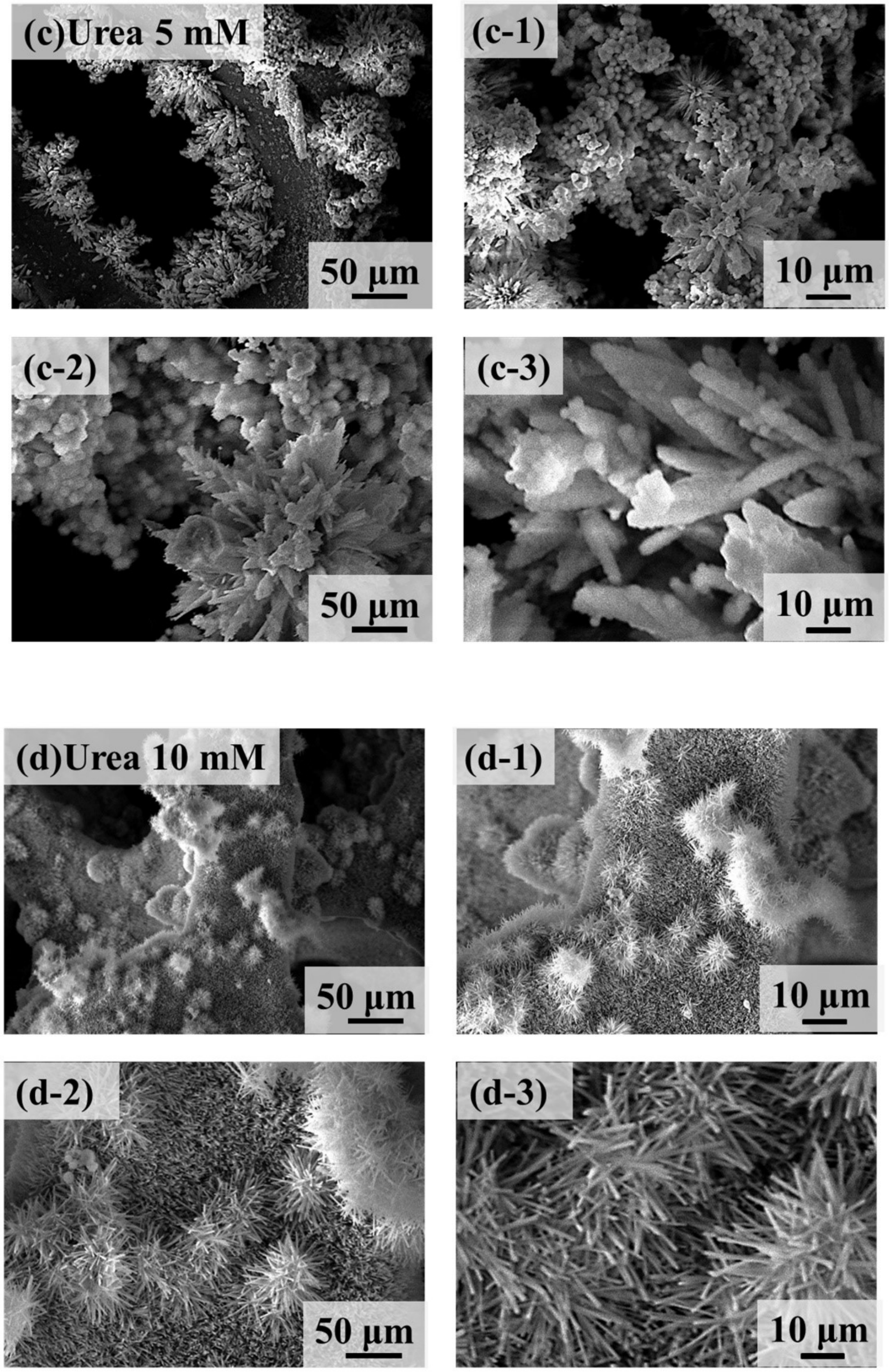

Figure S17: (a) – (d) SEM images of CoFeBP electrodes at different urea concentration. (a-1) – (a-3), (b-1) – (b-3), (c-1) – (c-3), and (d-1) – (d-3) Corresponding zoom-in images. Different urea concentration resulted in altered morphologies of CoFeBP.

S2.2.6. Urea concentration optimization: LSV

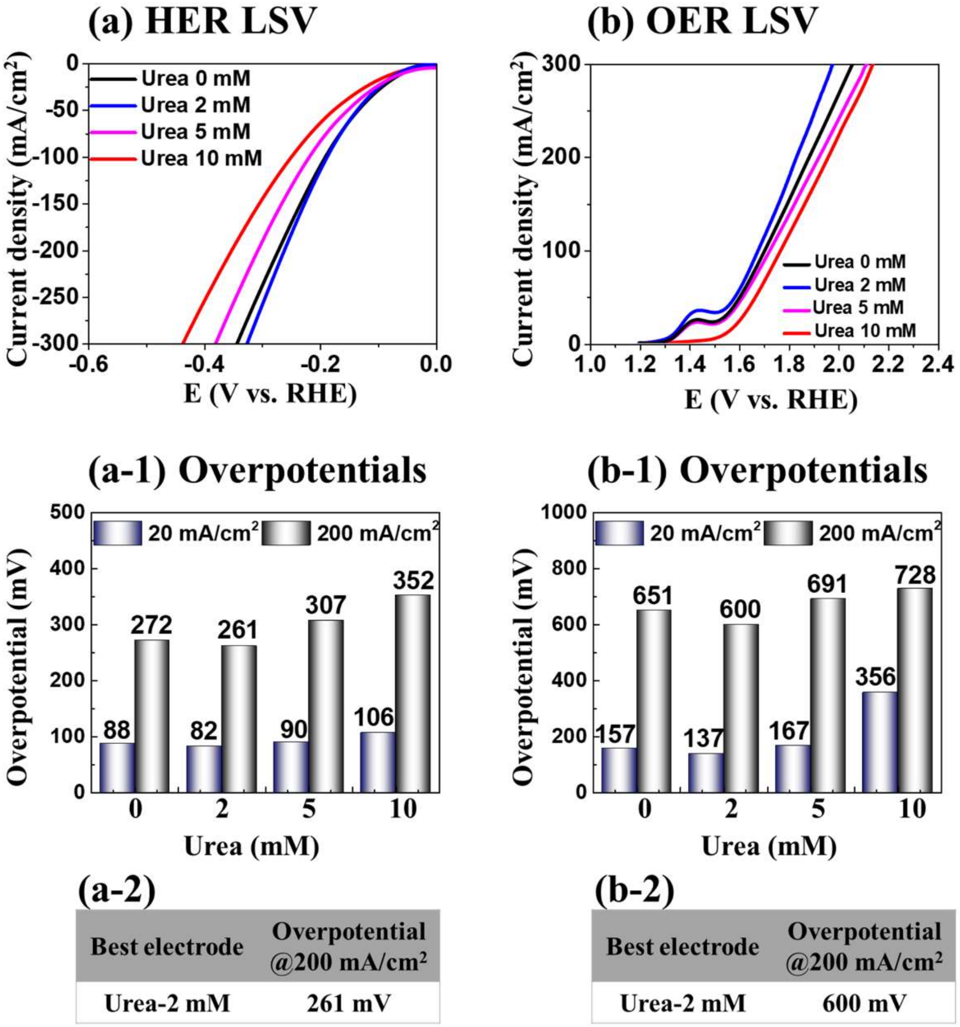

Figure S18: HER/OER performances of CoFeBP electrodes with urea concentration variation. Hydrothermal reaction parameter was fixed at 4 h at 140 °C. (a) & (b) HER/OER LSV results. (a-1) & (b-1) Corresponding overpotential values at 200 mA/cm^2. (a-2) & (b-2) HER/OER overpotential values of the best electrode at 200 mA/cm^2.

S2.2.7. Urea-Ammonium fluoride optimization: morpholgy

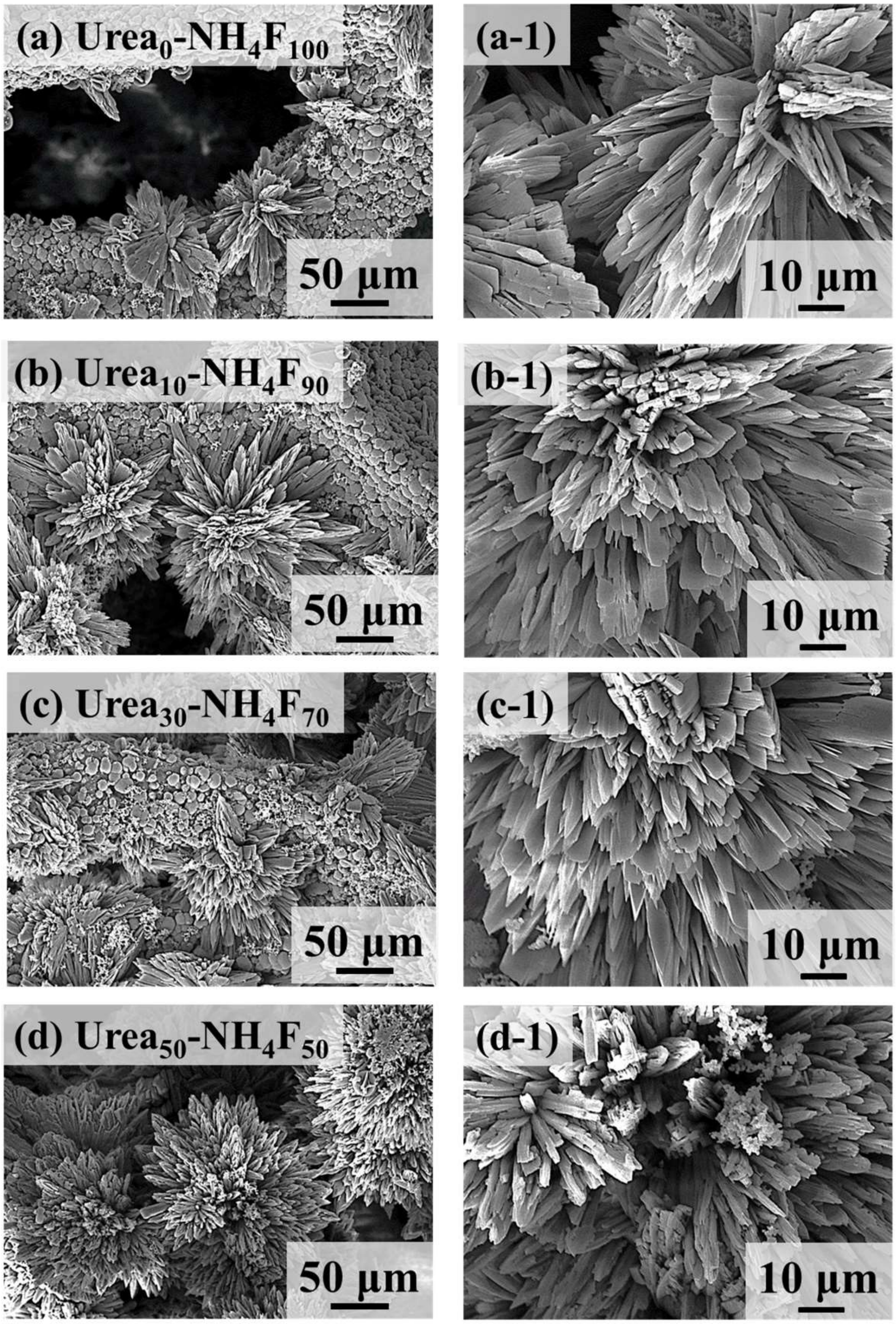

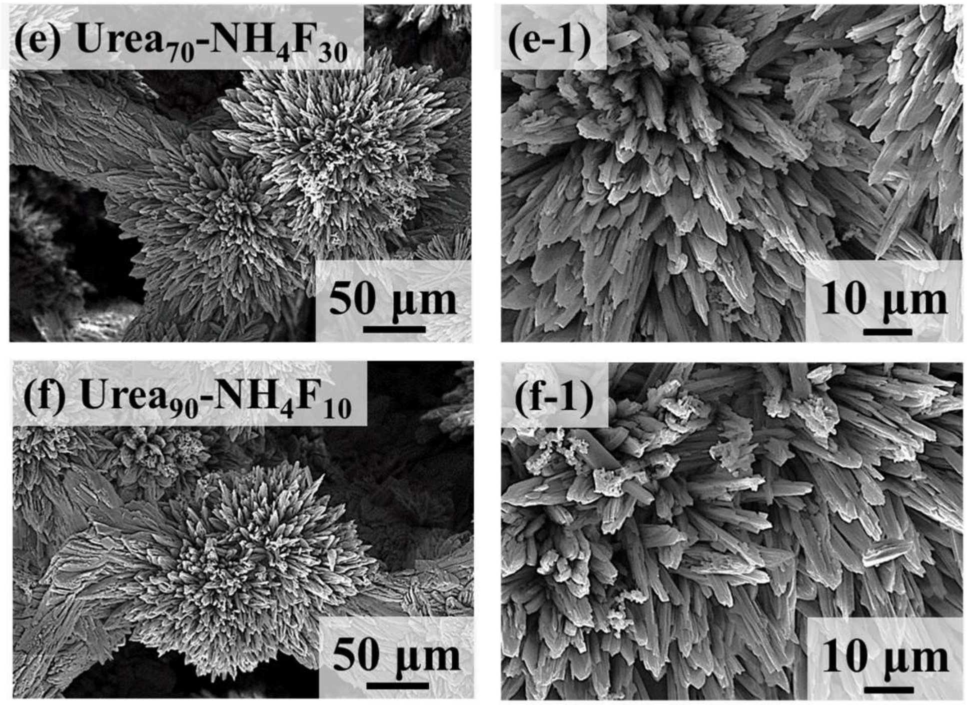

Figure S19: (a) – (f) SEM images of Urea-Ammonium fluoride concentration variation. (a-1) – (f-1) Corresponding zoom-in images.

S2.2.7. Urea-Ammonium fluoride optimization: LSV

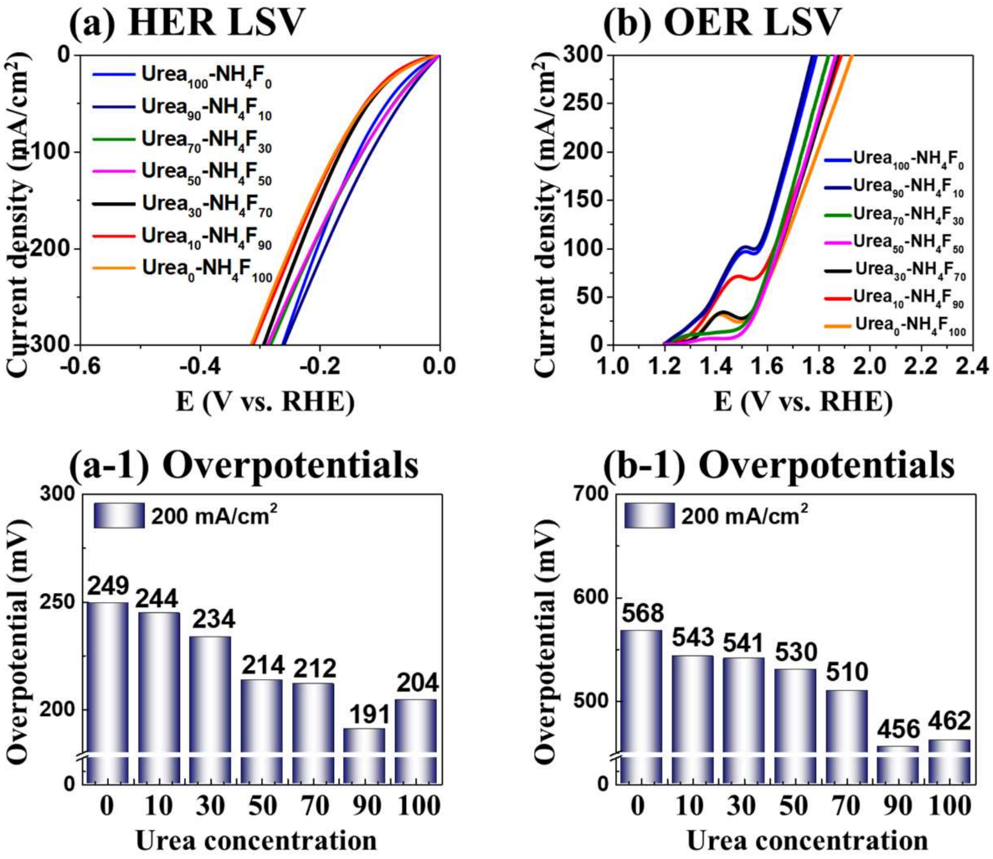

(a-2)

Best electrode	Overpotential @200 mA/cm^2
Urea-90	191 mV

(b-2)

Best electrode	Overpotential @200 mA/cm^2
Urea-90	456 mV

Figure S20: Electrochemical performance of CoFeBP electrodes with Urea-Ammonium fluoride concentration variation set. (a) & (b) HER/OER LSV results. (a-1) & (b-1) Corresponding overpotential values at 200 mA/cm^2. (a-2) & (b-2) HER/OER overpotential values of the best electrode at 200 mA/cm^2.

S2. CoFeBP MFB electrocatalysts

S2.1. Substrate and other necessary characterizations

S2.1.1. Bare Ni foam characterization

S2.1.2. LSV at different scan rates with the best CoFeBP

S2.1.3. EIS voltage variation of the best CoFeBP MFBs

S2.1.4. Pt/C (HER) reference electrode

S2.1.5. RuO_2 (OER) reference electrode

S2.2. Reaction parameter and precursor optimizations

S2.2.1. Fabrication steps of CoFeBP MFB

S2.2.2. Reaction duration optimization

S2.2.3. Reaction temperature optimization

S2.2.4. B-P concentration optimization

S2.2.5. Co-Fe concentration optimization

S2.2.6. Urea concentration optimization

S2.2.7. Urea- Ammonium fluoride concentration optimization

S2.3. Post-annealing optimization

S2.3.1. Post-annealing time optimization

S2.3.2. Post-annealing temperature optimization

S2.4. Before and after post-annealing analysis of CoFeBP MFB

S2.4.1. Before and after post-annealing: Raman

S2.4.2. Before and after post-annealing: XRD

S2.4.3. Before and after post-annealing: EIS

S2.4.4. Before and after post-annealing: HER/OER LSV

S2.3.1. Post-annealing duration optimization: morpholgy

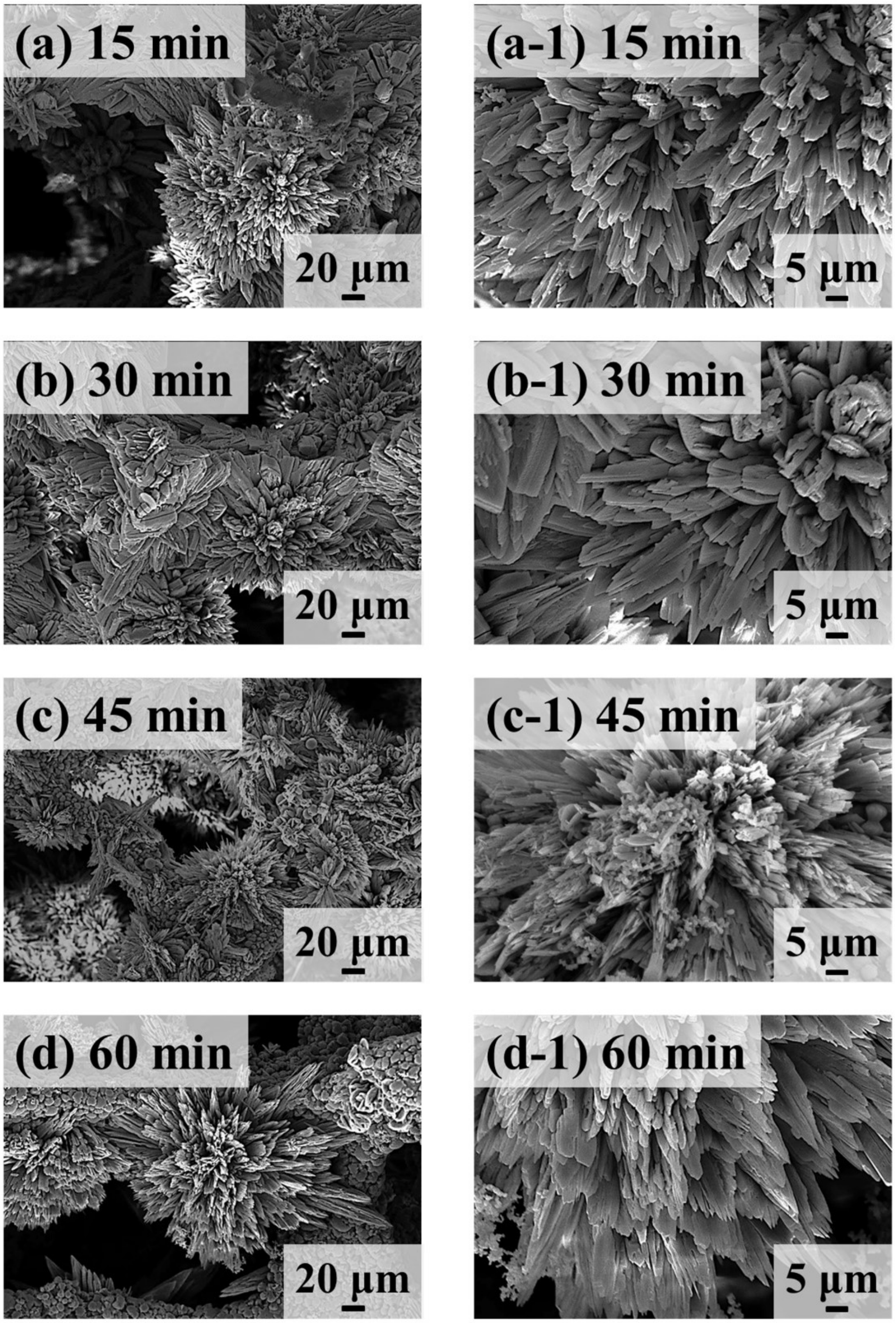

Figure S21: (a) – (d) SEM images of CoFeBP electrodes with post-annealing duration variation. Annealing temperature was fixed at 100 ºC. (a-1) – (d-1) Corresponding zoom-in images.

S2.3.1. Post-annealing duration optimization: LSV

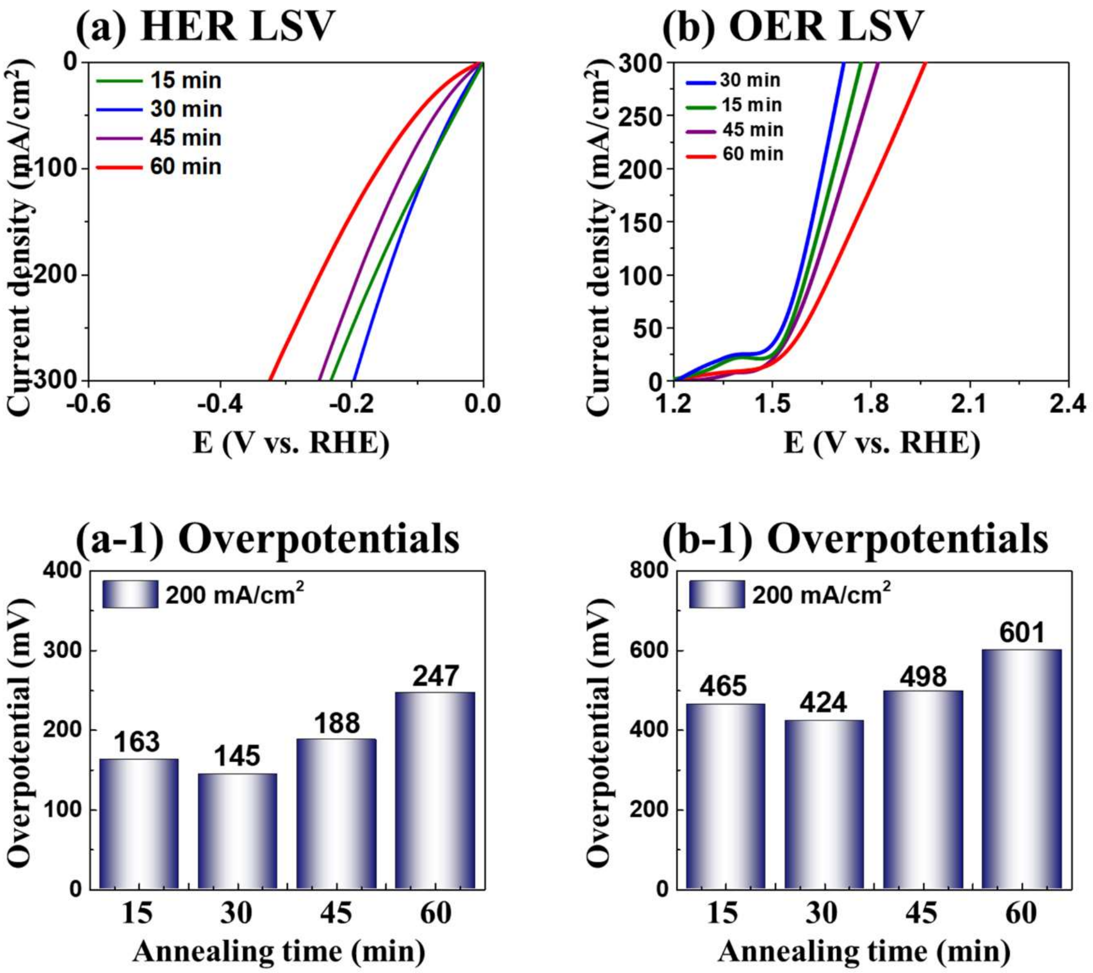

(a-2)

Best electrode	Overpotential @200 mA/cm²
30 mins	145 mV

(b-2)

Best electrode	Overpotential @200 mA/cm²
30 mins	424 mV

Figure S22: HER/OER performance of CoFeBP electrodes with post-annealing time variation. (a) & (b) HER/OER LSV results. (a-1) & (b-1) Corresponding overpotential values at 200 mA/cm^2. (a-2) & (b-2) HER/OER overpotential values of the best electrode at 200 mA/cm^2.

S2.3.2. Post-annealing temperature optimization

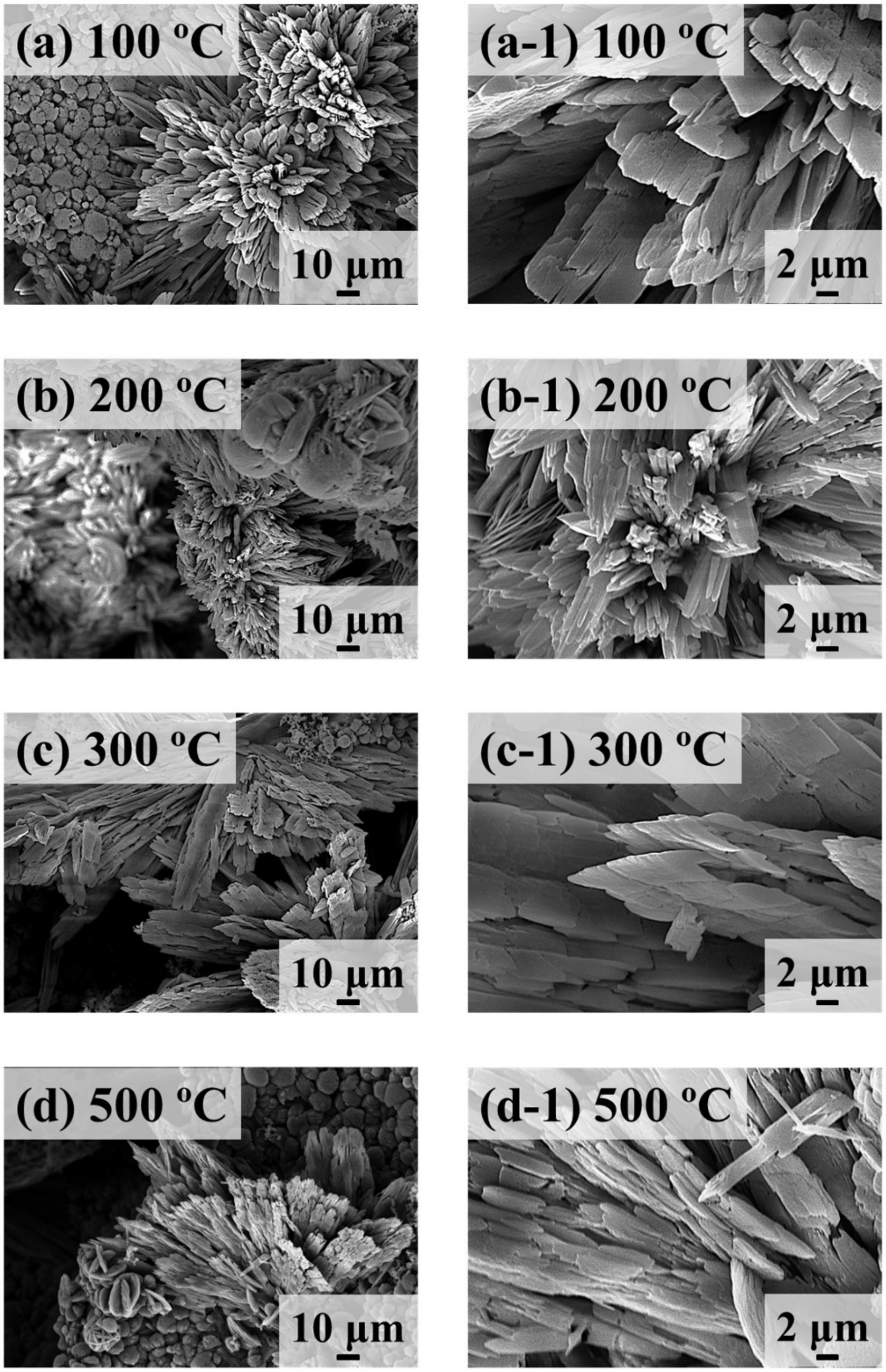

Figure S23: (a) – (d) SEM images of hybrid CoFeBP electrodes with post-annealing temperature variation. Annealing duration was fixed at 30 min. (a-1) – (d-1) Corresponding zoom-in images.

S2.3.2. Post-annealing temperature optimization: EDS

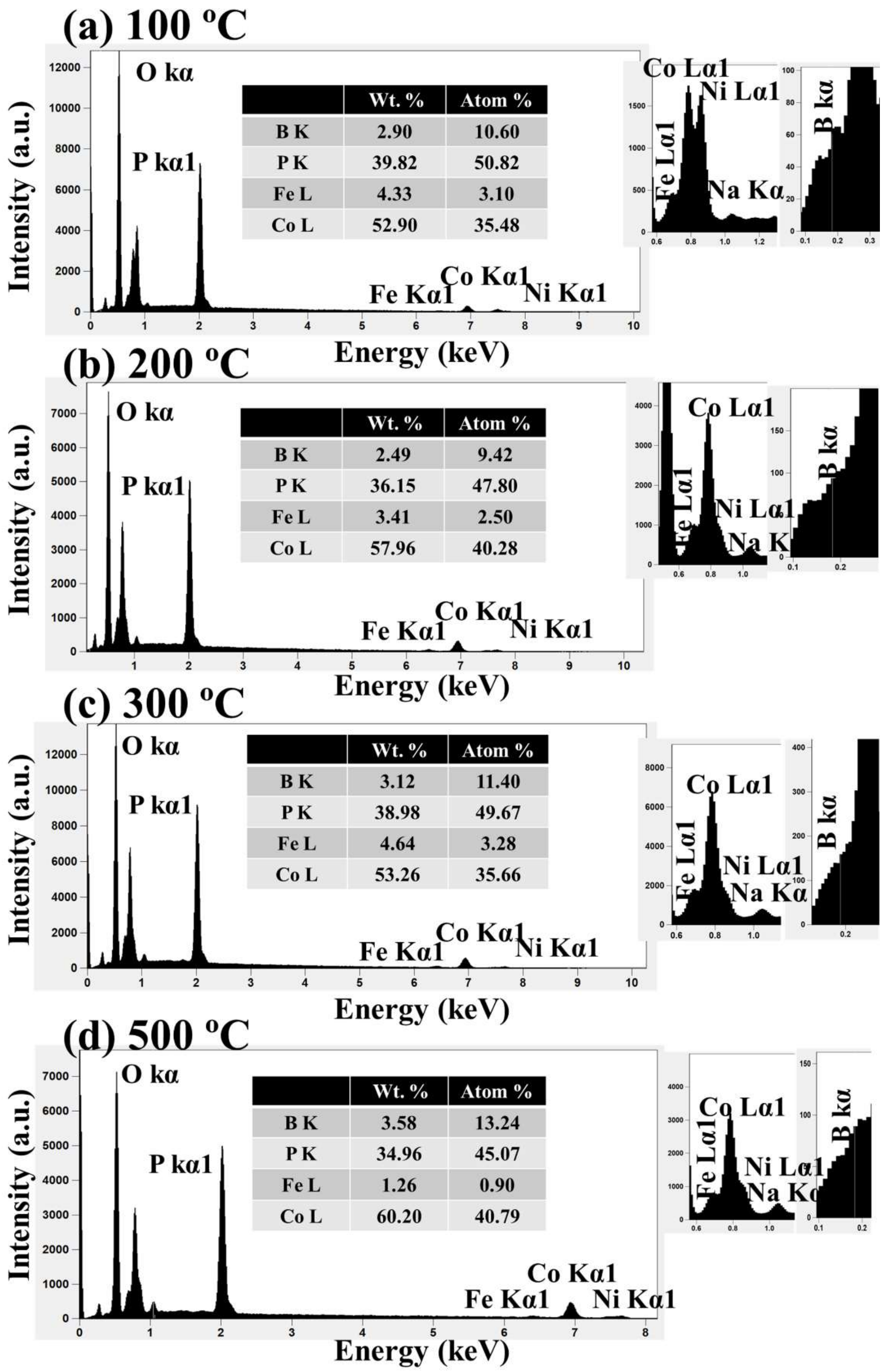

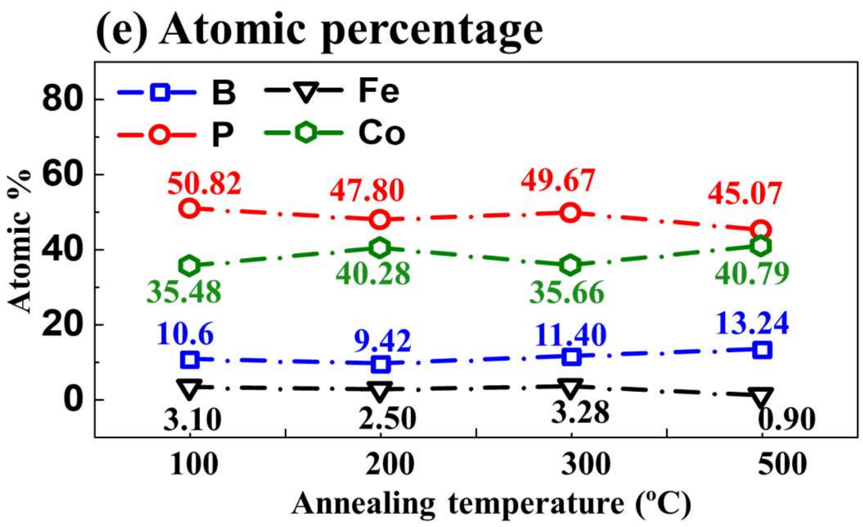

Figure S24: (a) – (d) EDS spectra of post-annealing temperature variation set. (e) Summary plot of atomic percentage changes. Generally, they showed similar atomic percentage with minor changes with post-annealing temperature variation.

S2.3.2. Post-annealing temperature optimization: LSV

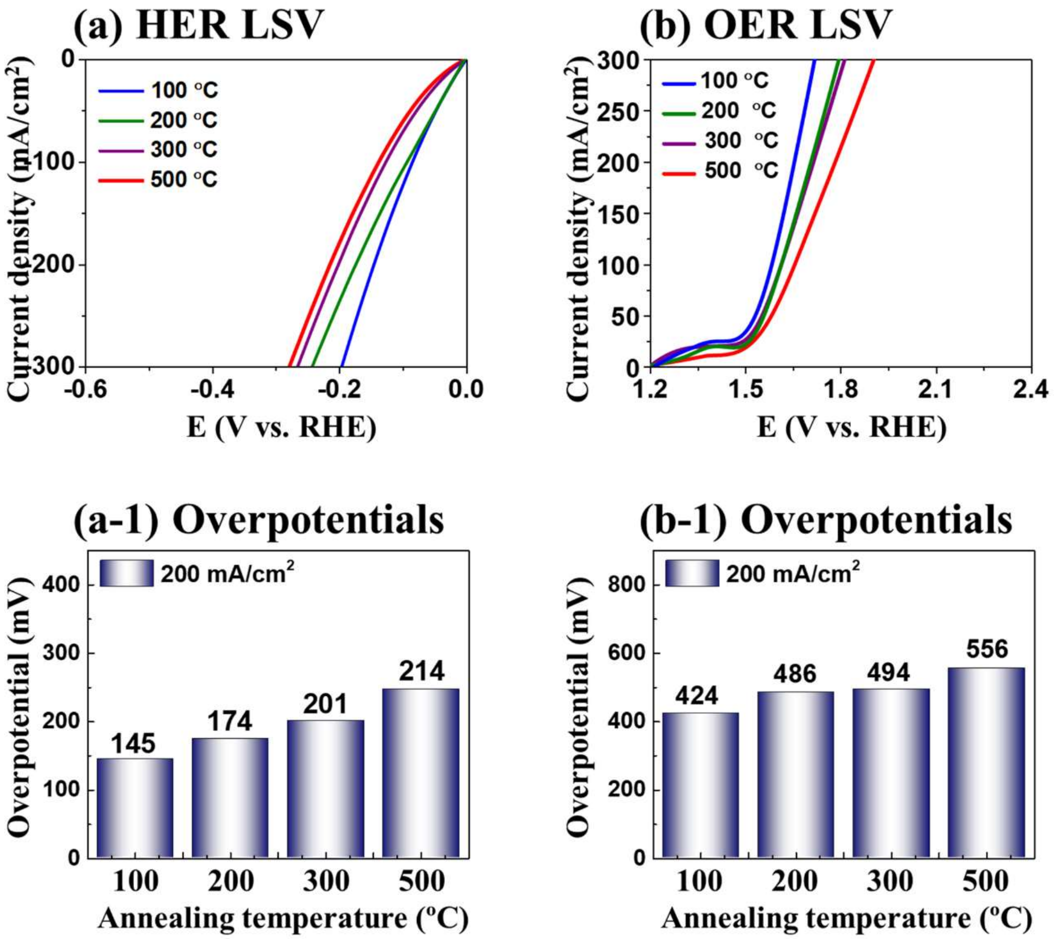

(a-2)

Best electrode	Overpotential @200 mA/cm²
100 °C	145 mV

(b-2)

Best electrode	Overpotential @200 mA/cm²
100 °C	424 mV

Figure S25: HER/OER performance of CoFeBP electrodes with post-annealing time variation. (a) & (b) HER/OER LSV results. (a-1) & (b-1) Corresponding overpotential values at 200 mA/cm^2. (a-2) & (b-2) HER/OER overpotential values of the best electrode at 200 mA/cm^2.

S2.3.2. Post-annealing temperature optimization: HER CV

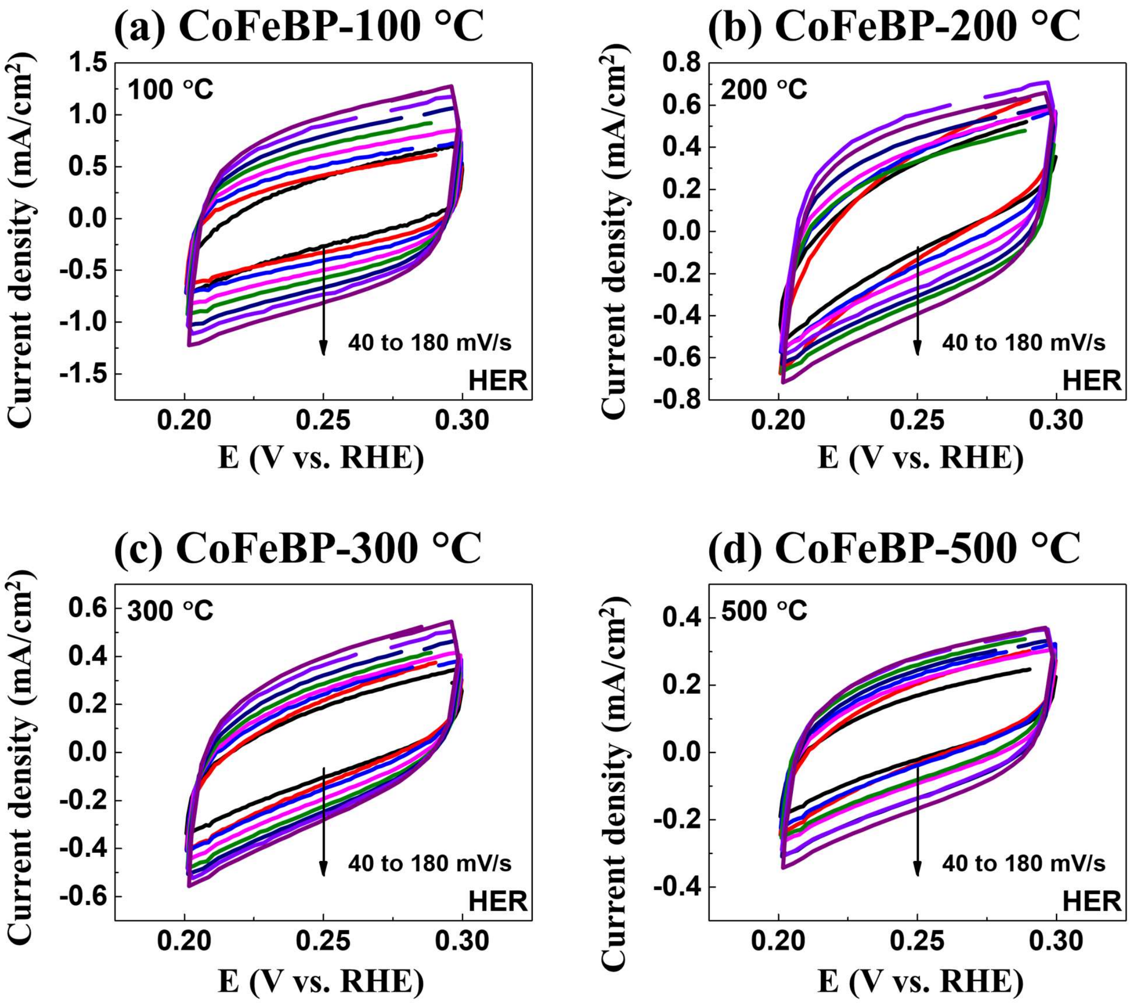

Figure S26: (a) – (d) HER CV curves of CoFeBP electrodes in the post-annealing temperature variation set. The scan rate changes from 40 mV/s to 180 mW/s. More details can be found in S1.2.

S2.3.2. Post-annealing temperature optimization: OER CV

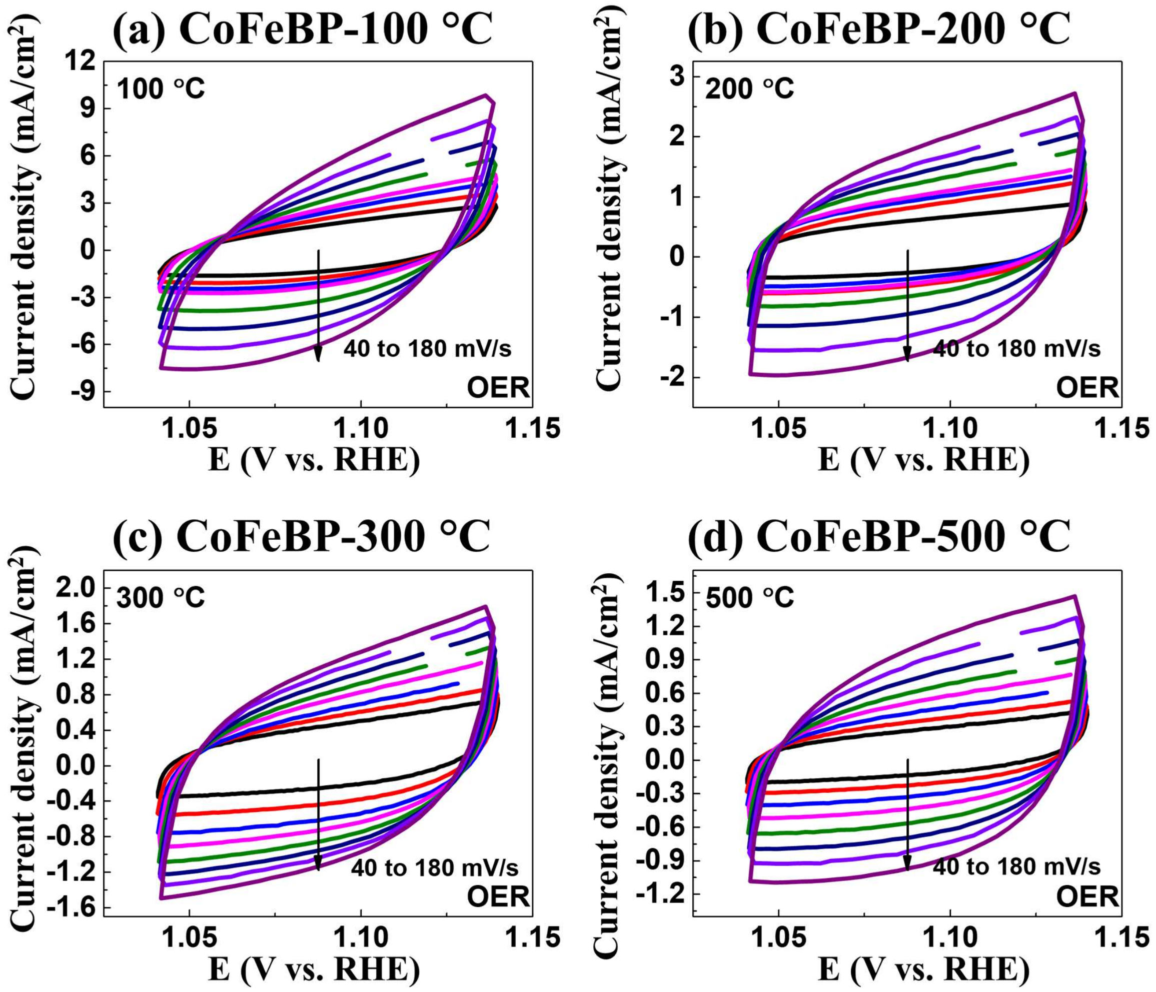

Figure S27: (a) – (d) OER CV curves of CoFeBP electrodes in the post-annealing temperature variation set. The scan rate changes from 40 mV/s to 180 mW/s. More details can be found in S1.2.

S2.3.2. Post-annealing temperature optimization: C_{dl}

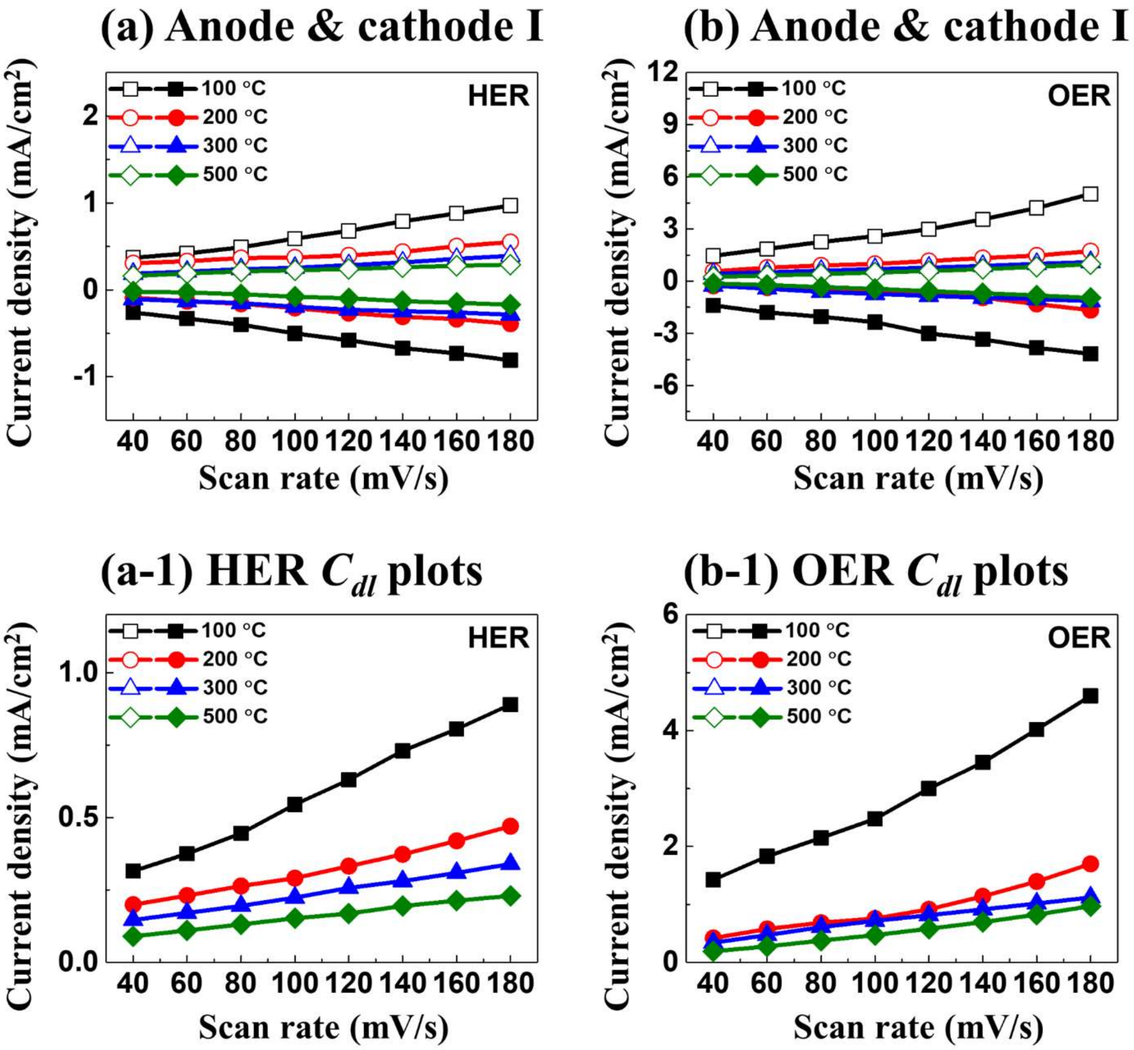

Figure S28: (a) – (b) HER/OER anodic and cathodic current density as a function of scan rate. (a-1) – (b-1) HER/OER C_{dl} plots. More details can be found in S1.2.

S2. CoFeBP MFB electrocatalysts

S2.1. Substrate and other necessary characterizations

S2.1.1. Bare Ni foam characterization

S2.1.2. LSV at different scan rates with the best CoFeBP

S2.1.3. EIS voltage variation of the best CoFeBP MFBs

S2.1.4. Pt/C (HER) reference electrode

S2.1.5. RuO_2 (OER) reference electrode

S2.2. Reaction parameter and precursor optimizations

S2.2.1. Fabrication steps of CoFeBP MFB

S2.2.2. Reaction duration optimization

S2.2.3. Reaction temperature optimization

S2.2.4. B-P concentration optimization

S2.2.5. Co-Fe concentration optimization

S2.2.6. Urea concentration optimization

S2.2.7. Urea- Ammonium fluoride concentration optimization

S2.3. Post-annealing optimization

S2.3.1. Post-annealing time optimization

S2.3.2. Post-annealing temperature optimization

S2.4. Before and after post-annealing analysis of CoFeBP MFB

S2.4.1. Before and after post-annealing: Raman

S2.4.2. Before and after post-annealing: XRD

S2.4.3. Before and after post-annealing: EIS

S2.4.4. Before and after post-annealing: HER/OER LSV

S2.4.1. Before and after post-annealing: Raman

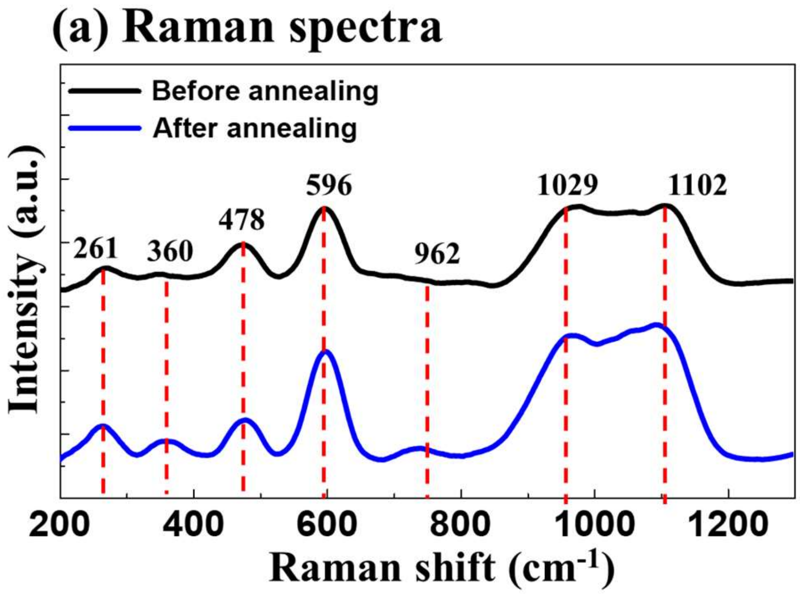

Figure S29: (a) Raman analysis of the best CoFeBP electrodes before and after post-annealing. More details can be found in S1.6.

S2.4.2. Before and after post-annealing: XRD

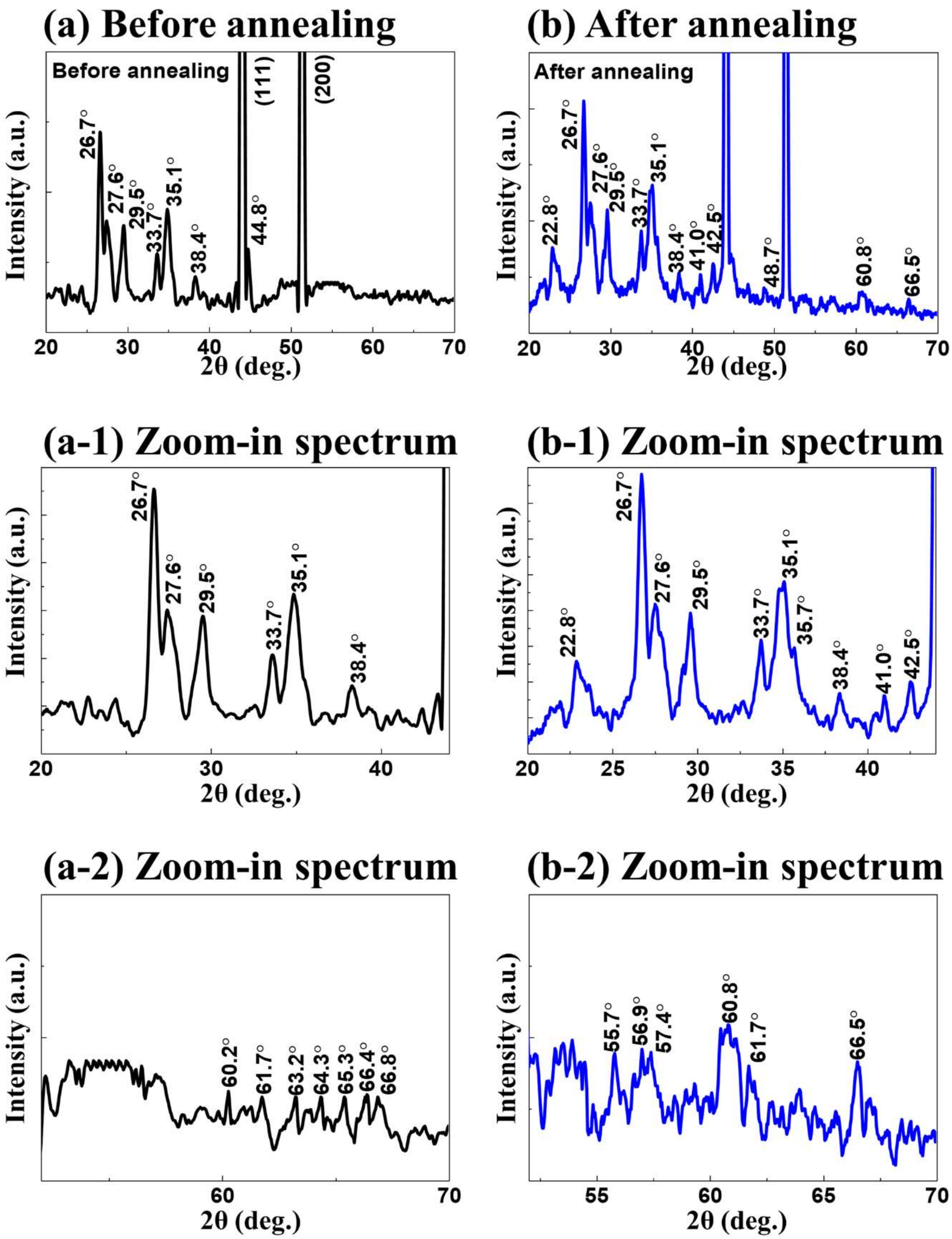

Figure S30: (a) & (b) XRD analysis of the best CoFeBP electrodes before and after post-annealing. (a-1) – (a-2), (b-1) – (b-2) Corresponding zoom-in spectra. More details can be found in S1.7.

S2.4.3. Before and after post-annealing: EIS

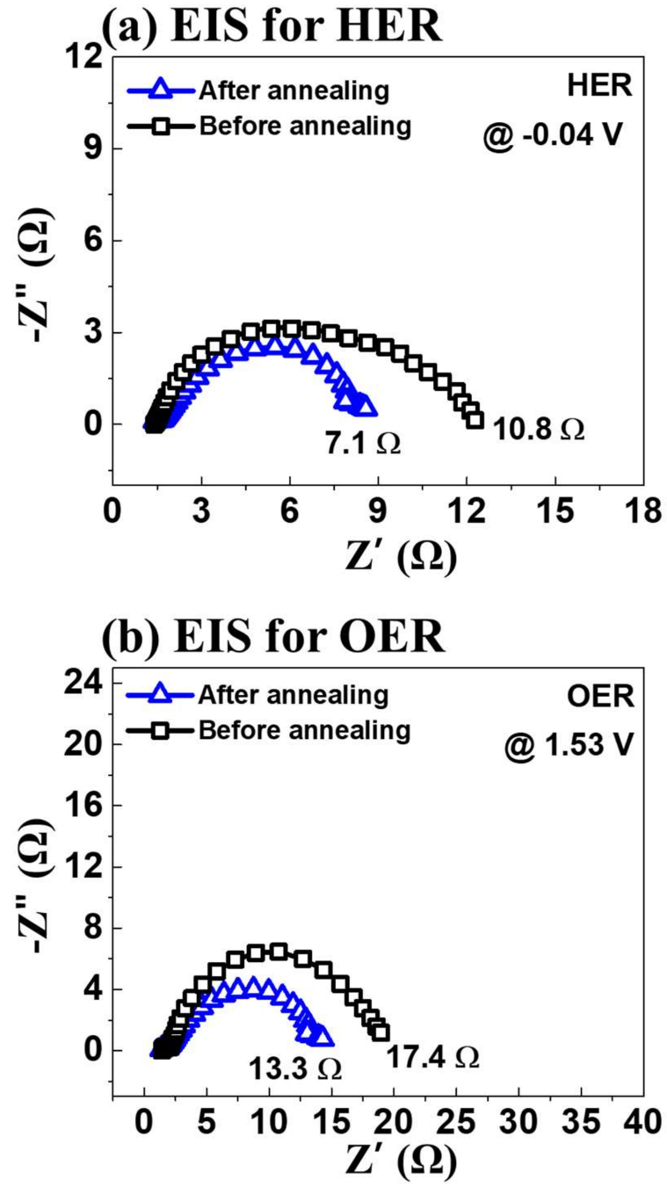

Figure S31: (a) & (b) HER/OER EIS of the best CoFeBP electrodes before and after post-annealing.

S2.4.4. Before and after post-annealing: HER/OER LSV

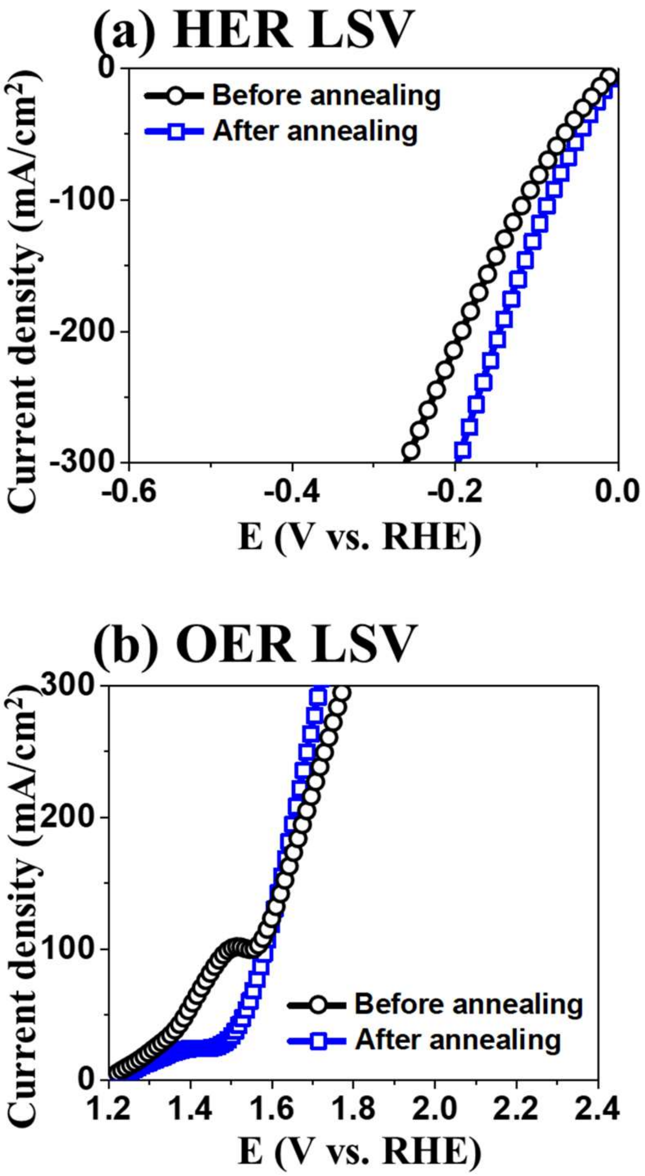

Figure S32: (a) & (b) HER and OER LSV activities of the best CoFeBP electrodes before and after post-annealing.

S3. Analysis on the best CoFeBP MFB

S3.1. XRD spectra of CoFeBP and related compounds

S3.2. XRD PDF cards of related compounds

S3.3. CV measurement in the same range of OER

S3.4. Water-gas displacement for Faradaic efficiency

S3.5. Faradaic efficiency of the best CoFeBP MFBs

S3.6. 3-E CA and LSV comparison

S3.7. 2-E CA and LSV comparison

S3.8. 2-E stabiltiy test in 1 M and 6 M KOH

S3.9. 2-E LSV in sea and river waters

S3.10. 2-E CA and LSV comparison in seawater + 1M KOH

S3.1. XRD spectra of CoFeBP and related compounds

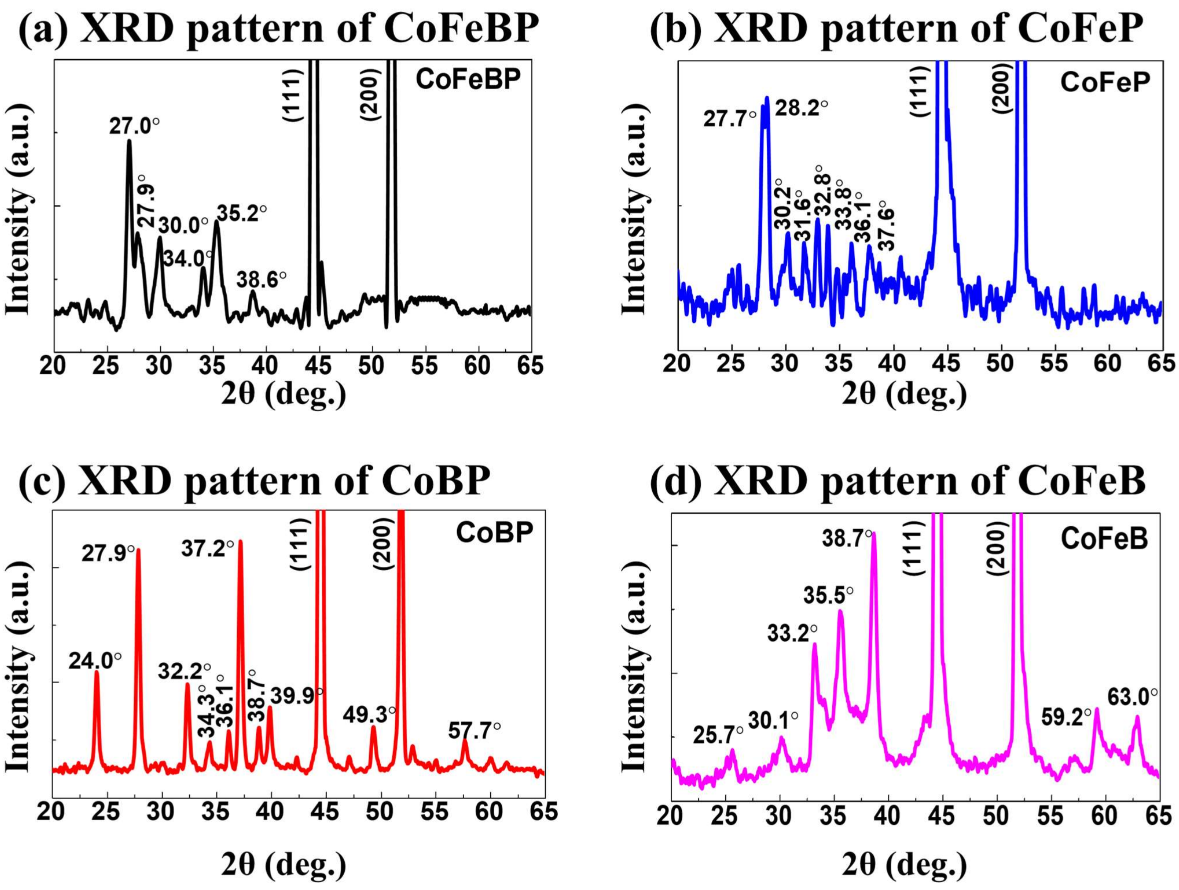

Figure S33: (a) – (d) XRD spectra of CoFeBP, CoFeP, CoBP and CoFeB electrodes.

S3.2. XRD PDF cards of related compounds

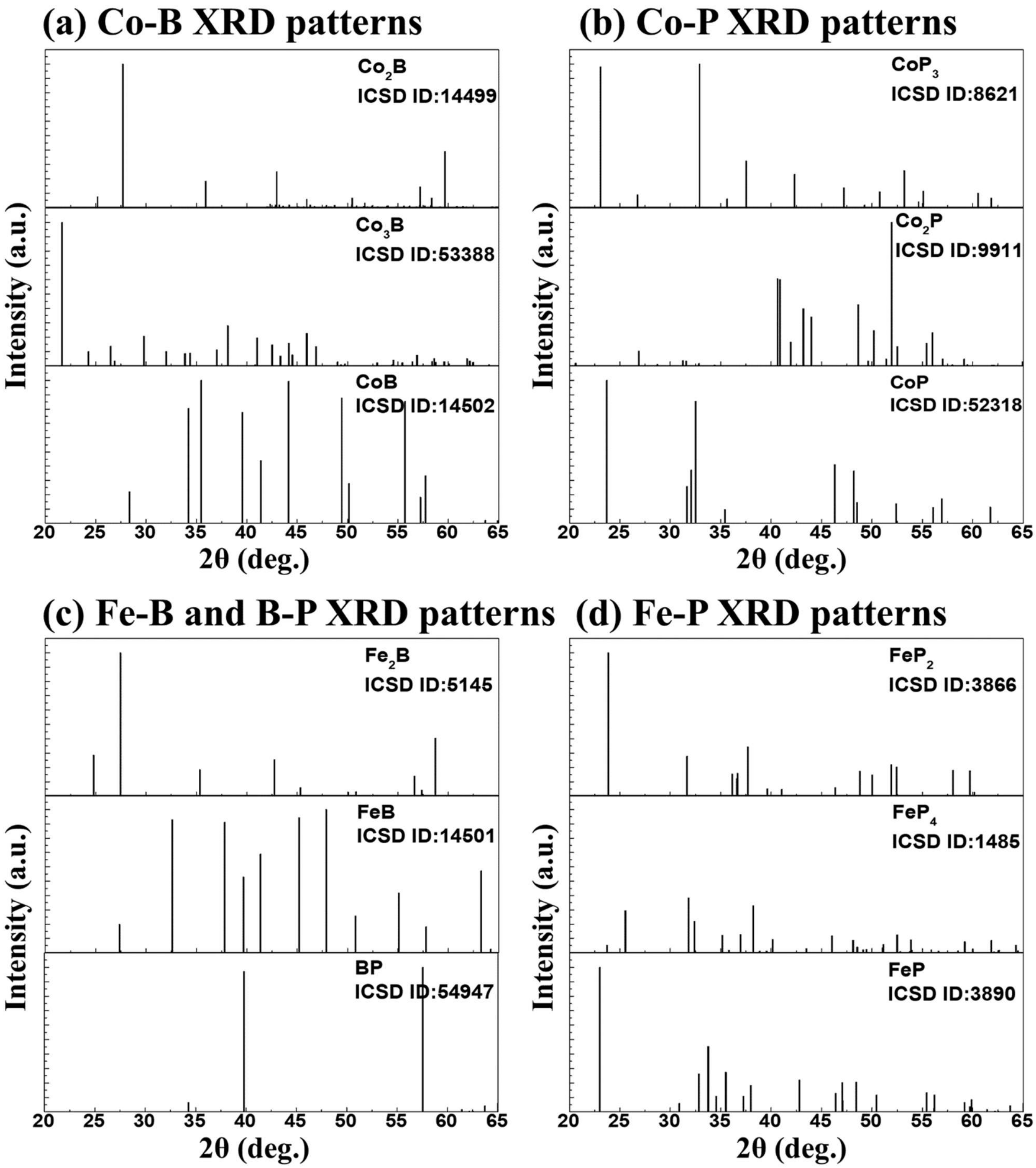

Figure S34: (a) – (d) Related PDF cards. (http://icsd.kisti.re.kr/icsd/icsd_chemistry.jsp)

S3.3. CV measurement in the same range of OER

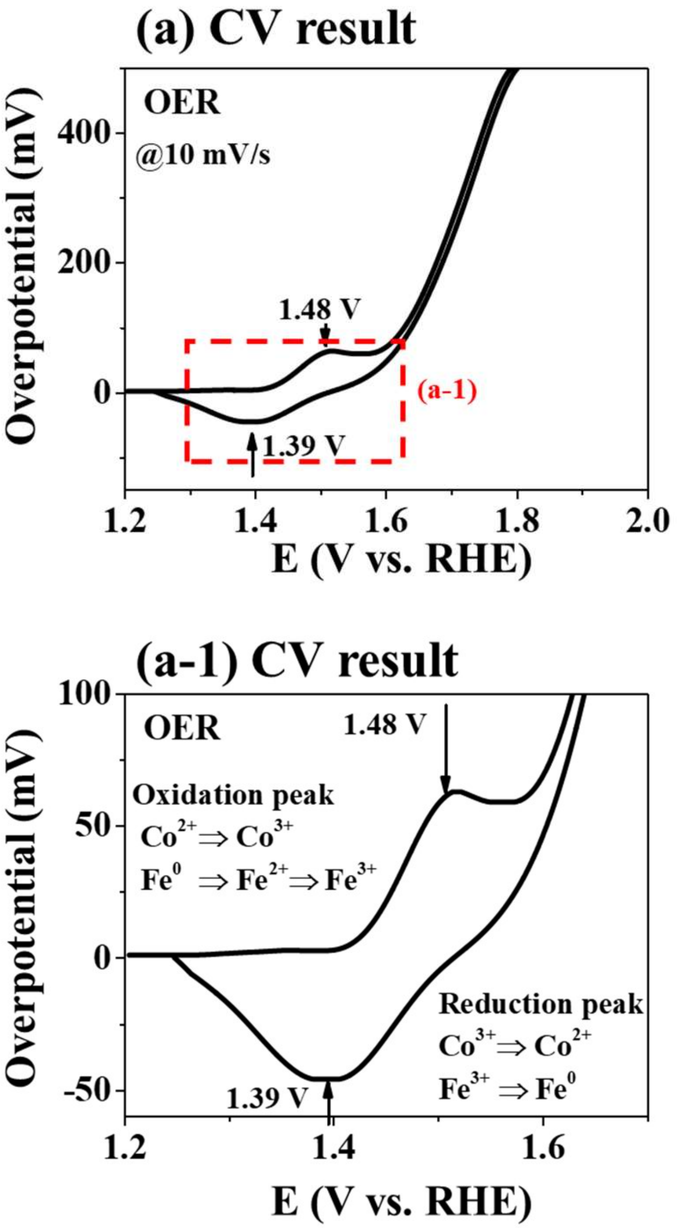

Figure S35: (a) Cyclic voltammetry (CV) measurement of Co_{90}-Fe_{10}-B_{50}-P_{50} MFB electrode in the same scanning range as OER. (a-1) Zoom-in plot of oxidation and reduction peaks.

S3.4. Water-gas displacement for Faradaic efficiency

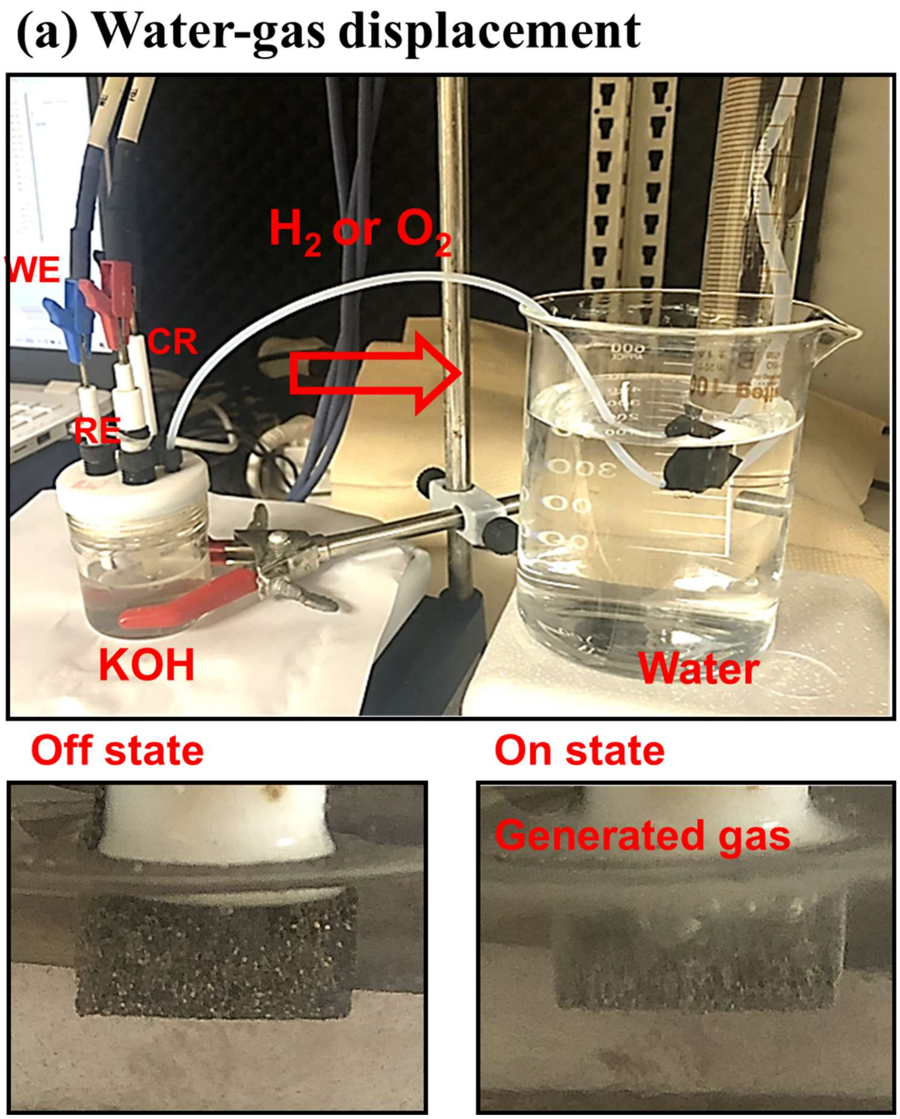

Figure S36: (a) Water-gas displacement to collect the generated H_2 and O_2 by 3-E HER/OER operations. More details can be found in S1.9.

S3.5. Faradaic efficiency of the best CoFeBP MFBs

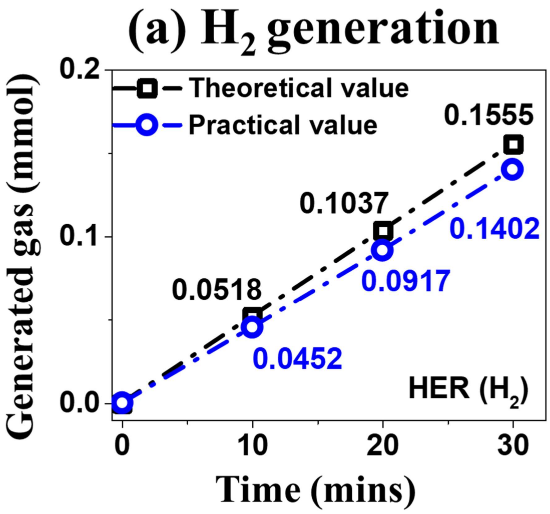

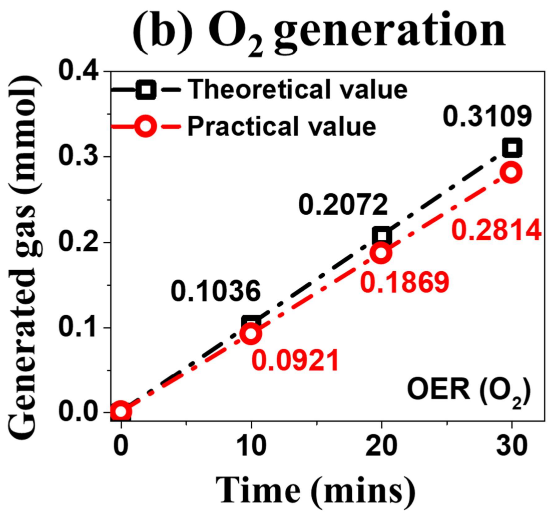

Figure S37: (a) – (b) Comparison of theoretical values and measured H_2 and O_2 in different reaction time. More details can be found in S1.9.

S3.6. 3-E CA and LSV comparison

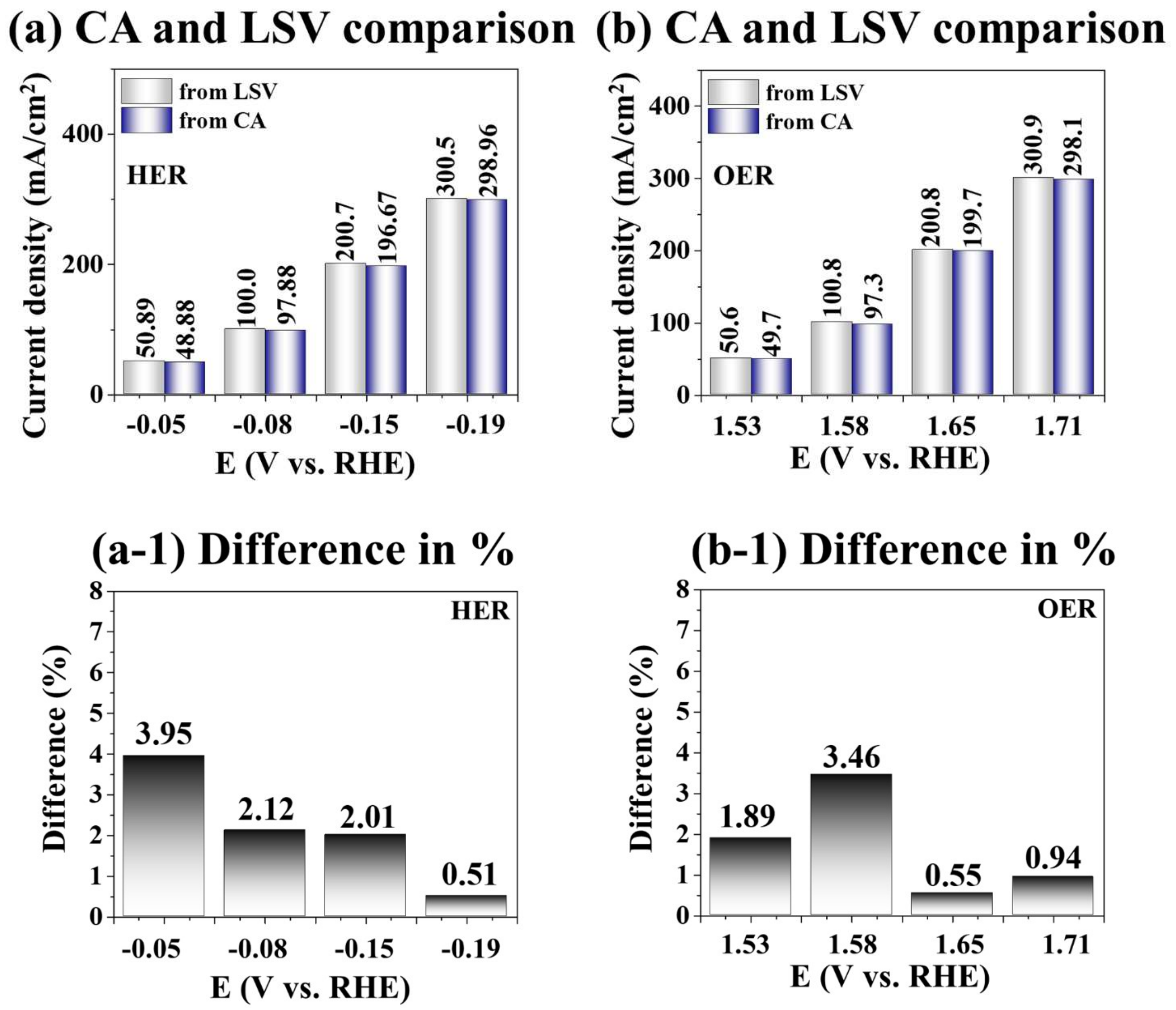

Figure S38: (a) – (b) HER/OER 3-E current density comparisons of CA and LSV. (a-1) – (b-1) Difference in percentage.

S3.7. 2-E CA and LSV comparison

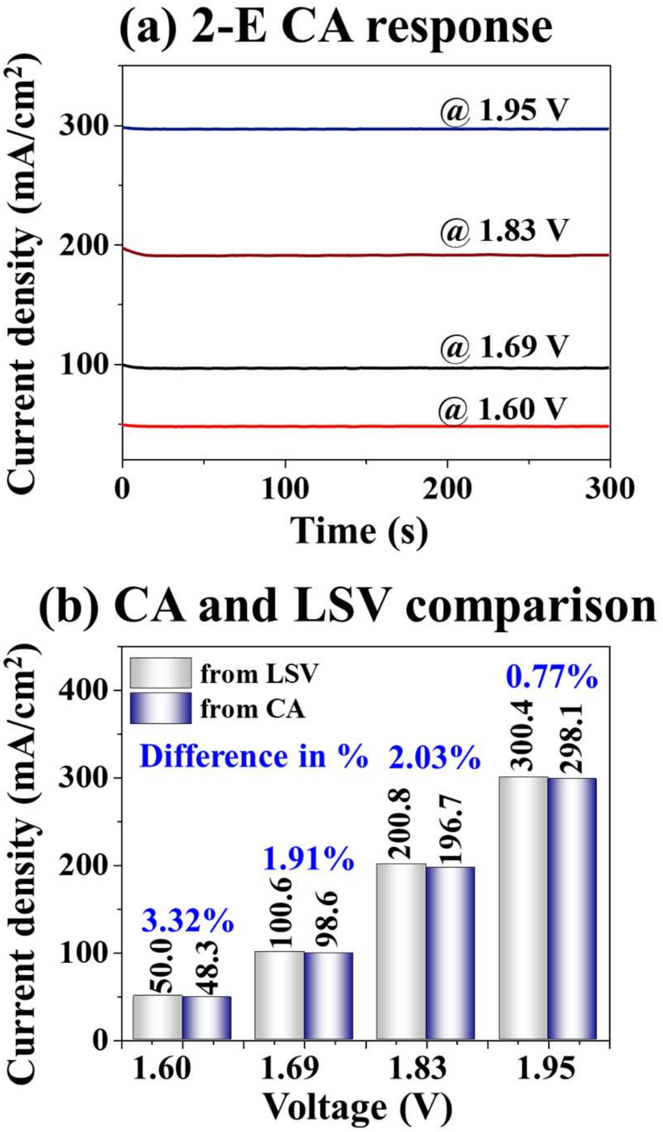

Figure S39: (a) CA response of the best CoFeBP MFBs in 1M KOH. (b) Comparison of 2-E current density of CA and LSV.

S3.8. 2-E stabiltiy test in 1 M and 6 M KOH

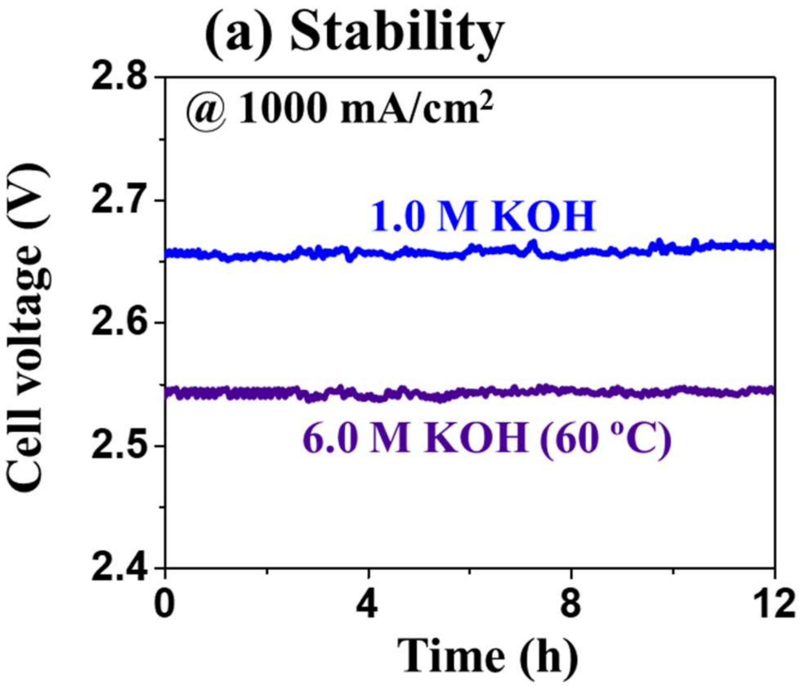

Figure S40: (a) 2-E stability test of CoFeBP || CoFeBP MFBs. The 1.0 M KOH stability was performed at room temperature at 1,000 mA/cm^2 for 12 hrs. The 6.0 M KOH stability was performed at 60 ºC at 1,000 mA/cm^2 for 12 hrs.

S3.9. 2-E LSV in sea and river waters

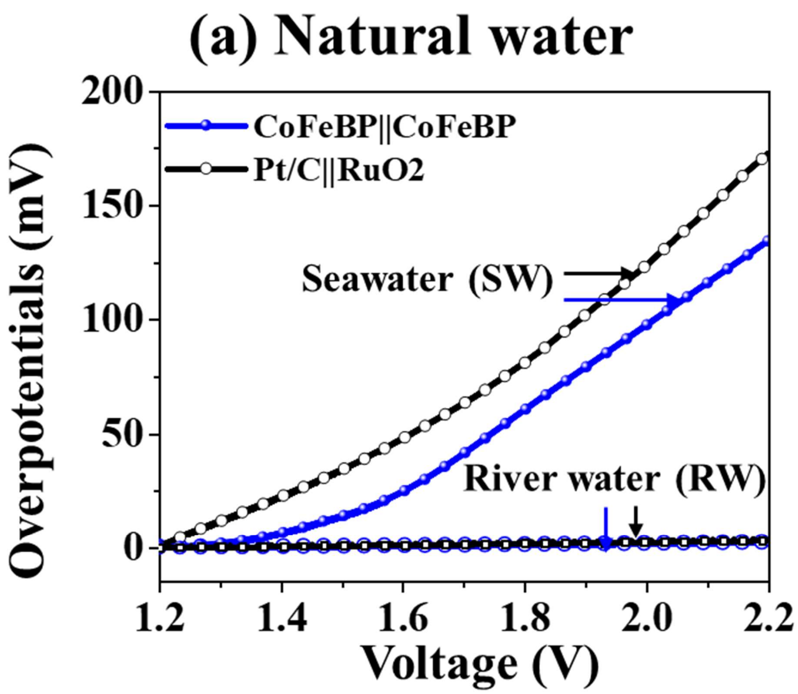

Figure S41: (a) LSV results of CoFeBP || CoFeBP MFBs in sea and river waters. While naggable water splitting (WS) was observed in river water (RW), higher WS was observed in the sea water (SW) by the CoFeBP MFBs. General conductivity of RW is ~ 1 milli-siemens per centimeter (mS/cm) and it reaches ~ 50 mS/cm in SW. Notably, in SW, due to the existence of Cl^- ions, the chlorine evolution reaction (CER) is inevitable since the required energy for the formation of hypochlorite is comparable to OER, ($Cl^- + 2OH^- = ClO^- + H_2O + 2e^-$), which will compete with oxygen generation reaction and corrode the electrode.[48] The obtained results were comparable to the reference system, meaning that the MBF CoFeBP catalyst presented superior OER and chloride corrosion resistance, which can effectively suppress the CER.

S3.10. 2-E CA and LSV comparison in seawater + 1M KOH

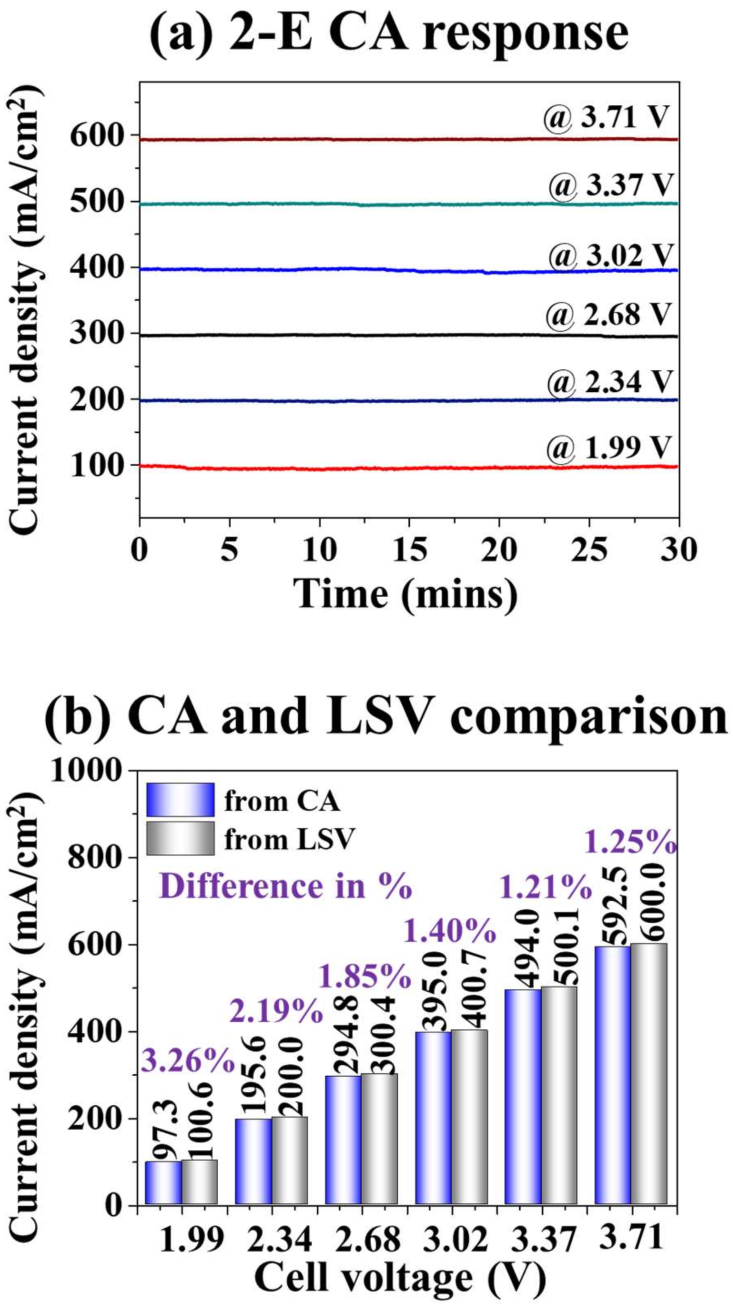

Figure S42: (a) CA response in seawater + 1M KOH of the best CoFeBP electrode. (b) Comparison of the current density of LSV and CA.

S4. After stability test

S4.1. After stability test: SEM

S4.2. After stability test: Raman

S4.3. After stability test: LSV

S4.1. After stability test: SEM

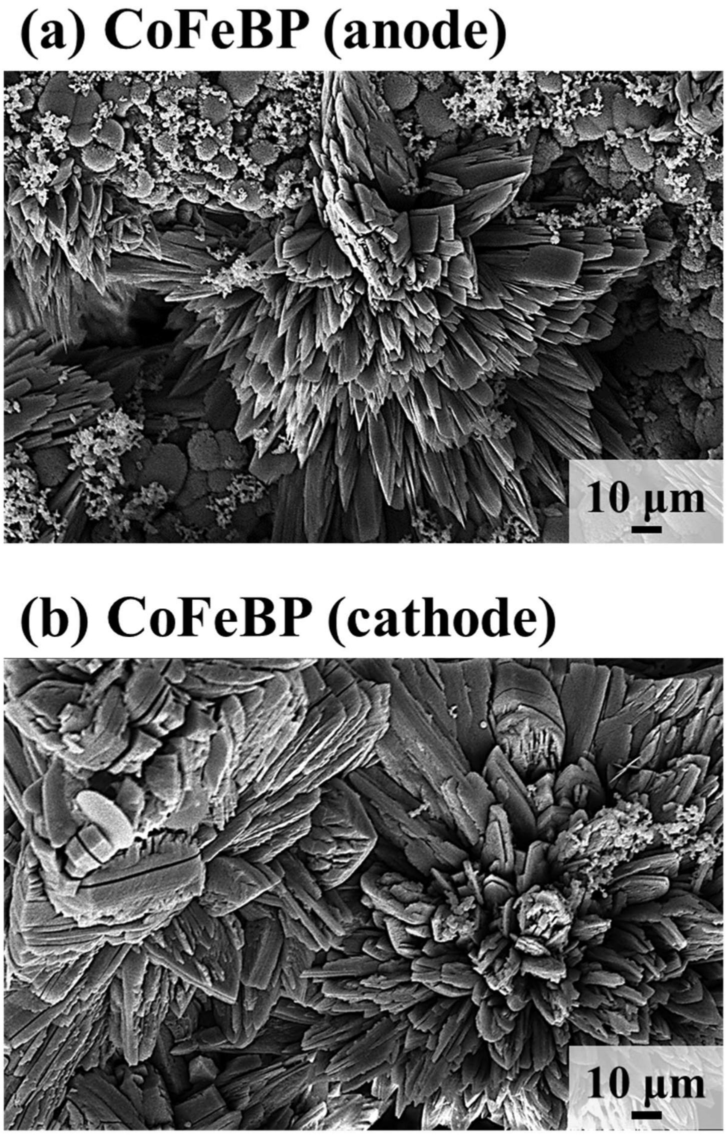

Figure S43: (a) – (b) SEM images of CoFeBP MFBs after 2-E stability test at 1,000 mA/cm^2 in 1.0 M KOH for 12-hrs.

S4.2. After stability test: Raman

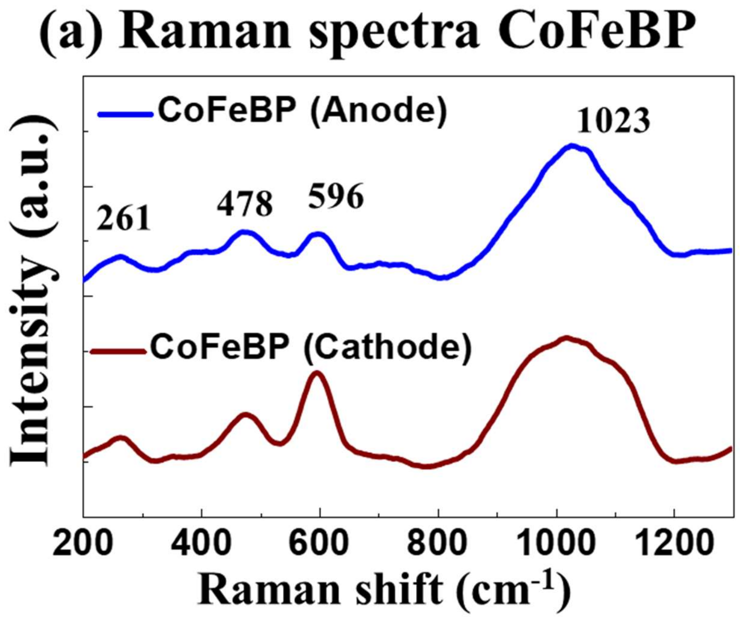

Figure S44: (a) Raman spectra of anode and cathode CoFeBP MFBs after 12-hr stability test. More details can be found in S1.6.

S4.3. After stability test: LSV

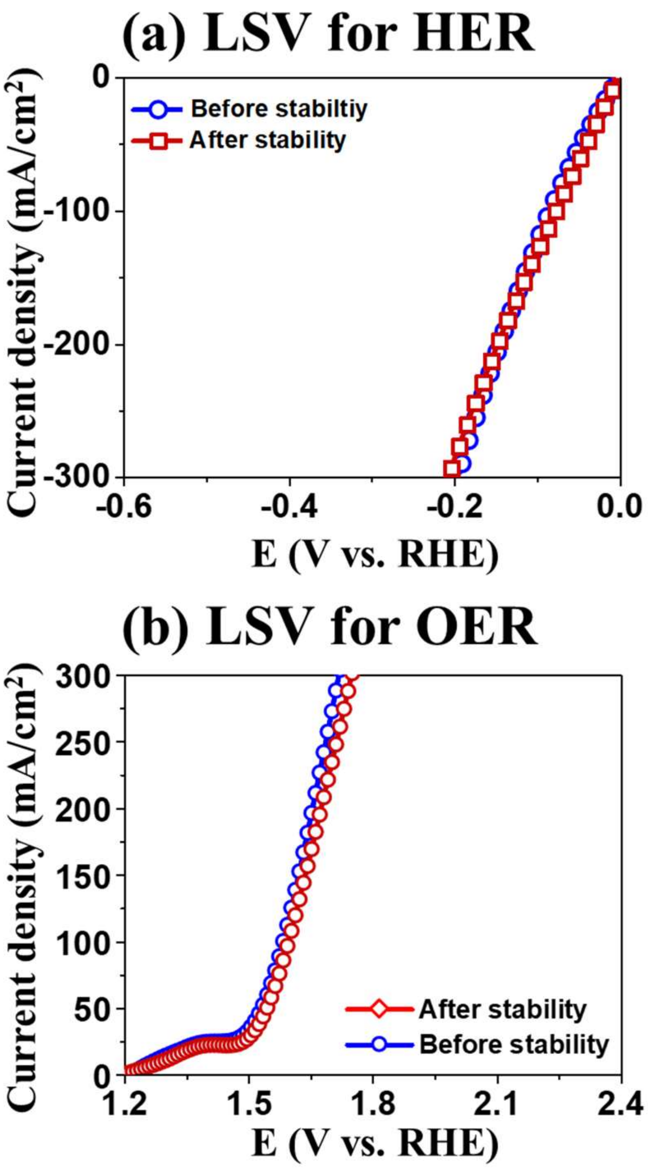

Figure S45: (a) – (b) HER and OER activities of CoFeBP MFBs after 12-hr stability test

High-resolution XPS spectrum of O 1s

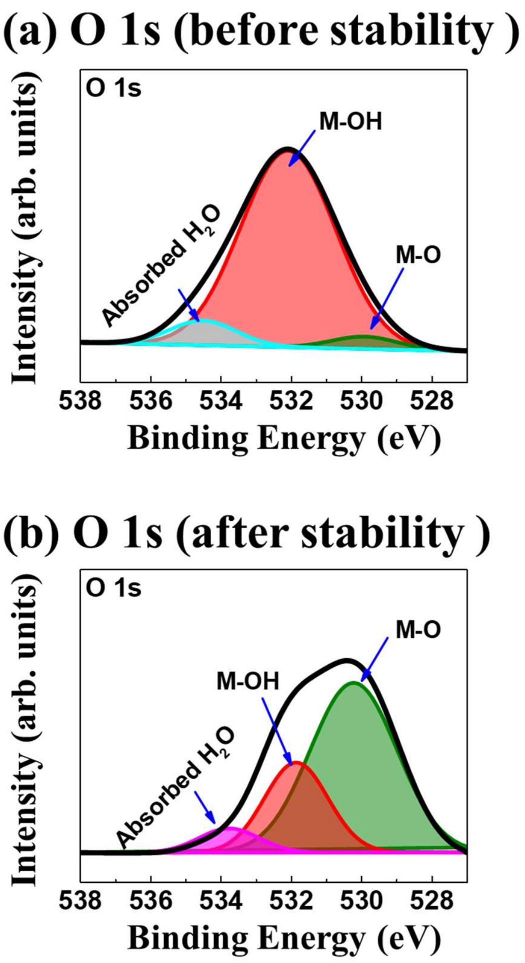

Figure S46: XPS spectrum of O 1s (a) before and (b) after stability test.

Table S1: TOF comparison of electrocatalysts for HER and OER.

Electrocatalyst	HER TOF ($site^{-1}$ s^{-1})	OER TOF ($site^{-1}$ s^{-1})	References
$Cu_{1.96}S/Co_9S_8$	0.014 @η=99 mV	0.124 @η=200 mV	[49]
Fe-$Co_{1.11}Te_2$@NCNTF	0.02 @η=100 mV	0.035 @η=300 mV	[50]
CoMnBP/CF	0.059 @η=200 mV	0.001 @η=200 mV	[33]
Co-Ni_3N	0.1459 @η= 290 mV	0.0134@η=300 mV	[51]
Ni_2V-MOFs@NF	0.186 @η=300 mV	1.58 @η=300 mV	[52]
CoFeBP	0.1932 @η=400 mV	0.101 @ η=800 mV	This work
CoPx/CNTs	0.420 @η=80 mV	0.11 @η=300 mV	[53]
$Co_3O_4/CoFe_2O_4$@NF	-	0.090 @η=300 mV	[54]
HP-Ru/C	5.33 @η= 100 mV	-	[55]
Ru@Cr-FeMOF		0.218 @η=250 mV	[56]
CoFe-LDH/I^-	-	0.2327 @η=310 mV	[57]

SI References

1. Browne, M.P.; Vasconcelos, J.M.; Coelho, J.; O'Brien, M.; Rovetta, A.A.; McCarthy, E.K.; Nolan, H.; Duesberg, G.S.; Nicolosi, V.; Colavita, P.E.; et al. Improving the Performance of Porous Nickel Foam for Water Oxidation Using Hydrothermally Prepared Ni and Fe Metal Oxides. *Sustain. Energy Fuels* **2017**, *1*, 207–216, doi:10.1039/C6SE00032K.
2. Anantharaj, S.; Ede, S.R.; Karthick, K.; Sam Sankar, S.; Sangeetha, K.; Karthik, P.E.; Kundu, S. Precision and Correctness in the Evaluation of Electrocatalytic Water Splitting: Revisiting Activity Parameters with a Critical Assessment. *Energy Environ. Sci.* **2018**, *11*, 744–771, doi:10.1039/c7ee03457a.
3. Feng, R.; Zhu, Q.; Chu, M.; Jia, S.; Zhai, J.; Wu, H.; Wu, P.; Han, B. Electrodeposited Cu–Pd Bimetallic Catalysts for the Selective Electroreduction of CO 2 to Ethylene. *Green Chem.* **2020**, *22*, 7560–7565.
4. Anantharaj, S.; Noda, S.; Driess, M.; Menezes, P.W. The Pitfalls of Using Potentiodynamic Polarization Curves for Tafel Analysis in Electrocatalytic Water Splitting. *ACS Energy Lett.* **2021**, *6*, 1607–1611, doi:10.1021/acsenergylett.1c00608.
5. Han, A.; Chen, H.; Sun, Z.; Xu, J.; Du, P. High Catalytic Activity for Water Oxidation Based on Nanostructured Nickel Phosphide Precursors. *Chem. Commun.* **2015**, *51*, 11626–11629.
6. Burse, S.; Kulkarni, R.; Mandavkar, R.; Habib, M.A.; Lin, S.; Chung, Y.-U.; Jeong, J.-H.; Lee, J. Vanadium-Doped FeBP Microsphere Croissant for Significantly Enhanced Bi-Functional HER and OER Electrocatalyst. *Nanomaterials* 2022, *12*.
7. Habib, M.A.; Mandavkar, R.; Burse, S.; Lin, S.; Kulkarni, R.; Patil, C.S.; Jeong, J.-H.; Lee, J. Design of Boron-Based Ternary W3CoB3 Electrocatalyst for the Improved HER and OER Performances. *Mater. Today Energy* **2022**, 101021, doi:https://doi.org/10.1016/j.mtener.2022.101021.
8. Wang, G.; Hua, C.; Chen, W.; Fan, H.; Feng, P.; Zhu, Y. Intriguing 3D Micro-Flower Structure of Co1.11Te2 Deposited on Te Nanosheets Showing an Efficient Bifunctional Electrocatalytic Property for Overall Water Splitting. *Electrochim. Acta* **2023**, *447*, 142133, doi:https://doi.org/10.1016/j.electacta.2023.142133.
9. Mandavkar, R.; Habib, M.A.; Lin, S.; Kulkarni, R.; Burse, S.; Jeong, J.-H.; Lee, J. Electron Enriched Ternary NiMoB Electrocatalyst for Improved Overall Water Splitting: Better Performance as Compared to the Pt/C || RuO2 at High Current Density. *Appl. Mater. Today* **2022**, *29*, 101579, doi:https://doi.org/10.1016/j.apmt.2022.101579.

10. Sun, H.; Meng, J.; Jiao, L.; Cheng, F.; Chen, J. A Review of Transition-Metal Boride/Phosphide-Based Materials for Catalytic Hydrogen Generation from Hydrolysis of Boron-Hydrides. *Inorg. Chem. Front.* **2018**, *5*, 760–772, doi:10.1039/C8QI00044A.
11. Liu, Y.; Yu, Y.; Mu, Z.; Wang, Y.; Ali, U.; Jing, S.; Xing, S. Urea-Assisted Enhanced Electrocatalytic Activity of MoS2-Ni3S2for Overall Water Splitting. *Inorg. Chem. Front.* **2020**, *7*, 3588–3597, doi:10.1039/d0qi00634c.
12. Chen, M.-T.; Duan, J.-J.; Feng, J.-J.; Mei, L.-P.; Jiao, Y.; Zhang, L.; Wang, A.-J. Iron, Rhodium-Codoped Ni2P Nanosheets Arrays Supported on Nickel Foam as an Efficient Bifunctional Electrocatalyst for Overall Water Splitting. *J. Colloid Interface Sci.* **2022**, *605*, 888–896, doi:https://doi.org/10.1016/j.jcis.2021.07.101.
13. Xu, T.; Yang, L.; Li, J.; Usoltseva, N.; An, V.; Jin, X.; Zhang, C.; Zhang, X.; Liu, B. NH4F-Induced Morphology Control of CoP Nanostructures to Enhance the Hydrogen Evolution Reaction. *Inorg. Chem.* **2021**, *60*, 10781–10790, doi:10.1021/acs.inorgchem.1c01484.
14. Yang, G.; Jiao, Y.; Yan, H.; Xie, Y.; Wu, A.; Dong, X.; Guo, D.; Tian, C.; Fu, H. Interfacial Engineering of MoO2-FeP Heterojunction for Highly Efficient Hydrogen Evolution Coupled with Biomass Electrooxidation. *Adv. Mater.* **2020**, *32*, 2000455, doi:https://doi.org/10.1002/adma.202000455.
15. Feng, J.; Wang, X.; Zhang, D.; Wang, Y.; Wang, J.; Pi, M.; Zhou, H.; Li, J.; Chen, S. Porous Mn-Doped CoP3 Nanowires as a Janus Electrocatalyst for Efficient Overall Water Splitting in Alkaline Solution. *J. Electrochem. Soc.* **2018**, *165*, F1323, doi:10.1149/2.0781816jes.
16. Yu, X.; Yu, Z.-Y.; Zhang, X.-L.; Li, P.; Sun, B.; Gao, X.; Yan, K.; Liu, H.; Duan, Y.; Gao, M.-R.; et al. Highly Disordered Cobalt Oxide Nanostructure Induced by Sulfur Incorporation for Efficient Overall Water Splitting. *Nano Energy* **2020**, *71*, 104652, doi:https://doi.org/10.1016/j.nanoen.2020.104652.
17. Wang, X.; Huang, H.; Qian, J.; Li, Y.; Shen, K. Intensified Kirkendall Effect Assisted Construction of Double-Shell Hollow Cu-Doped CoP Nanoparticles Anchored by Carbon Arrays for Water Splitting. *Appl. Catal. B Environ.* **2023**, *325*, 122295, doi:https://doi.org/10.1016/j.apcatb.2022.122295.
18. Wang, L.; Lu, X.; Han, C.; Lu, R.; Yang, S.; Song, X. Electrospun Hollow Cage-like α-Fe2O3 Microspheres: Synthesis, Formation Mechanism, and Morphology-Preserved Conversion to Fe Nanostructures. *CrystEngComm* **2014**, *16*, 10618–10623, doi:10.1039/C4CE01485E.
19. Li, X.; Xiao, L.; Zhou, L.; Xu, Q.; Weng, J.; Xu, J.; Liu, B. Adaptive Bifunctional Electrocatalyst of Amorphous CoFe Oxide @ 2D Black Phosphorus for Overall Water Splitting. *Angew. Chemie* **2020**, *132*, 21292–21299,

doi:https://doi.org/10.1002/ange.202008514.

20. Diaz-Morales, O.; Ferrus-Suspedra, D.; Koper, M.T.M. The Importance of Nickel Oxyhydroxide Deprotonation on Its Activity towards Electrochemical Water Oxidation. *Chem. Sci.* **2016**, *7*, 2639–2645, doi:10.1039/C5SC04486C.
21. Huang, C.; Zhang, B.; Wu, Y.; Ruan, Q.; Liu, L.; Su, J.; Tang, Y.; Liu, R.; Chu, P.K. Experimental and Theoretical Investigation of Reconstruction and Active Phases on Honeycombed Ni3N-Co3N/C in Water Splitting. *Appl. Catal. B Environ.* **2021**, *297*, 120461, doi:https://doi.org/10.1016/j.apcatb.2021.120461.
22. Peng, Y.; Mak, C.H.; Kai, J.-J.; Du, M.; Ji, L.; Yuan, M.; Zou, X.; Shen, H.-H.; Santoso, S.P.; Colmenares, J.C.; et al. Recent Progress on Post-Synthetic Treatments of Photoelectrodes for Photoelectrochemical Water Splitting. *J. Mater. Chem. A* **2021**, *9*, 26628–26649, doi:10.1039/D1TA05935A.
23. Tian, L.; Li, Z.; Xu, X.; Zhang, C. Advances in Noble Metal (Ru, Rh, and Ir) Doping for Boosting Water Splitting Electrocatalysis. *J. Mater. Chem. A* **2021**, *9*, 13459–13470, doi:10.1039/D1TA01108A.
24. Zhang, H.; Lee, J.S. Hybrid Microwave Annealing Synthesizes Highly Crystalline Nanostructures for (Photo)Electrocatalytic Water Splitting. *Acc. Chem. Res.* **2019**, *52*, 3132–3142, doi:10.1021/acs.accounts.9b00353.
25. Guo, C.; Shi, Y.; Lu, S.; Yu, Y.; Zhang, B. Amorphous Nanomaterials in Electrocatalytic Water Splitting. *Chinese J. Catal.* **2021**, *42*, 1287–1296, doi:https://doi.org/10.1016/S1872-2067(20)63740-8.
26. Wang, H.; Zou, H.; Liu, Y.; Liu, Z.; Sun, W.; Lin, K.A.; Li, T.; Luo, S. Ni2P Nanocrystals Embedded Ni-MOF Nanosheets Supported on Nickel Foam as Bifunctional Electrocatalyst for Urea Electrolysis. *Sci. Rep.* **2021**, *11*, 1–11, doi:10.1038/s41598-021-00776-8.
27. Lin, S.; Habib, M.A.; Mandavkar, R.; Kulkarni, R.; Burse, S.; Chung, Y.-U.; Liu, C.; Wang, Z.; Lin, S.; Jeong, J.-H.; et al. Higher Water-Splitting Performance of Boron-Based Porous CoMnB Electrocatalyst over the Benchmarks at High Current in 1 m KOH and Real Sea Water. *Adv. Sustain. Syst.* **2022**, *6*, 2200213, doi:https://doi.org/10.1002/adsu.202200213.
28. Kim, T.; Roy, S.B.; Moon, S.; Yoo, S.; Choi, H.; Parale, V.G.; Kim, Y.; Lee, J.; Jun, S.C.; Kang, K. Highly Dispersed Pt Clusters on F-Doped Tin (IV) Oxide Aerogel Matrix: An Ultra-Robust Hybrid Catalyst for Enhanced Hydrogen Evolution. *ACS Nano* **2022**.
29. Liu, Y.; Li, H.; Gong, S.; Chen, Y.; Xie, R.; Wu, Q.; Tao, J.; Meng, F.; Zhao, P. A Novel Non-Enzymatic Electrochemical Biosensor Based on the Nanohybrid of Bimetallic PdCu Nanoparticles/Carbon Black for Highly Sensitive Detection of H2O2 Released from Living

Cells. *Sensors Actuators, B Chem.* **2019**, *290*, 249–257, doi:10.1016/j.snb.2019.03.129.

30. Sultan, S.; Ha, M.; Kim, D.Y.; Tiwari, J.N.; Myung, C.W.; Meena, A.; Shin, T.J.; Chae, K.H.; Kim, K.S. Superb Water Splitting Activity of the Electrocatalyst Fe3Co (PO4) 4 Designed with Computation Aid. *Nat. Commun.* **2019**, *10*, 1–9.
31. Wang, S.; Lu, A.; Zhong, C.-J. Hydrogen Production from Water Electrolysis: Role of Catalysts. *Nano Converg.* **2021**, *8*, 4, doi:10.1186/s40580-021-00254-x.
32. Chastain, J.; King Jr, R.C. Handbook of X-Ray Photoelectron Spectroscopy. *Perkin-Elmer Corp.* **1992**, *40*, 221.
33. Wei, Y.; Zou, P.; Yue, Y.; Wang, M.; Fu, W.; Si, S.; Wei, L.; Zhao, X.; Hu, G.; Xin, H.L. One-Pot Synthesis of B/P-Codoped Co-Mo Dual-Nanowafer Electrocatalysts for Overall Water Splitting. *ACS Appl. Mater. Interfaces* **2021**, *13*, 20024–20033, doi:10.1021/acsami.1c01341.
34. Yang, J.; An, Y.; Guo, K.; Ren, X.; Jiang, B. Nitrogen Doped FeCoNiS Nanoparticles on N, S-Co-Doped Vertical Graphene as Bifunctional Electrocatalyst for Water Splitting. *Int. J. Hydrogen Energy* **2023**, *48*, 4143–4157, doi:https://doi.org/10.1016/j.ijhydene.2022.10.216.
35. Surendran, S.; Jesudass, S.C.; Janani, G.; Kim, J.Y.; Lim, Y.; Park, J.; Han, M.-K.; Cho, I.S.; Sim, U. Sulphur Assisted Nitrogen-Rich CNF for Improving Electronic Interactions in Co-NiO Heterostructures Toward Accelerated Overall Water Splitting. *Adv. Mater. Technol.* **2023**, *8*, 2200572, doi:https://doi.org/10.1002/admt.202200572.
36. Miao, L.; Sui, L.; Shen, X.; Yang, D.; Huang, H.; Kuang, Y. Realizing High Performance Bifunctional Energy Storage Devices and Electrocatalytic Water Splitting Catalysts through Regulated Interface Engineering of ZnCo2O4@Co3O4 Nanosheets. *CrystEngComm* **2023**, *25*, 4812–4821, doi:10.1039/D3CE00534H.
37. Tsai, F.-T.; Deng, Y.-T.; Pao, C.-W.; Chen, J.-L.; Lee, J.-F.; Lai, K.-T.; Liaw, W.-F. The HER/OER Mechanistic Study of an FeCoNi-Based Electrocatalyst for Alkaline Water Splitting. *J. Mater. Chem. A* **2020**, *8*, 9939–9950, doi:10.1039/D0TA01877E.
38. Li, C.-F.; Zhao, J.-W.; Xie, L.-J.; Wu, J.-Q.; Li, G.-R. Fe Doping and Oxygen Vacancy Modulated Fe-Ni5P4/NiFeOH Nanosheets as Bifunctional Electrocatalysts for Efficient Overall Water Splitting. *Appl. Catal. B Environ.* **2021**, *291*, 119987, doi:https://doi.org/10.1016/j.apcatb.2021.119987.
39. Du, Y.; Li, Z.; Liu, H.; Qiao, S.; Chen, Y.; Zhu, Z.; Tang, Y.; Liu, C. Scalable Oxygen-Assisted-Fe2+ Etching Approach towards Amorphous/Crystalline Structure Fe-Ni2P Nanoarray for Efficient Water Splitting. *J. Alloys Compd.* **2023**, *936*, 168073, doi:https://doi.org/10.1016/j.jallcom.2022.168073.

40. Yang, N.; Tian, S.; Feng, Y.; Hu, Z.; Liu, H.; Tian, X.; Xu, L.; Hu, C.; Yang, J. Introducing High-Valence Iridium Single Atoms into Bimetal Phosphides toward High-Efficiency Oxygen Evolution and Overall Water Splitting. *Small* **2023**, *19*, 2207253, doi:https://doi.org/10.1002/smll.202207253.
41. Dai, Z.; Du, X.; Zhang, X. The Synthesis of Ni-Co-Fe-Se@NiCo-LDH Nanoarrays on Ni Foam as Efficient Overall Water Splitting Electrocatalyst. *J. Alloys Compd.* **2023**, *946*, 169451, doi:https://doi.org/10.1016/j.jallcom.2023.169451.
42. Teng, W.; Huo, M.; Sun, Z.; Yang, W.; Zheng, X.; Ding, C.; Zhang, S. FeCoNi Sulfides Derived from in Situ Sulfurization of Precursor Oxides as Oxygen Evolution Reaction Catalyst. *Front. Chem.* **2020**, *8*, 334.
43. Hao, W.; Yao, D.; Xu, Q.; Wang, R.; Zhang, C.; Guo, Y.; Sun, R.; Huang, M.; Chen, Z. Highly Efficient Overall-Water Splitting Enabled via Grafting Boron-Inserted Fe-Ni Solid Solution Nanosheets onto Unconventional Skeleton. *Appl. Catal. B Environ.* **2021**, *292*, 120188, doi:https://doi.org/10.1016/j.apcatb.2021.120188.
44. Chen, M.; Kitiphatpiboon, N.; Feng, C.; Zhao, Q.; Abudula, A.; Ma, Y.; Yan, K.; Guan, G. Tuning Octahedron Sites in MnFe2O4 Spinel by Boron Doping for Highly Efficient Seawater Splitting. *Appl. Catal. B Environ.* **2023**, *330*, 122577, doi:https://doi.org/10.1016/j.apcatb.2023.122577.
45. Guo, F.; Li, W.; Liu, Y.; Chen, Q.; Zhong, Q. Heterogeneous Fe-Doped NiCoP–MoO3 Efficient Electrocatalysts for Overall Water Splitting. *Langmuir* **2023**, *39*, 1042–1050, doi:10.1021/acs.langmuir.2c02678.
46. Shit, S.; Bolar, S.; Murmu, N.C.; Kuila, T. An Account of the Strategies to Enhance the Water Splitting Efficiency of Noble-Metal-Free Electrocatalysts. *J. Energy Chem.* **2021**, *59*, 160–190, doi:https://doi.org/10.1016/j.jechem.2020.10.022.
47. Lei, B.; Xu, D.; Wei, B.; Xie, T.; Xiao, C.; Jin, W.; Xu, L. In Situ Synthesis of α-Fe2O3/Fe3O4 Heterojunction Photoanode via Fast Flame Annealing for Enhanced Charge Separation and Water Oxidation. *ACS Appl. Mater. Interfaces* **2021**, *13*, 4785–4795, doi:10.1021/acsami.0c19927.
48. Wang, Y.; Yu, W.; Zhou, B.; Xiao, W.; Wang, J.; Wang, X.; Xu, G.; Li, B.; Li, Z.; Wu, Z.; et al. Corrosive Engineering Assisted in Situ Construction of an Fe–Ni-Based Compound for Industrial Overall Water-Splitting under Large-Current Density in Alkaline Freshwater and Seawater Media. *J. Mater. Chem. A* **2023**, *11*, 1886–1893, doi:10.1039/D2TA07586E.
49. Xiao, Y.; Shen, Y.; Su, D.; Zhang, S.; Yang, J.; Yan, D.; Fang, S.; Wang, X. Engineering Cu1.96S/Co9S8 with Sulfur Vacancy and Heterostructure as an Efficient Bifunctional

Electrocatalyst for Water Splitting. *J. Mater. Sci. Technol.* **2023**, *154*, 1–8, doi:https://doi.org/10.1016/j.jmst.2022.12.042.

50. He, B.; Wang, X.-C.; Xia, L.-X.; Guo, Y.-Q.; Tang, Y.-W.; Zhao, Y.; Hao, Q.-L.; Yu, T.; Liu, H.-K.; Su, Z. Metal-Organic Framework-Derived Fe-Doped Co1.11Te2 Embedded in Nitrogen-Doped Carbon Nanotube for Water Splitting. *ChemSusChem* **2020**, *13*, 5239–5247, doi:https://doi.org/10.1002/cssc.202001434.
51. Zhu, C.; Wang, A.-L.; Xiao, W.; Chao, D.; Zhang, X.; Tiep, N.H.; Chen, S.; Kang, J.; Wang, X.; Ding, J.; et al. In Situ Grown Epitaxial Heterojunction Exhibits High-Performance Electrocatalytic Water Splitting. *Adv. Mater.* **2018**, *30*, 1705516, doi:https://doi.org/10.1002/adma.201705516.
52. Lv, J.; Liu, P.; Yang, F.; Xing, L.; Wang, D.; Chen, X.; Gao, H.; Huang, X.; Lu, Y.; Wang, G. 3D Hydrangea Macrophylla-like Nickel–Vanadium Metal–Organic Frameworks Formed by Self-Assembly of Ultrathin 2D Nanosheets for Overall Water Splitting. *ACS Appl. Mater. Interfaces* **2020**, *12*, 48495–48510, doi:10.1021/acsami.0c11722.
53. Huang, C.; Ouyang, T.; Zou, Y.; Li, N.; Liu, Z.-Q. Ultrathin NiCo2Px Nanosheets Strongly Coupled with CNTs as Efficient and Robust Electrocatalysts for Overall Water Splitting. *J. Mater. Chem. A* **2018**, *6*, 7420–7427, doi:10.1039/C7TA11364A.
54. Ma, Y.; Zhou, Y.; Wang, C.; Gao, B.; Li, J.; Zhu, M.; Wu, H.; Zhang, C.; Qin, Y. Photothermal–Magnetic Synergistic Effects in an Electrocatalyst for Efficient Water Splitting under Optical–Magnetic Fields. *Adv. Mater.* **2023**, *35*, 2303741, doi:https://doi.org/10.1002/adma.202303741.
55. Hong, C.-B.; Li, X.; Wei, W.-B.; Wu, X.-T.; Zhu, Q.-L. Nano-Engineering of Ru-Based Hierarchical Porous Nanoreactors for Highly Efficient PH-Universal Overall Water Splitting. *Appl. Catal. B Environ.* **2021**, *294*, 120230, doi:https://doi.org/10.1016/j.apcatb.2021.120230.
56. Zhao, C.; Wang, J.; Gao, Y.; Zhang, J.; Huang, C.; Shi, Q.; Mu, S.; Xiao, Q.; Huo, S.; Xia, Z.; et al. D-Orbital Manipulated Ru Nanoclusters for High-Efficiency Overall Water Splitting at Industrial-Level Current Densities. *Adv. Funct. Mater.* **2023**, *n/a*, 2307917, doi:https://doi.org/10.1002/adfm.202307917.
57. Nagappan, S.; Karmakar, A.; Madhu, R.; Dhandapani, H.N.; Singha Roy, S.; Kundu, S. Tuning the Active Sites and Optimizing the D-Spacing Value in CoFe-LDH by Ex Situ Intercalation of Guest Anions: An Innovative Electrocatalyst for Overall Water Splitting Reaction. *Catal. Sci. Technol.* **2023**, *13*, 6377–6391, doi:10.1039/D3CY00859B.
